# Influence of Protein Glycosylation on *Campylobacter fetus* Physiology

**DOI:** 10.3389/fmicb.2020.01191

**Published:** 2020-06-17

**Authors:** Justin Duma, Harald Nothaft, Danielle Weaver, Christopher Fodor, Bernadette Beadle, Dennis Linton, Stéphane L. Benoit, Nichollas E. Scott, Robert J. Maier, Christine M. Szymanski

**Affiliations:** ^1^Department of Microbiology, University of Georgia, Athens, GA, United States; ^2^Complex Carbohydrate Research Center, University of Georgia, Athens, GA, United States; ^3^Department of Biological Sciences, University of Alberta, Edmonton, AB, Canada; ^4^School of Biological Sciences, Faculty of Biology, Medicine and Health, The University of Manchester, Manchester, United Kingdom; ^5^Department of Microbiology and Immunology, The Peter Doherty Institute, The University of Melbourne, Melbourne, VIC, Australia

**Keywords:** *Campylobacter fetus*, N-linked protein glycosylation, glycosyltransferase, proteomics, metal regulation, hydrogenase

## Abstract

*Campylobacter fetus* is commonly associated with venereal disease and abortions in cattle and sheep, and can also cause intestinal or systemic infections in humans that are immunocompromised, elderly, or exposed to infected livestock. It is also believed that *C. fetus* infection can result from the consumption or handling of contaminated food products, but *C. fetus* is rarely detected in food since isolation methods are not suited for its detection and the physiology of the organism makes culturing difficult. In the related species, *Campylobacter jejuni*, the ability to colonize the host has been linked to N-linked protein glycosylation with quantitative proteomics demonstrating that glycosylation is interconnected with cell physiology. Using label-free quantitative (LFQ) proteomics, we found more than 100 proteins significantly altered in expression in two *C. fetus* subsp. *fetus* protein glycosylation (*pgl*) mutants (*pglX* and *pglJ*) compared to the wild-type. Significant increases in the expression of the (NiFe)-hydrogenase HynABC, catalyzing H_2_-oxidation for energy harvesting, correlated with significantly increased levels of cellular nickel, improved growth in H_2_ and increased hydrogenase activity, suggesting that N-glycosylation in *C. fetus* is involved in regulating the HynABC hydrogenase and nickel homeostasis. To further elucidate the function of the *C. fetus pgl* pathway and its enzymes, heterologous expression in *Escherichia coli* followed by mutational and functional analyses revealed that PglX and PglY are novel glycosyltransferases involved in extending the *C. fetus* hexasaccharide beyond the conserved core, while PglJ and PglA have similar activities to their homologs in *C. jejuni*. In addition, the *pgl* mutants displayed decreased motility and ethidium bromide efflux and showed an increased sensitivity to antibiotics. This work not only provides insight into the unique protein N-glycosylation pathway of *C. fetus*, but also expands our knowledge on the influence of protein N-glycosylation on *Campylobacter* cell physiology.

## Introduction

**FIGURE 1:**
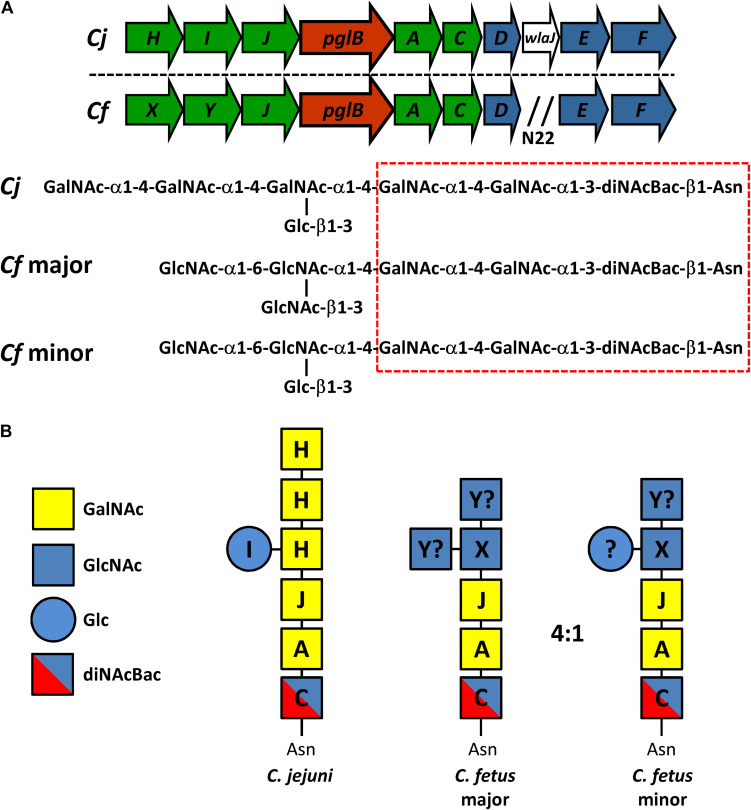
Comparison of the N-linked protein glycosylation (*pgl*) pathway in *C. jejuni* and *C. fetus*. **(A)** The N-linked glycan structures and the genetic organization of the *pgl* locus for *C. jejuni* 11168 (*Cj*) and *C. fetus fetus* ATCC 27374 (*Cf*) (according to Nothaft et al., 2012) are shown. Genes encoding glycosyltransferases are in green, the oligosaccharyltransferase gene is in red, and genes for the biosynthesis of diNAcBac are in blue. N_22_ indicates an insertion of 22 genes (between *pglD* and *pglE*) in the *Cff*-*pgl* operon. The hatched red box indicates the conserved group of sugars at the reducing end. **(B)** Similar to *Cj*, we propose that *Cf*-PglC transfers diNAcBAc which is synthesized by *Cj/Cf* PglDEF (not shown) to undecaprenyl-phosphate (Nothaft and Szymanski, 2010). Subsequently, we show that *Cf*-PglA transfers the first α1–3 linked GalNAc followed by the second α1–4 linked GalNAc residue added by *Cf*-PglJ, comparable to the *Cj* homologs. To this trisaccharide, *Cj*-PglH transfers three α1–4 linked GalNAc residues and *Cj-*PglI subsequently transfers the β1–3 linked Glc branch (Kelly et al., 2006). For *Cf*, PglX most likely transfers the first HexNAc (α1–4 linked GlcNAc), but it remains to be determined if PglY can transfer the β1–3 linked Glc or the remaining two GlcNAc residues in the major and the minor glycan forms. The question mark indicates that another enzyme outside the gene cluster could be responsible for the addition of those sugar residues.

Asparagine-linked protein glycosylation is a post-translational modification present in species from all three domains of life. The prototypical bacterial protein N-glycosylation system (referred to as *pgl*) was first identified in *Campylobacter jejuni* over two decades ago (Szymanski et al., 1999). This system utilizes five glycosyltransferases (*pglA*, *pglC*, *pglH*, *pglI*, *pglJ*) to produce the heptasaccharide GalNAc-α1,4-GalNAc-α1,4-(Glc-β1,3-)GalNAc-α1,4-GalNAc-α1,4-GalNAc-α1,3-diNAcBac-β1,N-Asn (diNAcBac is 2,4-diacetamido-2,4,6-trideoxyglucopyranose) which is attached to protein (Figure 1; Glover et al., 2005, 2006; Linton et al., 2005). The assembly of the full-length glycan occurs on the cytoplasmic side of the inner membrane through the sequential transfer of nucleotide-activated sugars onto the lipid carrier undecaprenyl-phosphate. The lipid-linked heptasaccharide is then flipped into the periplasm by the flippase PglK (Alaimo et al., 2006; Kelly et al., 2006) and transferred to the asparagine residue within the consensus sequon D/E-X_1_-N-X_2_-S/T (where X_1_, X_2_ can be any amino acid except proline) by the oligosaccharyltransferase PglB (Kowarik et al., 2006; Chen et al., 2007; Scott et al., 2011), or is released as free oligosaccharide (Nothaft et al., 2009), a process that is conserved among *Campylobacter* species (Nothaft et al., 2012). In *C. jejuni*, the conserved heptasaccharide has been found on more than 80 periplasmic and membrane-bound proteins (Scott et al., 2011; Cain et al., 2019). Mutagenesis of the *pgl* genes indicates that this glycosylation system impacts multiple cell functions including: (i) colonization of chickens and mice; (ii) adherence and invasion of epithelial cells; (iii) functionality of the multidrug efflux complex CmeABC; (iv) stability of the type IV secretion system; and (v) interactions with the immune system (Nothaft and Szymanski, 2013; Dubb et al., 2020). More specifically, two recent proteomics studies of *C. jejuni pglB* mutants have revealed multiple physiological functions associated with N-glycosylation (Abouelhadid et al., 2019; Cain et al., 2019). These include increased expression of stress response proteins, decreased survival in high temperature and osmolarity, altered metabolic activities, decreased chemotaxis, impaired efflux, and decreased nitrate reductase activity (Abouelhadid et al., 2019; Cain et al., 2019).

Orthologs of the *pgl* pathway have been found in all *Campylobacter* spp., select *Helicobacter* spp., *Desulfovibrio desulfuricans*, *Wolinella succinogenes*, *Deferribacter desulfuricans*, *Sulfurovum* sp., *Nitratiruptor* sp., and some less characterized δ- and ε-Proteobacteria (Nakagawa et al., 2007; Jervis et al., 2010; Ielmini and Feldman, 2011; Nothaft et al., 2012; Mills et al., 2016). Despite the conservation of the *pgl* pathway *per se*, different *Campylobacter* species produce N-glycans that vary in structure and composition (Jervis et al., 2012; Nothaft et al., 2012). This is particularly evident among the non-thermotolerant *Campylobacter* species which produce multiple N-linked glycoforms (Jervis et al., 2012; Nothaft et al., 2012). For instance, *Campylobacter fetus* synthesizes two distinct N-linked hexasaccharides: the major GlcNAc-α1-6-(GlcNAc-β1-3)-GlcNAc-α1-4-GalNAc-α1-4-GalNAc-α1-3-diNAcBac-β1,N-Asn and the minor GlcNAc-α1-6-(Glc-β1-3)-GlcNAc-α1-4-GalNAc-α1-4-GalNAc-α1-3-diNAcBac-β1,N-Asn at a 4:1 ratio, respectively (Nothaft et al., 2012).

*Campylobacter fetus* grows best between 25 and 37°C and consists of three subspecies: *C. fetus* subsp. *fetus* (*Cff*), *C. fetus* subsp. *venerealis* (*Cfv*), and the more recently described subspecies *C. fetus* subsp. *testudinum* (*Cft*) thought to originate from reptiles, but also associated with human infections (Patrick et al., 2013; Fitzgerald et al., 2014). *Cff* has the broadest host range and is found in cattle, sheep, reptiles, and humans (Tu et al., 2004; Wagenaar et al., 2014). In livestock, both *Cfv* and *Cff* are known to cause reproductive failure and infertility (Duncan et al., 2014), and although *Cfv* has been isolated from humans, it only causes disease in cattle (Holst et al., 1987). The majorities of human *C. fetus* infections are attributed to *Cff* and are associated with meningitis, acute diarrhea, and most commonly bacteremia (Wagenaar et al., 2014). Human infections are generally sporadic, with only a few reported outbreaks (Klein et al., 1986; Marchand-Senecal et al., 2017). Recent metagenomic analysis found *C. fetus* in 8% of feces from healthy humans, suggesting it is a possible pathobiont (Iraola et al., 2017).

In this study, we examined the role of several *C. fetus pgl*-encoded glycosyltransferases through mutagenesis and functional transfer into *Escherichia coli.* We demonstrate that the *Cff*-PglA and *Cff*-PglJ homologs have the same function as their counterparts in *C. jejuni* building the conserved GalNAc-α1,4-GalNAc-α1,3-diNAcBac reducing-end core. PglX (previously annotated as PglH1) and PglY (previously annotated as PglH2) are associated with the biosynthesis of the structurally variable region at the non-reducing end of the *Cff*-hexasaccharides. To assess the potential impact of the N-glycan truncations on other cellular functions, a label-free quantitative proteomics approach was used to examine the *Cff*-*pglJ* and *pglX* mutants. Proteomics demonstrated widespread changes in protein abundance with a notable impact on metal transport proteins, several (NiFe) hydrogenase subunits, and oxidative response proteins compared to the wild-type (WT). The results presented in this study provide new insights into the assembly and roles of N-linked glycoproteins in *C. fetus*.

## Results

### Characterization of *Cff pgl* Cluster

**FIGURE 2:**
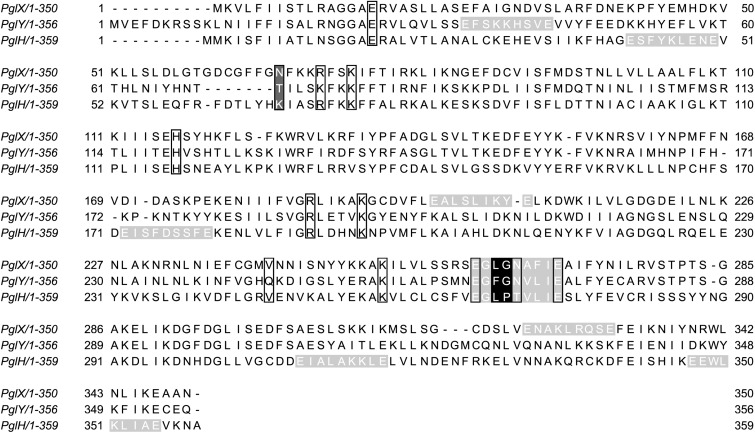
Sequence alignment of *C. fetus* (*Cf*) PglX, PglY, and *C. jejuni* (*Cj*) PglH. Black boxes indicate specific amino acids associated with activity in PglH (Troutman and Imperiali, 2009; Ramirez et al., 2018). Black and dark gray amino acids represent functional residues that show non-conserved substitutions in PglX and PglY. Light gray highlighted sequences indicate EX_7_E motifs commonly found in glycosyltransferases (Cid et al., 2000; Coutinho et al., 2003; Troutman and Imperiali, 2009). The catalytic EX_7_E motif of *Cj*-PglH is located at residues E266 to E274. *Cf*-PglX CFF8240_1386, *Cf*-PglY CFF8240_1385, and *Cj* DDV78_00080 sequences were analyzed by Jalview (Waterhouse et al., 2009).

The *C. fetus* (*Cf*) *pgl* cluster is syntenic with the *C. jejuni* (*Cj*) *pgl* gene cluster (Jervis et al., 2012; Nothaft et al., 2012) apart from lacking *pglI* and possessing two homologs of *pglH* (Figure 1). The similarities between the two loci are reflected in their N-glycan structures, with both sharing the same three reducing end sugars. In *C. jejuni, pglC*, *pglA*, and *pglJ* are responsible for the formation of this initial diNAcBac-GalNAc_2_ trisaccharide that is conserved across nearly all *Campylobacter* species (Jervis et al., 2012; Nothaft et al., 2012). Previously, *Cf* was annotated to possess two *pglH* homologs; however, compositional and structural analyses of the *Cf*-*pgl* pathway products showed that it does not contain the three GalNAc residues added by the *Cj*-*pglH* gene product (Nothaft et al., 2012). Since it is the non-reducing end of the *C. jejuni* and *C. fetus* N-glycans that varies in structure, the *pgl* genes in the “variable” glycosyltransferase (GTase) region upstream of *pglB* most likely differ in function. We therefore named the two *pglH* homologs, *pglX* and *pglY* (Figure 1). Interestingly, both proteins contain the catalytic EX_7_E motif previously annotated in PglH (Cid et al., 2000; Troutman and Imperiali, 2009; Figure 2). In addition to this catalytic EX_7_E, PglY and PglX contain one and two additional EX_7_E motifs, respectively. K68 of *Cj-*PglH, which is believed to be involved in lipid-linked oligosaccharide (LLO) association, is altered to N67 and T70 in PglX and PglY, respectively. In addition, the binding site of the *Cj*-PglH catalytic EX_7_E motif that involves L269 and P270 was found to be altered in PglX and PglY. Both enzymes possess a G instead of a P at position P270; however, only PglY possesses an F at position 267 that corresponds to L269 in *C. jejuni*. These minor changes in the amino acid residues may explain the differences in enzyme specificity and the formation of the shorter glycans when compared to *C. jejuni.*

### N-Glycan Analysis of *Cff*-*pgl* Mutants

To assess the functions of the “variable” GTases, we constructed mutants by insertion of a kanamycin resistance cassette (referred to as “kan”) into the respective gene loci. Both *pglX* (*pglX:kan*, further referred to as *pglX-*) and *pglJ (pglJ:kan*, further referred to as *pglJ-*) were constructed in the *Cff* strain ATCC 27374 (Supplementary Figure S1), however multiple attempts at generating mutants in *pglY* were unsuccessful.

**FIGURE 3:**
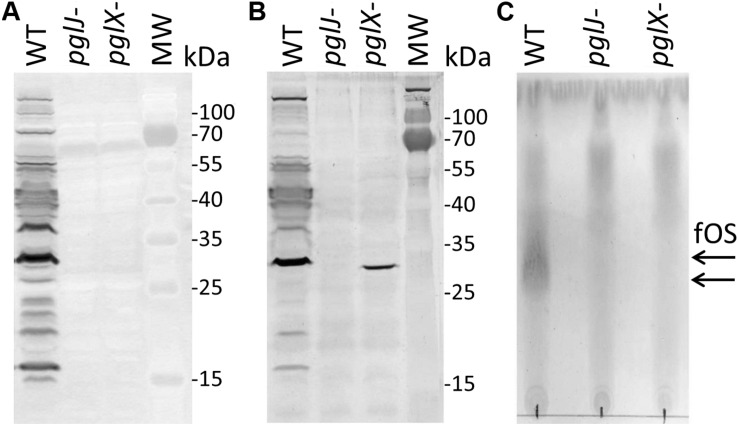
Product analysis of *Cf* WT, *pglX-* and *pglJ-* strains. **(A)** Western blot of whole-cell lysates with *Cff-*N-glycan-specific antiserum, and **(B)** wheat germ agglutinin reactivity of whole cell lysates of the WT, *pglJ*-, and *pglX-* strains. **(C)** Thin-layer chromatography (TLC)-free oligosaccharide (fOS) analysis of WT, *pglJ*-, and *pglX-* strains. Molecular weight (MW) markers for the western blots (in kDa) are indicated on the right; arrows indicate the migration of WT fOS on the TLC plate.

Insertion of the *kan* cassette in the *pglJ* and *pglX* genes was verified by PCR with oligonucleotides hybridizing outside of the recombination event (Supplementary Figure S1). When compared to the PCR product size obtained with chromosomal DNA isolated from *Cff*-WT, an increase in size by approximately 1.8 kb was observed when the *kan* cassette was present on the respective PCR product, clearly indicating insertion at the correct position within the *Cff* chromosome. To further investigate the effect of the mutations on N-glycan biosynthesis, western blot analysis of whole cell lysates probed with *Cff*-N-glycan specific serum was performed. Complete loss of serum reactivity in *pglX-* and *pglJ-* was observed when compared to the WT (Figure 3A and Supplementary Figure S6A). Lectin blotting with WGA confirmed those results, i.e., loss of reactivity in whole cell lysates of the *pglJ* mutant and strongly reduced reactivity (with only one signal present) in lysates of the *pglX* mutant (Figure 3B and Supplementary Figure S6B). Similarly, no free oligosaccharides (fOS) could be detected in the two *pgl* mutants when analyzed by thin layer chromatography (TLC) (Figure 3C). Here, two spots for the *Cff*-fOS variants could be seen when a fOS preparation of the WT was applied, confirming previous observations (Dwivedi et al., 2013), and these spots were absent in similar preparations from the *pglX-* and *pglJ-* strains.

**FIGURE 4:**
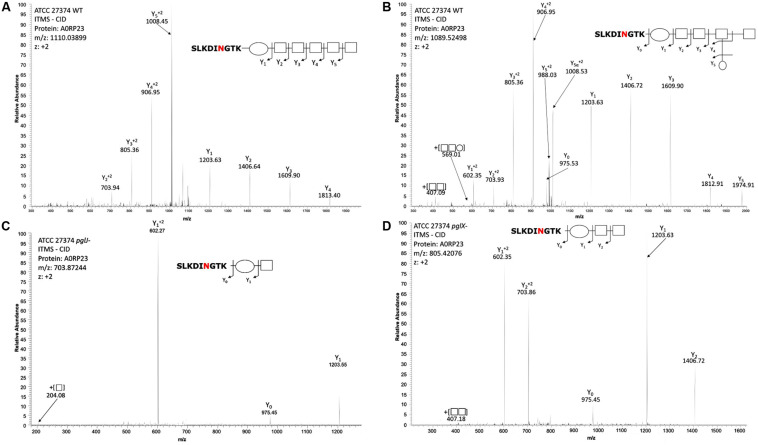
Mass-spectrometric analysis of *Cff* WT, *pglJ-*, and *pglX-* glycopeptides. Fragmentation of characteristic ions obtained by precursor ion scanning of digested *Cff* lysate samples using liquid chromatography-mass spectrometry. Red lettering indicates possible glycosylation site. Spectra of WT *Cff* have two glycans: **(A)** HexNAc_5_-diNAcBac and **(B)** HexNAc-[Hex]-HexNAc_3_-diNAcBac. **(C)** The peptide from the *pglJ-* mutant only shows the presence of a mass consistent with HexNAc-diNAcBac. **(D)** The peptide from the *pglX-* mutant indicates that it is modified with HexNAc-HexNac-diNAcBac.

To investigate the N-glycan in the two *Cff*-*pgl* mutants in more detail, proteomics analysis of *Cff-*WT and the *pgl* mutants was performed. As the disruption of *Cff*-*pgl* was predicted to truncate the N-linked glycan, we examined whole cell lysates to avoid potential biases in the detection of glycoforms that can result from glycopeptide enrichment (Alagesan et al., 2019). Consistent with our previous work (Nothaft et al., 2012), we observed both GlcNAc-α1-6-(GlcNAc-β1-3)-GlcNAc-α1-4-GalNAc-α1-4-GalNAc-α1-3-diNAcBac and GlcNAc-α1-6-(Glc-β1-3)-GlcNAc-α1-4-GalNAc-α1-4-GalNAc-α1-3-diNAcBac glycans on multiple protein substrates within the WT (Figures 4A,B), which were absent within *pglX- and pglJ-* (Supplementary MS Data 1). Consistent with our western and lectin blotting assays, multiple truncated N-linked glycans were observed within *pglX-* and *pglJ-* including diNAcBac-HexNAc_2_ glycans, diNAcBac-HexNAc glycans (Figures 4C,D) as well as diNAcBac alone. Within *pglX*-, the diNAcBac-HexNAc_2_ glycan was the predominant glycoform (Supplementary MS Data 1) and is consistent with the *Cff* N-glycan core structure, diNAcBac-GalNAc_2_ (Nothaft et al., 2012). In contrast, multiple glycoforms were identified in *pglJ-* including diNAcBac-HexNAc_2_-, diNAcBac-HexNAc, and diNAcBac glycans (Supplementary MS Data 1). Taken together these results confirm the involvement of PglJ in the formation of the conserved reducing end trisaccharide and that PglX functions in extension of the non-conserved N-linked glycan structure.

### Mutations in *pglX* and *pglJ* Have No Effect on Growth and the Expression of Downstream Genes but Reduces Motility

Growth curves were performed to investigate a potential influence of the *pgl* mutations. Although the *pgl* mutants reached a slightly higher optical density in the late logarithmic phase when compared to the WT, the final optical densities, as well as the growth rates in the early and mid-exponential phases, were similar among the three strains (Supplementary Figure S2). In addition, we did not observe a significant difference in *pgl* gene transcript levels after the insertion of the *kan* cassette in either *pglX* or *pglJ* (Supplementary Figure S3). This indicates that expression of the antibiotic cassette has no effect or that other transcriptional start sites in the *Cff*-*pgl* operon are compensating, as observed in the *C. jejuni pgl* operon (Szymanski et al., 1999; Dwivedi et al., personal communication). A downstream effect would influence expression of *pglB* but we see similar abundance of the PglB protein in WT when compared to either mutant (Supplementary Table S1). In addition, we still observe different forms of glycans on each mutant whereas in the absence of PglB we would not expect any glycans at all. However, we observed a significantly reduced swimming behavior in *pglX-* and *pglJ-* when compared to *Cff*-WT (Supplementary Figure S4) indicating that N-glycosylation either directly or indirectly affects motility.

### Characterization of PglJ and PglA in *E. coli*

**FIGURE 5:**
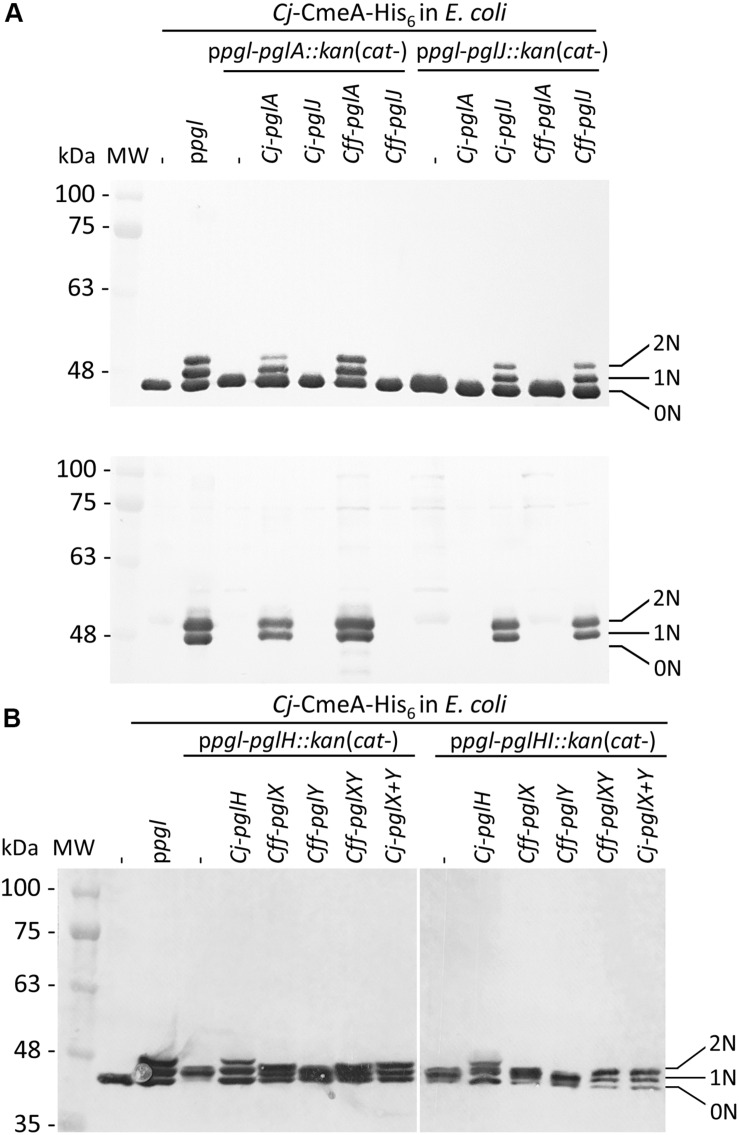
Functional analysis of *Cff*-Pgl pathway glycosyltransferases (GTases) in the heterologous *E. coli* glycosylation system. The GTase-activities of **(A)** PglA and PglJ were analyzed in western blots of *Cj-*CmeA-His_6_ with His_6_-tag antibodies (upper panel) and *Cj-*N-glycan specific (R1) antibodies (lower panel). **(B)** PglX and PglY activities were analyzed with His_6_-tag specific antibodies in western blots of CmeA-His_6_ used as the glycan acceptor to determine N-glycosylation activities. Whole cell extracts (5 μg) of *E. coli* CLM24 expressing the indicated gene/plasmid combinations are indicated above each lane. Non-, mono-, and di-glycosylated CmeA-His_6_ proteins are labeled as 0N, 1N, and 2N, respectively. Molecular weight markers (MW) in kDa are indicated on the left.

Since the N-glycan phenotype observed in the *pglJ* mutant was somewhat unexpected, the function of *Cff*-PglJ and *Cff-*PglA was further investigated by using a modified heterologous *E. coli Cj*/*Cff* hybrid glycosylation system (Wacker et al., 2002). Within this system, *Cff*-Pgl proteins are expressed in the presence of a mutant *Cj-pgl* operon (lacking select *Cj*-*pgl* genes). The glycans produced are then transferred to *Cj-*CmeA-His_6_ (N-glycosylation acceptor protein) via *Cj-*PglB. Western blotting of whole cell lysates of *E. coli* CLM24 prepared after co-expression of *Cj-*CmeA-His_6_ and *Cj-pglA* or *Cj-pglJ* in the presence of p*pgl* operon derivatives lacking either *pglA* or *pglJ* were probed with anti-His_6_ and anti-*Cj*-N-glycan antibodies (Figure 5A and Supplementary Figure S7A). The three *Cj-*Cme-His_6_-specific signals with anti-His (Figure 5A and Supplementary Figure S7A upper panel) and two N-glycans specific signals with the *Cj*-N-glycan specific R1 antiserum (Figure 5A and Supplementary Figure S7A lower panel) clearly identified the bands as non-(0N), mono-(1N), and di-(2N) glycosylated CmeA-His_6_. A similar *Cj-*CmeA-His_6_ pattern was produced in cells harboring the native *Cj*-*pgl* operon (from p*pgl*) and upon expression of *Cff-pglA* or *Cff-pglJ* (although with lower glycosylation efficiency) only when the *Cj*-homologous gene was knocked-out. In addition, no cross-complementation could be observed when *Cj* or *Cff*-*pglA* or *pglJ* were expressed in the presence of the p*pgl* plasmid lacking *pglJ* or *pglA*, respectively. These results confirm that *Cff*-PglA and *Cff*-PglJ fulfill the same functions as the homologous *Cj*-Pgl proteins, i.e., the addition of the second and third monosaccharide building blocks, respectively, to Und-diNAcBac, to form the diNAcBac-GalNAc_2_- trisaccharide. As expected, no *Cj-*CmeA-His_6_ glycosylation was observed in the absence of p*pgl* resulting in only non-glycosylated (0N) acceptor protein represented by a single band in the anti-His_6_ western blot and further confirmed by the absence of the N-glycan-specific signals in the anti-N-glycan (R1) blot (Figure 5A and Supplementary Figure S7A lower panel). Mass spectrometric analysis of isolated *Cj-*CmeA confirmed the modification of CmeA glycopeptides with the expected glycoforms supporting these western blot results (Supplementary Material and Supplementary MS Data 2). Here, the full length *Cj*-heptasaccharide was produced only when the *Cj*-*pgl* operon plasmids with mutations in *pglA* or *pglJ* were co-expressed with plasmids containing the corresponding *pglA* or *pglJ* from *Cj* or *Cff*.

### Characterization of *Cff-pglX* and *pglY* Using the *E. coli* Heterologous Glycosylation System

Since we could not obtain a mutant in *Cff-pglY* and therefore could not assign the functions of the two remaining GTases in the “variable” *pgl* region, we decided to analyze PglX and PglY using the heterologous *E. coli* glycosylation system (Wacker et al., 2002). In this case we employed the *Cj-pgl* operon lacking *pglH* that produces a trisaccharide (diNABacGalNAc_2_) identical to that found in *Cff*, potentially providing a substrate for PglX or PglY activity. In addition, we constructed and analyzed the complementation of a p*pgl-pglHI*:*kan* mutant plasmid (lacking *Cj-pglH* and *Cj-pglI*) to rule out the possibility of the *Cj*-PglI GTase adding or competing with the potential addition of a glucose residue to the N-glycan chain by either *Cff*-PglX or *Cff*-PglY. To do so, plasmid pCE111/28 derivatives expressing *Cj-pglH* (positive control), *Cff-pglX*, *Cff-pglY*, or *Cff-pglXY* served as complementation vectors. Western blots of whole cell lysates probed with anti-His_6_ antibodies were performed to investigate the *Cj-*CmeA-His_6_ glycosylation pattern in the underlying strains (Figure 5B and Supplementary Figure S7B). Expression of p*pgl* in combination with CmeA-His_6_ and CmeA-His_6_ alone served as positive and negative glycosylation controls, respectively. First, we demonstrated that expression of *Cj-pglH* in combination with the *pgl* operon lacking *pglH* resulted in a glycosylation pattern similar to the strain co-expressing CmeA-His_6_ and the *Cj*-WT *pgl* operon (on p*pgl*), i.e., production of non-(0N), mono-(1N), and di-(2N) glycosylated CmeA-His_6_, whereas in the absence the complementation plasmid, glycobands were migrating slightly faster due to the addition of only the trisaccharide N-glycan (missing the GalNAc_3_-Glc that is added by PglH and PglI in the full length *Cj-*heptasaccharide). Expression of *Cff-pglY* with p*pgl-pglH*:*kan* did not alter the migration behavior of the glycobands when compared to p*pgl-pglH*:*kan* alone, whereas transformation of *Cff*-*pglX* resulted in a slight mass increase compared to p*pgl-pglH*:*kan/Cff-pglY*, indicating that PglX, but not PglY, might be responsible for the addition of a sugar residue to the p*pgl-pglH*:*kan* glycan (Figure 5B). A slight increase in mass of the *Cj-*CmeA-His_6_ glycoprotein was also observed upon expression of *Cff-pglXY* (*pglXY* cloned as one PCR product), however a difference in the running behavior compared to p*pgl-pglH*:*kan*/*Cff-pglY* could not be resolved by SDS-PAGE and western blotting analysis alone (Figure 5B and Supplementary Figure S7B).

Similar results were obtained upon introduction of *Cj-pglH* and *Cff pglX*, *Cff-pglY* and *Cff-pglXY* into CLM24 expressing p*pgl-pglHI:kan* and *Cj-*CmeA-His_6_. Here, the glycobands in the *Cj-pglH* complements were expected to display a slightly faster running behavior when compared to the full length heptasaccharide due to the loss of the Glc residue; however, similar to the complementation analysis of the p*pglH:kan* strains, an obvious difference in the running behavior of the CmeA-His_6_ glycobands upon introduction of *Cff*-*pglX*, and *Cff*-*pglXY* could not be resolved (Figure 5B).

To further investigate the N-glycans produced upon expression of the different *Cj*-*pgl* operon mutants in combination with the *Cff-pglX* and *pglY* complementation plasmids, mass-spectrometric analyses of trypsinized CmeA was undertaken. While N-glycan structures observed upon complementation with the *Cj*-control (p*pgl-pglH* mutant expressing *Cj-pglH*) resulted in the formation of the expected full length *Cj*-N-glycan, only one plasmid combination, the expression of *Cff*-*pglXY* in the p*pgl-pglH* mutant background resulted in the formation of a structure that was similar in composition and sequence to the minor form of the native *Cff*-N-glycan, diNAcBac-HexNAc_4_-Hex (Supplementary Material and Supplementary MS Data 2).

### Mutations in *pglX* and *pglJ* Have an Impact on Multiple Cellular Functions in *Cff*

**FIGURE 6:**
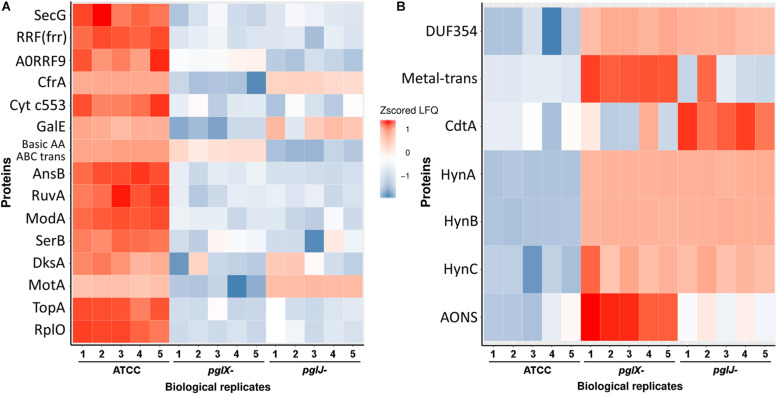
Label-free quantification of proteins in *Cff*-*pglJ-* and *pglX-* strains compared to WT. **(A,B)** Heat maps of specific proteins with statistically significant decreases **(A)** or increases **(B)** in *pglJ-* and *pglX-* mutants compared to WT (labeled ATCC). Values are gray where MS did not identify fragments. This data represents samples from five biological replicates (B1–B5). The complete dataset is included in Supplementary Table S1.

To further understand the role of N-glycosylation in *Cff*, label-free quantitative (LFQ) proteomics analysis of whole cell lysates of *Cff-*WT, and the *pglX-* and *pglJ-* was done. Across five biological replicates of each sample type (Supplementary Figure S5), 914 proteins were identified representing ∼77% of the *Cff* ATCC 27374 predicted proteome of 1,190 proteins (Supplementary Table S1). Quantitative proteome analyses revealed more than 100 proteins with significantly different abundance across various biological groups as shown in heat maps of the most prominent differences in abundance comparing WT to *pglX-* and *pglJ-* strains (Figures 6A,B). These results indicate that mutating glycosyltransferases involved in assembly of the N-linked glycan has a significant effect on abundance of numerous cellular proteins.

### Expression of the H_2_-Uptake Hydrogenase Complex HynABC Is Significantly Induced in Both *pglJ* and *pglX* N-Glycosylation Mutants

**FIGURE 7:**
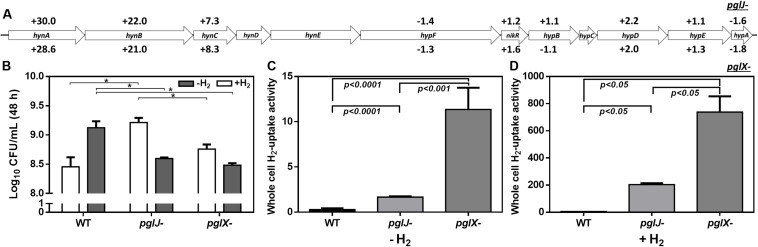
Elevated hydrogenase levels in *Cff*-*pgl* mutants correlate with increased H_2_ uptake. **(A)** Gene arrangements of *hyn* and *hyp* clusters in *Cff* ATCC 27374 with LFQ proteomic protein abundance in *pgl* mutants vs WT are shown. Protein fold changes of *pglJ-* and *pglX-* with respect to WT are indicated above and below each gene respectively. Genes with no values associated had no coverage in our proteomics data analyses. Only HynABC showed significant protein level increases when compared to WT. **(B)** Effect of H_2_ on microaerobic growth of *Cff* WT, *pglJ*-, and *pglX*- strains. The data set is derived from three biological replicates (with two technical replicates each) of cells grown under the indicated condition. Number of cells obtained after 48 h of growth is represented by Log_10_ colony forming units per mL (CFU/mL). The error bars represent the standard error within each group. **(C,D)** Whole cell H_2_-uptake of *Cff* WT, *pglX-*, and *pglJ*- strains. *Cff* was grown on BHI agar in microaerophilic conditions for 24 h at 37°C in 10% H_2_ (+H_2_) or absence of hydrogen (–H_2_). Whole cell H_2_-uptake activity is expressed as nanomoles of H_2_ used per min per 10^9^ cells. Results in **(C)** represent the mean ± SD of four independent assays; results in **(D)** represent the mean ± SD of two independent assays. *p*-values as analyzed by a two-tailed t-test are either indicated by an asterisk (*p-value ≤ 0.05 in **B**) or are directly included in the figure (for **C,D**).

Among the proteins with increased abundance in both the *pglJ* and *pglX* mutants (compared to WT) were the three subunits (HynABC) of a putative nickel-iron (NiFe) H_2_-uptake hydrogenase complex (Benoit et al., 2020). In both *pgl* mutants, the expression levels of all three hydrogenase subunits, HynA, HynB, and HynC, were significantly higher compared to the WT (means of 29.3-fold, 21.5-fold, and 7.8-fold, respectively) (Figure 7A and Supplementary Table S1). This complex, found in a number of bacterial pathogens, enables the microbes to use the electron donor H_2_ as an energy source, thus providing an alternative respiratory pathway that is important for *in vivo* survival (Olson and Maier, 2002; Benoit and Maier, 2018). HynABC-associated proteins, such as hydrogenase accessory/maturation proteins (e.g., HypABCDEF) or the nickel specific transcriptional regulator (NikR) also showed moderate increases in protein levels in both mutants compared to WT (Figure 7A).

Since H_2_ increases growth of various ε-Proteobacteria species, including *Helicobacter pylori* and *Campylobacter concisus* (Kuhns et al., 2016; Benoit and Maier, 2018), we determined whether higher hydrogenase expression in the *C. fetus* N-glycosylation mutants correlates with elevated H_2_-supported microaerobic growth. To do so, the cell yield (CFU/mL) of the WT and the *pglJ* and *pglX* mutants was assessed after 48 h of growth under microaerobic conditions in the presence or absence of 20% H_2_ (Figure 7B). We only determined the end point of growth due to the extended lag phase of *Cff* cultures grown under these conditions. With no added H_2_, *Cff* WT had a significantly higher growth yield compared to both mutants. However, in H_2_-enriched conditions, WT cells showed growth levels comparable to both *pgl* mutants. Although the addition of H_2_ was originally predicted to be beneficial for WT growth, we observed decreased growth in H_2_ for other *Cf* strains, *Cft* 03-427 and *Cff* 82-40 (data not shown). In contrast, we observed a significant increase in *pglJ-* growth compared to the other strains in the presence of H_2_. A slight increase in *pglX-* growth was also observed in the presence of H_2_, but it was not significant compared to WT. These results indicate that the *pgl* mutants have increased growth yield in H_2_ opposed to WT where H_2_ is deleterious.

H_2_-uptake in whole cells was examined to determine whether increased HynABC levels in the mutants correlate with increased H_2_-uptake activity. Cells were grown in microaerobic conditions in the presence or absence of supplemental H_2_ and hydrogenase activity was determined using a previously described amperometric method (Maier et al., 1996). The hydrogenase activity (expressed in nmoles of H_2_ oxidized per min per 10^9^ cells) was 0.3 ± 0.07, 1.7 ± 0.04, and 11.4 ± 1.2 for WT *pglJ*-, and *pglX*, respectively, when cells where grown under microaerobic conditions in the absence of supplemental H_2_ (Figure 7C). This represented almost a 6-fold (for *pglJ-*) to 39-fold (for *pglX-*) increase in activity compared to WT. When cells were grown in the presence of 10% H_2_, we observed a 122- and 65-fold increase in hydrogenase activity in *pglJ-* and *pglX-*, respectively, and a 20-fold increase in WT (Figure 7D). The remarkable H_2_-uptake levels measured for *pglJ-* (204 ± 7 nmoles H_2_/min/10^9^ cells) and *pglX-* (738 ± 82 nmoles H_2_/min/10^9^ cells) mutants grown with H_2_ were the highest recorded values to date for a bacterial pathogen. Taken together, these results indicate an inverse correlation between N-glycosylation and H_2_ usage (i.e., hydrogenase synthesis and activity) in *Cff.*

### N-Glycosylation Influences Transition Metal Profiles

Proteomics data indicate that multiple proteins associated with transition metals were significantly altered in both *pgl* mutants. These include ModA, involved in molybdenum transport (−49.9-fold in *pglX*- and −71.8-fold in *pglJ-*); the ZinT/AdcA family protein involved in zinc binding (−12.2-fold in *pglX*- and −6.5-fold in *pglJ*-); CfrA, a ferric receptor (−118.4-fold and −3.3-fold); and an iron ABC transporter (−5.1-fold and −7.6-fold) (Supplementary Table S1). Also, a copper/cadmium-translocating P-type ATPase protein was found to be significantly increased (51.9-fold) in *pglX-*; however, the increase was not significant (1.4-fold) in *pglJ-*.

**FIGURE 8:**
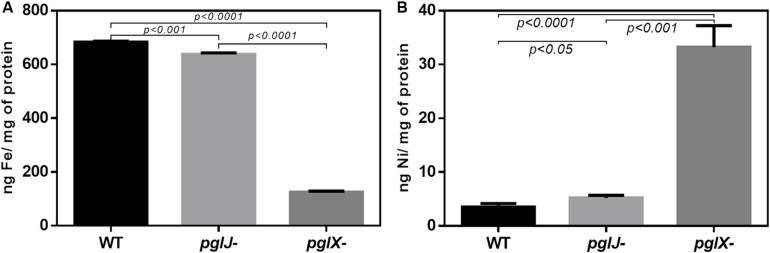
Cellular iron and nickel content of *Cff* WT, *pglX-*, and *pglJ*- strains. Atomic absorption spectroscopy (AAS) was employed for the detection of **(A)** iron (Fe) and **(B)** nickel (Ni) in lysed cells of the indicated strain. Data are presented as the mean of at least three replicates, error bars depict the standard deviations. Statistically significant differences determined by a two-tailed *t*-test are indicated.

The increased levels, especially of the HynABC (Ni-Fe) hydrogenase observed in both *pgl* mutants, led us to further investigate nickel and iron levels in these strains. Using atomic absorption spectrometry (AAS) of lysed cells, we found that iron levels were dramatically decreased in *pglX-* (125.1 ng/mg protein), that is almost sixfold lower when compared to WT (683.2 ng/mg protein) whereas iron levels in *pglJ-* were modestly, but statistically significantly, decreased (Figure 8A). In addition, the *pgl* mutants had significantly higher levels of cellular nickel content compared to WT (Figure 8B); *pglX-* had a nickel content of 33.2 ng/mg protein that was almost 10-times higher than in the WT (3.5 ng/mg protein). Although still significantly higher when compared to the WT, *pglJ-* (5.2 ng/mg protein) had almost sixfold less nickel than *pglX-*. These results indicate that N-glycosylation might be vital in regulation of nickel homeostasis, iron, or both.

### Antibiotic Sensitivity and Increased Membrane Efflux

**FIGURE 9:**
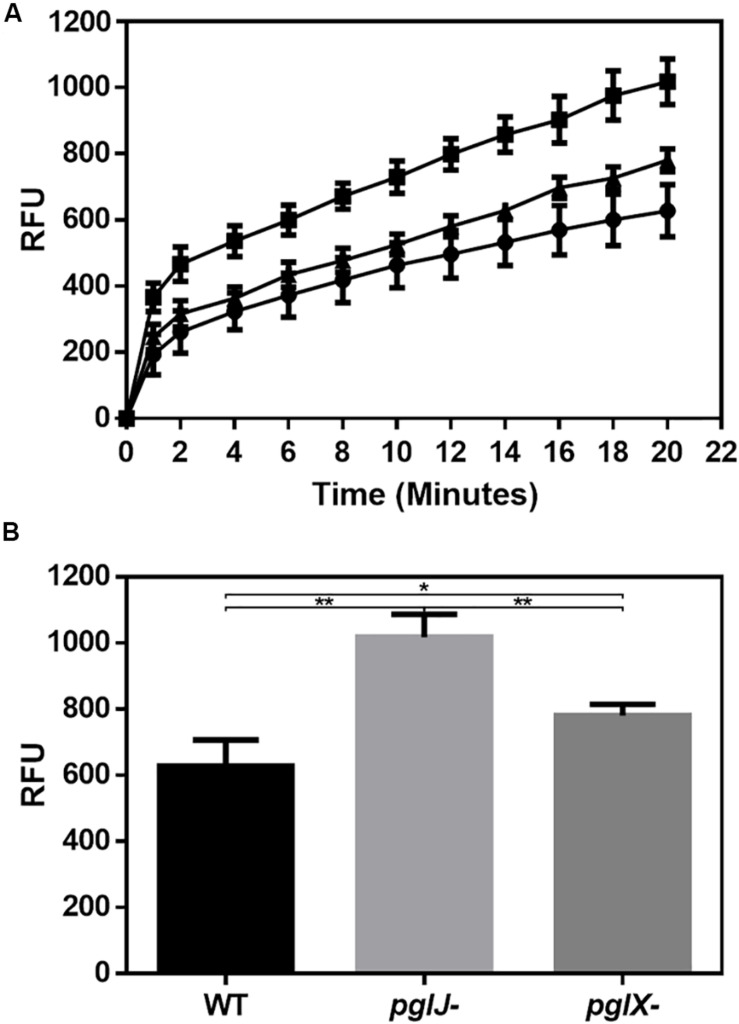
Mutations in N-linked protein glycosylation decreased efflux in *pglX-* and *pglJ*- strains. **(A)** Accumulation of ethidium bromide in cultures of *Cff* WT (circle), *pglJ-* (square), and *pglX-* (triangle) over a time frame of 20 min. Relative fluorescent units (RFU) are indicated on the *y*-axis. **(B)** Bar graph depicting the relative fluorescence at *t* = 20 min. The error bars represent the standard error for each data set consisting of four biological replicates with three technical replicates each. ** *p*-value ≤ 0.001, * *p*-value ≤ 0.05 as determined by a two-tailed *t-*test.

Our previous study showed that *C. jejuni* N-glycosylation was required for optimal activity of the CmeABC multidrug efflux pump necessary for antibiotic resistance (Dubb et al., 2020). In *Cff*, albeit not statistically significant, we found increased levels of CmeA, CmeB, and CmeC in *pglX-* and *pglJ-* (mean of both mutants: 2.0-fold CmeA, 1.9-fold CmeB, and 2.0-fold CmeC). Therefore, we examined the antibiotic sensitivity profiles of both *Cff* N-glycosylation mutants. As shown in Supplementary Table S2, the *pglX-* and *pglJ-* strains showed twofold increase in sensitivities to chloramphenicol, gentamicin, azithromycin, and sulfisoxazole, and a fourfold increase in sensitivity to ampicillin suggesting a correlation between N-glycosylation and antibiotic resistance in *C. fetus*, similar to previously observed in *C. jejuni* (Abouelhadid et al., 2019; Dubb et al., 2020). To explore this further, we used ethidium bromide (EtBr), a DNA intercalating agent, to quantitatively assess efflux pump activity over time. Both *pglJ* and *pglX* mutant strains showed significantly higher levels of EtBr accumulation compared to WT (Figure 9); however, accumulation was less pronounced in the *pglX* mutant. Taken together, these results suggest that N-glycosylation in *C. fetus* may be important for efflux pump activity and antibiotic sensitivity.

## Discussion

N-glycosylation is a conserved mechanism in all domains of life. The prototypical *pgl* N-glycosylation system, originally characterized in *C. jejuni* (*Cj*), has orthologs in many δ- and ε-Proteobacteria (Nakagawa et al., 2007; Jervis et al., 2010; Ielmini and Feldman, 2011; Nothaft et al., 2012; Mills et al., 2016). Non-thermotolerant *Campylobacter* species, like *C. fetus* (*Cf*), including *C. fetus fetus* (*Cff*) and *C. fetus venerealis* (*Cfv*), have been found to produce more than one N-glycan, unlike *Cj* which expresses one distinct heptasaccharide (Scott et al., 2011; Nothaft et al., 2012; Cain et al., 2019).

In our study, we generated mutants in PglX and PglJ in *Cff* strain ATCC 27374. Glycopeptides from the *pglX-* mutant showed fragmentation patterns consistent with the conserved diNAcBac-GalNAc_2_ (Nothaft et al., 2012), suggesting that PglX is responsible for the addition of the first GlcNAc residue to the *Cf* N-glycan structure (Figure 1). The loss of *Cf* N-glycan-specific serum reactivity and WGA lectin binding to lysates from the *pglX-* strain support this claim. Proteomics of the *pglJ-* strain resulted in a mixture of glycopeptides, primarily consisting of diNAcBac and a few fragments of diNAcBac-HexNAc, typically more characteristic of a *Cj* PglA mutant. To investigate this further, we used an *E. coli* expression system followed by MS-analyses and were able to show that the *Cff*-PglJ and *Cff-*PglA had similar transferase activities onto recombinantly expressed *Cj*-CmeA as the *Cj* homologs. However, we did not see reactivity with WGA or the *Cff-*N-glycan specific antiserum in the *Cff-pgl* mutants, except for a single band for *pglX-* in the WGA blot. These results suggest possible WGA interaction with another glycan, such as LPS, however the reason behind the absence of specific binding in the *pglJ* mutant strain has yet to be explained. Nevertheless our results suggest that the formation of the diNAcBac-GalNAc_2_ trisaccharide is conserved between *Cj* and *Cff* and that the observed differences in antigenicity (Jervis et al., 2010; Nothaft et al., 2012) stem from the non-reducing end.

Expression of *Cff*-PglX in *E. coli* showed a CmeA mass shift and with transfer of an additional sugar, consistent with transfer of an additional sugar and with our MS-analysis of glycopeptides from the native host (i.e., diNAcBac-GalNAc_2_-GlcNAc). Since we were unable to generate a pglY mutant in *Cff*, we also used the *E. coli* system to investigate *Cff*-PglY activity and detected a major glycoform with an additional sugar only when both PglX and PglY were co-expressed, suggesting that PglY’s activity is dependent on the initial modification by PglX. Based on our MS results, an N-linked glycan with a composition resembling the minor *Cff* N-glycan [i.e., GlcNAc-α1-6-(Glc-β1-3)-GlcNAc-α1-4-GalNAc-α1-4-GalNAc-α1-3-diNAcBac] was also observed in *E. coli* p*pgl-pglH*:*kan* expressing *pglX* in combination with *pglY*, although the addition of the Glc residue by *Cj*-PglI could not be ruled out since we observed less peptides containing the minor *Cff*-N-glycan in the p*pgl-pglHI* background, and also observed Glc addition in a p*pgl*-*pglHI*:*kan* mutant demonstrating that an *E. coli* enzyme could be contributing this residue. Thus, the GlcTF reaction requires further investigation either in *Cff* or *in vitro*. No N-glycan that resembles the major form of the *Cff* N-glycan could be detected with any *Cj*-*pgl* mutant/*Cff*-*pgl* gene combinations. Nevertheless, these data suggest that *pglX* and *pglY* can mediate the construction of a partial *C. fetus* N-linked glycan using the *C. jejuni* diNAcBac-GalNAc_2_ trisaccharide as a substrate. *C. jejuni* PglB does not have strict substrate specificity and can transfer full-length and truncated N-glycans and diverse O-antigen structures in *E. coli* and to a lesser extent, in the native host (Feldman et al., 2005; Linton et al., 2005). Therefore, we did not expect preferential transfer of certain *Cj*-*Cff* hybrid N-glycans to CmeA. However, since we only generated one potential variant of the *Cff*-N-glycan, this suggests that the GTase involved in the formation of the second *Cff*-N-glycan structure is either not fully functional in *E. coli* or is not part of the *pgl* locus, similar to the lack of *pgl* gene clustering in *Helicobacter* species and *D. desulfuricans* (Jervis et al., 2010; Nothaft and Szymanski, 2010).

To better understand the role of N-glycosylation in *Cff*, we utilized LFQ proteomics comparing *Cff* ATCC 27374 with two isogenic *pgl* mutants, *pglX-* and *pglJ-*. Through this approach, we were able to detect almost 77% of the (genome-inferred) total proteins. Analysis of proteins that were significantly up- or down-regulated indicated that more than 100 proteins were altered in the *Cff pgl* mutants in comparison to WT. It is worth noting that differences between *pgl* mutants may be due to differences in glycan length (diNAcBac-GalNAc in *pglJ*- and diNAcBac-GalNAc_2_ in *pglX*-) or differential feedback regulation in these two backgrounds. Although N-glycosylation was not completely eliminated, we observed a decrease in NapB (−6.7-fold in *pglX*- and −8.4-fold in *pglJ*-, Supplementary Table S1), similar to that previously seen in a *Cj*-*pglB* mutant (Cain et al., 2019). In *C. jejuni*, the nitrate reductase NapAB has been shown to be a two-subunit enzyme, with both subunits being N-glycosylated (Scott et al., 2011; Mintmier et al., 2018; Abouelhadid et al., 2019; Cain et al., 2019). In contrast, in *Cff* ATCC 27374, NapA lacks an N-glycosylation sequon, while at the same time NapB has two potential sequons. This may explain why we only observed a decrease in NapB (see above), while the difference in NapA protein levels was not significant (1.1-fold in both *pgl* mutants).

No effect on growth or on the expression of downstream genes was observed, but the *pgl* mutants were impaired in motility. Similarly, loss of *pglB* (and therefore complete loss of N-glycosylation) in *C. jejuni* JHH1 and *C. jejuni* 11168 also resulted in decreased motility when compared to WT cells (Scott et al., 2012; Cain et al., 2019). In addition, Cain et al. (2019) demonstrated that levels of specific proteins required for motility were expressed at significantly lower levels in the *C. jejuni* 11168 *pglB* mutant; among them MotA, MotB, and FlgP. We also observed lower levels of MotA and MotB (significantly lower in *pglX-*; 94- and 8.6-fold, respectively), but not in *pglJ-* (Supplementary Table S1); FliG [significantly lower in *pglX-* (5.7-fold) and *pglJ-* (4.7-fold) (Supplementary Table S1)], as well as the *Cj*-FlaA homolog flagellin protein [significantly lower in *pglX-* (9.7-fold) and *pglJ-* (7.1-fold) (Supplementary Table S1)]. This could imply that motility may be correlated with N-glycosylation changes in some Campylobacters. However, *pglB, pglE*, *pglF*, and *pglH* mutants in *C. jejuni* 81–178 were described to display WT levels of motility (Szymanski et al., 1999; Hendrixson and DiRita, 2004), therefore it seems that this regulatory network varies even among strains.

We did not observe a reduction in CmeABC in either the *Cff pglX* or *pglY* mutant. In contrast, we observed a slight, but not statistically significant, increase in these efflux proteins in both mutants. Despite that discrepancy, our *pgl* mutants still displayed decreased EtBr efflux activity compared to WT when cells were grown under the same conditions that were used to prepare whole cell lysates for proteomic analysis. This suggests that *Cf* N-glycosylation directly influences the activity of the efflux pump, an effect that has previously been described for *C. jejuni* (Abouelhadid et al., 2019; Dubb et al., 2020). However, the increased sensitivity to various classes of antibiotics observed in both *Cff*-*pgl* mutants is most likely indirect since not all of those antibiotics are substrates for the efflux pump in other *Campylobacter* species; however, variations in CmeABC substrate specificities have been observed even between strains (Lin et al., 2002; Akiba et al., 2006; Guo et al., 2010). One might speculate that membrane permeability increases due to lower abundance of certain periplasmic and/or membrane proteins or that loss of periplasmic fOS could result in a higher influx of those antibiotics and therefore lead to the observed decrease in MICs. It is worth noting that the observed effects were less pronounced in *pglX*- compared to *pglJ-*. This could be due to the fact that glycoproteins contain a longer N-glycan chain in *pglX-* compared to *pglJ*. Together these results indicate that N-glycosylation in *Cf* plays a role in efflux, although the mechanism is currently unknown.

Our proteomics data indicate that all three components (HynABC) of the (NiFe)-containing H_2_-uptake hydrogenase were significantly upregulated in both *pgl* mutants, suggesting that protein glycosylation plays a role in H_2_ utilization. Based on homology with hydrogenase complexes found in related ε-Proteobacteria, such as *H. pylori*, *C. jejuni*, and *C. concisus* (Olson and Maier, 2002; Weerakoon et al., 2009; Benoit and Maier, 2018), the *Cff* HynABC complex is likely to be involved in H_2_ oxidation. Consistent with the proteomics data, higher H_2_-mediated growth rates were observed in both *pgl* mutants compared to WT, with the highest growth rate seen in the *pglJ-* strain grown under H_2_ rich conditions. Surprisingly, H_2_-enriched conditions seemed to have a deleterious effect on WT growth. Nevertheless, we infer from these results that the improved growth observed in the mutants could be due to enhanced utilization of H_2_ from the drastically increased expression levels of the HynABC complex. In correlation with higher HynABC protein levels, H_2_-uptake activities were higher in both *pglJ-* and *pglX* mutants compared to WT in the absence and in the presence of H_2_. The increased (NiFe) hydrogenase synthesis (and activity) observed in the mutants might be linked to changes in metal homeostasis, particularly that pertaining to Fe and Ni. Studies conducted in the related organism *H. pylori* can provide insight into the respective roles of Fe and Ni with respect to transcriptional regulation of hydrogenase genes, through Fur and NikR regulators, respectively. For instance, *H. pylori* apo-Fur has been shown to repress *hynABC* (Ernst et al., 2005). Furthermore, addition of Ni to the medium leads to decreased *hynABC* expression; however, this repression was not observed in a *nikR* mutant background (Ernst et al., 2005) suggesting that either Ni-bound NikR represses or apo-NikR activates hydrogenase expression in *H. pylori*; in addition Ni-NikR has been shown to repress *fur* (Dosanjh et al., 2009). Taken together, these sets of results suggest the possible following mechanism in *Cff*: if Ni-bound NikR represses *fur* and (apo-) Fur represses *hynABC*, then elevated Ni levels (as observed in both *pgl* mutants) would be expected to de-repress Fur-controlled *hynABC.* The final outcome would be increased HynABC levels and increased hydrogenase activity, and indeed protein activities correlated well in cells and whole cell lysates grown under the same conditions. Obviously, the mechanism at play in *Cff* has yet to be elucidated. Nevertheless, taken together, our results indicate a clear link between N-glycosylation (or the lack thereof) and (NiFe) HynABC hydrogenase expression and/or enzymatic activity.

It is worth noting that *Cff* contains two additional hydrogenase complexes: a (FeFe) hydrogenase (HydA), hypothesized to be a H_2_-uptake type, and a (NiFe) H_2_-evolving complex (HycBCDEFG) predicted to be part of a formate hydrogen lyase (FHL) complex that links formate oxidation to hydrogen production (Benoit et al., 2020). Based on our proteomic study, neither HydA nor HycBCDEFG hydrogenase subunits were found to be expressed at different levels between WT and the N-glycosylation mutants.

The increase in (NiFe) HynABC and decrease in certain metal-related proteins prompted us to quantify Ni and Fe levels. In both *pgl* mutants we saw a significant decrease in iron; however, the decrease in iron for *pglX-* was fivefold lower than *pglJ*- and sixfold lower than WT. This may be because *pglX-* has a 118.4-fold decrease, and only 3.3-fold decrease in *pglJ*-, in the CfrA ferric enterobactin receptor present in *Cj*, which is responsible for high-affinity iron acquisition (Miller et al., 2009).

Although nickel is essential for both nickel containing hydrogenases in *Cf*, it is also toxic in excessive amounts, potentially causing oxidative stress and perturbing enzyme activities (Macomber and Hausinger, 2011). One mechanism of modulating nickel levels that was previously identified in *E. coli* is the nickel defense system (RcnA), which utilizes a proton gradient to translocate nickel to the periplasm where it can either be bound by sequestering proteins or effluxed from the cell (Macomber and Hausinger, 2011). We observed a 50-fold increase in a metal P-type ATPase in *pglX-* (A0RQS6), annotated as copper/cadmium-translocating P-type ATPase with similarly predicted activities. These metal P-type ATPase translocators are involved in detoxification of metals by transporting metals across the inner membrane (Ma et al., 2009). It is possible that this P-type metal translocator may be deficient at translocating; however, there is no clear link to N-glycosylation. These data are consistent with the cellular nickel levels of the *pglX*- strain, which were 6-times higher than the *pglJ-* strain. These increased nickel levels may be responsible for the higher hydrogenase activity levels measured in *pglX-* compared to the other two strains, while nickel toxicity could explain the decreased growth in H_2_ growth assays. Our data indicate that N-glycosylation regulates (NiFe)-hydrogenases HynABC, correlating with cellular nickel levels. Taken together, this suggests a possible link between our findings; however, their specific interaction with N-glycosylation is still unknown.

Our research connects N-glycosylation to HynABC hydrogenase regulation and nickel/iron homeostasis, two cellular processes which have been associated with pathogenicity in other bacteria (Palyada et al., 2004; Maier and Benoit, 2019; Benoit et al., 2020). The presented results deepen our understanding of the role of N-glycosylation in *C. fetus* cell physiology. In addition, the *Cf-*N-glycosylation system provides glycan diversity through PglX and PglY, which may further impact the biology of the microbe and warrants further investigation.

## Materials and Methods

### Bacterial Strains, Plasmids, Oligonucleotides and Growth Conditions

Oligonucleotides used in this study are listed in Supplementary Table S3. Bacterial strains and plasmids are listed in Supplementary Table S4. *C. fetus* was grown using Brain-Heart Infusion (BHI) medium (BHI-Hardy Diagnostics) and Columbia agar (CBA-Hardy Diagnostics) with 5% defibrinated horse blood (Hemostat, Dixon, CA, United States) under microaerobic conditions (10% CO_2_, 5% O_2_, 85% N_2_) at 37°C. *E. coli* was grown on 2xYT at 37°C or as indicated. If required, antibiotics were added to the following working concentrations: 100 μg/mL ampicillin, 25 μg/mL chloramphenicol, 50 μg/mL kanamycin, and 100 μg/mL spectinomycin.

### Preparation of Whole Cell Lysates and Western Blotting

Whole cell lysates of bacterial cells were prepared as described previously (Liu et al., 2006). Protein concentrations were determined using either the NanoVue Plus Spectrophotometer (GE) at A_280_ or by the BioRad DC Bradford assay kit with bovine serum albumin as a protein standard. Samples were either analyzed immediately or were frozen at −20°C until further use. Western blot analyzes was carried out as described (Nothaft et al., 2010) with anti-His (1:2000) (Rockland), anti-*Cff*-N-glycan (1:5000) (Nothaft et al., 2012), anti-*Cj-*N-glycan (R1, 1:7500) (Nothaft et al., 2012) or anti-CmeA (1:5000) (Wacker et al., 2002) as the primary, and anti-rabbit IgG (1:2000) (Santa Cruz Biotechnology) as the secondary antibody or with alkaline phosphatase labeled wheat germ agglutinin (WGA, 1:500) (EY Labs). Antibody and WGA-lectin reactive bands were visualized directly on the membrane with nitro-blue tetrazolium chloride (NBT) and 5-bromo-4-chloro-3′-indolyphosphate p-toluidine salt (BCIP) alkaline phosphatase substrate solution (Roche) according to the protocol of the manufacturer.

### Free Oligosaccharides Preparation and Analysis

Free oligosaccharides were obtained by ethanol extraction as described previously (Dwivedi et al., 2013) from 1 g of wet cell pellets. Free oligosaccharides (fOS) preparations were further purified using porous graphite carbon (PGC) columns as described (Liu et al., 2006). After elution and lyophilization fOS were dissolved in 100 μl of milliQ water and either stored at −20°C or directly analyzed by TLC as described (Dwivedi et al., 2013).

### Generation of *Cff pgl* Gene Mutant Constructs

First, a PCR product containing *Cff*-*pglKXYJ* (4868 nt) was generated with oligo CS469 and CS470 using chromosomal DNA from *Cff* as a template and inserted into the *Eco*RV site of plasmid pPCR-Script Amp SK(+). After transforming *E. coli* DH5α, plasmid-containing cells were isolated on plates supplemented with Amp and X-gal (40 μg/ml) and plasmids isolated from white colonies were analyzed by restriction digestion. One positive candidate (pPCR-Script-*Cffpgl*) that had the PCR product with the *Cff pgl* genes inserted in opposite direction to the *lacZ* gene was processed further. Next, plasmid pPCR-Script-*Cffpgl* was digested with either *Eco*RV (1 site within *pglX*), *Acc*I (1 site within *pglY*), or *Spe*I (1 site within *pglJ*). The linearized plasmid backbones were isolated and in the case of the *Acc*I and *Spe*I digests, T4 DNA polymerase was used to generate blunt ends before the DNA fragments were purified by agarose gel extraction. The kanamycin (*kan*) resistance cassette obtained and isolated after *Sma*I digestion of plasmid pMW2 was ligated with each vector backbone preparation. Amp and Kan resistant colonies obtained after ligation and transformation were screened and verified by restriction analyzes. One positive clone in which the *kan* cassette is transcribed in the same orientation as the corresponding reading frame (*pgl* gene) was used to generate the gene-specific insertions by double homologous recombination into the chromosome of *Cff*.

### Transformation and Insertion Mutagenesis of *Cff*

Natural transformation (on a BHI agar surface) (Wang and Taylor, 1990) and electroporation (Baillon et al., 1999) protocols were employed to introduce *Cff*-*pgl* gene:*kan* plasmid DNA for double homologous integration of the *kan* cassette into *Cff*. To do so, the corresponding suicide plasmids (pPCR-Script-*CffpglX:kan*, pPCR-Script-*CffpglY:kan*, and pPCR-Script-*CffpglJ:kan*) were isolated from either *E. coli* DH5α or *E. coli* JM110. The latter strain was used to generate non-methylated DNA to circumvent the *Campylobacter* restriction modification system. Transformants were selected on BHI plates for kanamycin resistance and individual colonies were isolated, streaked on fresh agar plates, and used to isolate chromosomal DNA. Candidate colonies were analyzed and verified by PCR with oligonucleotides hybridizing outside of the recombination event (Supplementary Figure S1) to confirm integration of the *kan* cassette at the correct position on the chromosome. One positive candidate (for *pglX*- and *pglJ*-) was used for further phenotypical analyzes, whereas (even after multiple attempts) no positive candidate could be obtained for the integration of the *kan* cassette into the *Cff*-*pglY* gene locus.

### Growth Curves and Motility Assays

Growth comparison was performed in BHI broth and growth curves were recorded as described (Dubb et al., 2020). Motility assays were carried out as outlined previously (Golden and Acheson, 2002) with slight modifications. Briefly, *Cff-*WT and *pgl* mutant strains were grown for 18 h on BHI agar. Cells were harvested from the plates with 2 ml of BHI broth and cell suspensions were diluted to an OD_600_ of 0.05. Then, 1 μl of each cell suspension was spotted onto a BHI 0.3% agar plate and after 24 h of incubation, images were taken and the diameter of the motility zone was measured horizontally and vertically.

### Reverse Transcriptase PCR

Reverse transcriptase (RT) PCR was performed according to Muraoka and Zhang (2011) with RNA extracted from cells grown on BHI agar for 18 h using the RNeasy Kit following the instructions of the manufacturer (Qiagen). PCR conditions after the RT-step were identical for each primer pair and were carried out as follows: 35 cycles with 30 s, 95°C; 30 s, 52°C and 20 s, 72°C followed by a 72°C finalizing step for 3 min. Samples were stored at 4°C before 15 μl of each 50 μl reaction were analyzed by 0.8% agarose gel electrophoresis.

### Pgl Gene Expressing Plasmids

Gene-specific oligonucleotides (Supplementary Table S3) were used to amplify *Cj*-*pglH, Cj*-*pglA*, *Cj*-*pglJ*, *Cff*-*pglA*, *Cff*-*pglJ*, *Cff*-*pglX*, and *Cff*-*pglY* as well as *Cff*-*pglXY* for expression in *E. coli*. To do so, PCR products obtained with specific template DNA (plasmid p*pgl* for the *C. jejuni pgl* genes or chromosomal DNA from *Cff*) were purified, treated with restriction enzymes (see Supplementary Table S3), and inserted into plasmid pCE111/28 digested with the same enzymes. To generate the *Cff*-*pglXY* expression plasmid, a PCR product encompassing both open reading frames was generated; in addition, a second plasmid was generated by inserting the *Cff*-*pglY* PCR product into the pCE111/28 (*Cff*-*pglX*) product via *Pst*I (introduced by PCR during the cloning of *pglX*) and *Xho*I simultaneously introducing an optimized RBS site upstream of the *Cff*-*pglY* start codon, as was done for all the other *pgl* genes. After ligation, transformation, and screening on selective (Cm) plates, plasmids isolated from candidate colonies were analyzed by restriction analyzes and verified by DNA sequencing. One positive candidate for each construct was used for further analysis.

### Pgl Operon Expression Plasmids

To generate p*pgl* operon mutant plasmids that are compatible with the generated *Cj* and *Cff*-*pgl* gene expression plasmids (pCE111/28-derivatives, Cm^R^), the *cat* cassette from all *pgl* operon plasmids with a *kan* cassette insertion in the various *pgl* genes (Supplementary Table S4; Linton et al., 2005) was deleted. To do so, plasmids p*pgl-pglH*:*kan*, p*pgl-pglI*:*kan*, p*pgl-pglJ*:*kan*, and p*pgl-pglA*:*kan* were treated with *Bsa*AI excising the *cat* gene but leaving the rest of the plasmid intact. The complete DNA digest reactions were purified and directly re-ligated. To generate the *pgl* operon plasmid lacking *pglH* and *pglI* (p*pgl-pglHI*:*kan*), two PCR products were generated: the first reaction was performed with plasmid p*pgl-pglH*:*kan* as a template and with oligonucleotides pglHI-kan-R and pglHI-pACYC-F amplifying the 5-prime half of the *kan* cassette in *pglH* and the upstream part of the *Cj-pgl* operon. The second reaction was performed with plasmid p*pgl-pglI*:*kan* as template and with oligonucleotides pglHI-aph-FR and pglHI-pACYC-R amplifying the 3-prime half of the *kan* cassette in *pglI*, the *pglI* downstream region of the *pgl* operon, as well as the origin of replication. The obtained PCR products were purified and ligated without further treatment.

After transformation of DH5α candidate colonies for each ligation reaction were pre-screened on LB agar for Kan^R^ and Cm^S^. The loss of the *cat* cassette and the correct gene organization on plasmids isolated form those colonies were further verified by restriction digest analyzes and DNA sequencing. One positive candidate for each construct (p*pgl*op *pgl-*gene*:kan*, cat- derivative) was used for further analyzes.

### Expression of CmeA-His_6_ in Glycosylation Competent *E. coli* Cells

Functional analysis of certain *Cff-pgl* proteins was performed in the heterologous *E. coli* glycosylation system. *E. coli* CLM24 was sequentially transformed with the individual *pgl* gene expression plasmids (pCE11/28 derivatives), the CmeA-His_6_ expression plasmid (pIH18, pEXT21-derivative), and either the plasmid carrying the WT *Cj-pgl* operon on p*pg*l or the compatible *pgl* operon mutant plasmids (*cat*- derivatives) with a *kan* cassette inserted into *pglA*, *pglJ*, *pglH*, and *pglHI* (double mutant). Cells stably maintaining the plasmid combinations were grown as 4 ml cultures overnight before inoculating 100 ml of fresh medium to a starting OD_600_ of 0.1. Cells were further grown until an OD_600_ of 0.5–0.7 was reached and CmeA-His_6_ expression (constitutively low expressed from the tetracycline promoter on pIH18) was further induced by the addition of IPTG to a final concentration of 0.5 mM. After growth for an additional 4 h, cells were cooled on ice for 10–15 min, pelleted by centrifugation (10 min, 12,000 rpm, 4°C), and washed twice with ice-cold 1 × PBS buffer. Then, 1/10 of the pellet (corresponding to 10 ml of culture volume) was used to produce whole cell lysates using Bacterial Protein Extraction Reagent B-PER (Thermo Fisher Scientific) according to the instructions of the manufacturer and the remainder of cells was used to generate whole cell lysates (as described in Liu et al., 2006) for the purification of the corresponding CmeA-His_6_ proteins by Ni-NTA affinity chromatography. To do so, whole cell extracts were filtered (0.22 μm) and loaded onto a 10 ml gravity-flow cartridge (Amersham Pharmacia Biosciences) pre-loaded with 0.5 ml Ni-NTA agarose and pre-equilibrated with 1 column volume 1 × PBS. The column was subsequently washed with at least 5 column volumes of 1 × PBS containing 20 mM imidazole and bound CmeA-His_6_ protein was eluted with 0.5–1.5 ml of PBS containing 0.5 M imidazole. Purified proteins were stored at 4°C until further use or immediately analyzed by 12.5% PAGE/mass spectrometry and/or western blotting.

### Hydrogen Growth Conditions

Growth of *Cff* under hydrogen was performed as previously described (Benoit and Maier, 2018) with the following changes. *Cff* cells were grown for 48 h then streaked on BHI plates and further incubated for 12 h at 37°C under microaerobic conditions. Cells were resuspended in BHI broth and standardized to the same optical density at 600 nm (OD_600_), 3.0–4.0. Sealed 165 mL bottles containing 10 mL BHI were flushed with N_2_ gas for 10 min, then CO_2_ (10% headspace partial pressure, h.p.p.) and O_2_ (5% h.p.p.) were injected in every bottle. H_2_ (20% h.p.p.) was added as indicated. Cells were inoculated (1:100) and grown at 37°C while shaking at 200 rpm. Growth yields from three biological replicates (each performed in duplicate) were determined after 48 h by serially diluting in BHI and plating on CBA. Plates were incubated at 37°C in microaerobic conditions for three days before being counted.

### Whole-Cell H_2_-Uptake Hydrogenase Assays

H_2_-uptake was performed as previously described (Maier et al., 1996). *Cff* cells were grown at 37°C for 24 h on BHI plates under microaerobic conditions either with 10% H_2_ or without. Cells were harvested and resuspended in phosphate buffered saline (PBS) to an optical density (OD_600_) of 1 which corresponds to ∼2.3 × 10^9^ cells/mL. A 2 mL chamber was filled with cells followed by an injection with PBS saturated with H_2_. H_2_-uptake was monitored as previously described (Maier et al., 1996). Values are reported as nanomoles of H_2_ used per min per 10^9^ cells and represent four independent measurements for cells grown in microaerobic conditions (and no H_2_) and two measurements for cells grown in microaerobic conditions with the addition of 10% H_2_.

### Determination of Iron and Nickel Content

*Campylobacter fetus* subsp. *fetus* cells were grown at 37°C for 24 h on two BHI plates under microaerobic conditions and harvested with a loop in 1 mL metal-free double distilled water. Samples were centrifuged at 10,000 *g* for 5 min, washed once with water, resuspended and lyzed by sonication. A portion of lyzed sample was used to determine the protein concentration using the bicinchoninic acid (BCA, Thermo Scientific Pierce) assay. Samples were centrifuged at 15,000 × *g* for 5 min and the supernatant was analyzed for iron and nickel. The remaining sample portion was used for metal (Fe or Ni) content analysis. Briefly, Fe and Ni concentrations were measured by atomic absorption, using a Shimadzu AA-6701F spectrophotometer. All samples were diluted (in 1% HNO_3_) to be in the range of the standard curve (0 to 0.4 μM of either Fe or Ni) generated using atomic absorption-grade standard Fe or Ni solutions (Sigma). Results shown are means and standard deviations for 3–5 measurements.

### Ethidium Bromide Accumulation Assay

Accumulation of EtBr was performed as previously described (Lin et al., 2002) with the following changes. *Cff* strains were grown overnight on BHI agar at 37°C in microaerobic conditions and harvested with MEM (Gibco). Cultures were adjusted to OD_600_ of 0.2 and then incubated at 37°C for 30 min in microaerobic conditions. EtBr was added to a final concentration of 2 μg/mL. Fluorescence was measured, with an excitation of 530 nm and emission of 600 nm, every 2 min over a 20 min time using a Bio Tek Synergy H1 plate reader. This was performed in three biological replicates, which included three technical replicates. Background fluorescence of MEM with EtBr was subtracted from these values.

### Antibiotic MIC Assay

*Campylobacter fetus* subsp. *fetus* cells were grown for 24 h at 37°C in microaerobic conditions on CBA plates. Antibiotic MIC was assessed using the Sensititre (Trek Diagnostic Systems) platform. Sensititre plate EQUIN1F was used, following manufacturer’s instructions.

### Preparation of Bacterial Whole Cell Proteome Samples

*Campylobacter fetus* subsp. *fetus* cells were grown for 24 h at 37°C under microaerobic conditions on BHI agar. Cells were harvested with ice-cold PBS and inactivated with PBS, 10% sodium azide for 30 min at 4°C. Cell pellets obtained after centrifugation (4000 × *g* for 15 min) were lyophilized and stored at −20°C until further use. Cell lysates for proteomic analyses were prepared as follows: cells were solubilized in 4% SDS, 100 mM Tris pH 8.0, and 20 mM DTT and boiled at 95°C with shaking at 2000 rpm for 10 min. Insoluble material was removed by centrifugation at 17,000 × *g* for 10 min at room temperature and the supernatant was collected. Protein concentrations were determined using the bicinchoninic acid assay (Thermo Scientific Pierce) and 200 μg of protein from each sample was acetone-precipitated overnight at −20°C by mixing volumes of ice-cold acetone with one volume of sample. Samples were then spun down at 16,000 × *g* for 10 min at 4°C. The precipitated protein pellets were resuspended with 80% ice-cold acetone and precipitated for an additional 4 h at −20°C. Samples were spun down at 17,000 × *g* for 10 min at 4°C to collect the precipitated protein.

### Digestion of Complex Protein Lysates

Dried protein pellets were resuspended in 6 M urea, 2 M thiourea, 40 mM NH_4_HCO_3_ and reduced/alkylated prior to digestion with Lys-C (1/200 w/w) and then trypsin (1/50 w/w) overnight as previously described (Scott et al., 2011). Digested samples were acidified to a final concentration of 0.5% formic acid and desalted with home-made high-capacity StageTips composed on 5 mg Empore^TM^ C18 material (3M, Maplewood, Minnesota) and 5 mg of OLIGO R3 reverse phase resin (Thermo Fisher Scientific) according to the protocol of Ishihama and Rappsilber (Ishihama et al., 2006; Rappsilber et al., 2007). Bound peptides were eluted with buffer B, dried and stored at −20°C.

### Reversed Phase Liquid Chromatography-Mass Spectrometry

Purified peptides were resuspended in Buffer A^∗^ and separated using a two-column chromatography set up comprising a PepMap100 C18 20 mm × 75 μm trap and a PepMap C18 500 mm × 75 μm analytical column (Thermo Fisher Scientific). Samples were concentrated onto the trap column at 5 μl/min for 5 min and infused into an Orbitrap Elite^TM^ Mass Spectrometer (Thermo Fisher Scientific) at 300 nl/min via the analytical column using a Dionex Ultimate 3000 UPLC (Thermo Fisher Scientific). Then, 180 min gradients were run altering the buffer composition from 3% buffer B to 28% B over 150 min, then from 28% B to 40% B over 10 min, then from 40% B to 100% B over 2 min, followed by the composition held at 100% B for 3 min, and then dropped to 3% B over 5 min and held at 3% B for another 10 min. The Orbitrap Mass Spectrometer was operated in a data-dependent mode automatically switching between the acquisition of a single Orbitrap MS scan (60,000 resolution) followed by one data-dependent HCD (resolution 15 k AGC target of 4 × 10^5^ with a maximum injection time of 250 ms, NCE 40) and CID (ion trap, AGC target of 5 × 10^4^ with a maximum injection time of 100 ms, NCE 35) event for each precursor (total of five precursors per cycle with 45 s dynamic exclusion enabled).

### Proteome Data Analyses

Proteome analysis to assess the expression of proteins within *Cff* strains was undertaken with MaxQuant [v1.5.3.30 (Cox and Mann, 2008)]. Database searching was carried out against the *C. fetus* subsp. *fetus* strain ATCC 27374 proteome (generated from a Maxquant generated six frame translation of the in-house sequenced strain). Searches were undertaken with the following search parameters: carbamidomethylation of cysteine as a fixed modification; oxidation of methionine, acetylation of protein N-terminal trypsin/P cleavage with a maximum of two missed cleavages. To enhance the identification of peptides between samples, the Match between Runs option was enabled with a precursor match window set to 2 min and an alignment window of 10 min. For label free quantitation, the MaxLFQ option within Maxquant was enabled in addition to the re-quantification module (Cox et al., 2014). The resulting outputs were processed within the Perseus (v1.5.0.9) analysis environment to remove reverse matches and common proteins contaminations prior to further analysis (Tyanova et al., 2016). Statistical analysis was undertaken in Perseus by grouping biological replicates, imputing missing values based on observed values (downshifted by 2.5 standard deviations with a width of 0.3 standard deviations) and then comparing groups using a student *t*-test. To define an appropriate *p*-value threshold, multiple hypothesis correction was undertaken using a Benjamini-Hochberg correction with a FDR of 0.05. All statistical outputs are provided within Supplementary Table S1. All mass spectrometry proteomics data have been deposited to the ProteomeXchange Consortium via the PRIDE partner repository (Vizcaino et al., 2016) with the dataset identifier PXD014538 [LFQ experiments of *C. fetus fetus* mutants (Supplementary MS Data 1) accessible using the **username:** reviewer71456@ebi.ac.uk, **password:** B5YuYNx8) and PXD017832 [analysis of *C. fetus fetus pgl* enzymes in the heterologous *E. coli* glycosylation system (Supplementary MS Data 2) accessible using the **username:** reviewer23740@ebi.ac.uk **password:** PHKlhnSp].

### Glycopeptide Data Analysis

Glycopeptides were identified by manually interrogating possible glycopeptide scans based on the presence of the diagnostic oxonium ion (204.09 m/z) of HexNAc. To facilitate glycopeptide assignments from HCD scans, the ions below the mass of the predicted deglycosylated peptides were extracted with Xcalibur v2.2 using the Spectrum list function. Ions with a deconvoluted mass above that of the deglycosylated peptide and ions corresponding to known carbohydrate oxoniums were removed in a similar approach to post-spectral processing of ETD data and then searched with Mascot^1^. Searches were carried out using semi-trypsin specificity, carbamidomethylation of cysteine as a fixed modification, and oxidation (M) as a variable modification. A precursor and product tolerance of 20 ppm was used, and the taxonomy restricted to “Other Proteobacteria.” All spectra were searched with the decoy option enabled with all peptides passing a 1% FDR. Identified glycopeptide spectra were manually inspected and spectra annotated according to the nomenclature of Roepstorff and Fohlman (1984) for peptides as well as Domon and Costello (1988) for glycans.

## Data Availability Statement

The mass spectrometry proteomics data have been deposited to the ProteomeXchange Consortium via the PRIDE (1) partner repository with the dataset identifiers PXD014538 and PXD017832.

## Author Contributions

JD, HN, and CS designed the experiments, interpreted the results, and wrote the manuscript. BB and CF constructed expression plasmids and *Cf-pgl* mutants. SB and RM performed the hydrogenase activity and assisted with analysis and interpretation of hydrogenase and AAS data. NS performed all mass spectrometry and LFQ analysis. DW and DL made *pgl* expression constructs for *E. coli* and assisted in data analysis and interpretation. All authors read and approved of the final manuscript.

## Conflict of Interest

The authors declare that the research was conducted in the absence of any commercial or financial relationships that could be construed as a potential conflict of interest.

## Footnotes

1 http://www.matrixscience.com/, v2.5

## Nomenclature

*Cf*: *Campylobacter fetus*
*Cff*: *Campylobacter fetus* subsp. *fetus*
*Cft*: *Campylobacter fetus* subsp. *testudinum*
*Cfv*: *Campylobacter fetus* subsp. *venerealis*
*Cj*: *Campylobacter jejuni*
diNAcBac: 2,4-diacetamido-2,4,6-trideoxyglucopyranose
fOS: free oligosaccharides
GalNAc: *N-acetyl*-galactosamine
Glc: glucose
GlcNAc: *N-acetyl*-glucosamine
GTase: Glycosyltransferase
Hex: hexose
HexNAc: *N-acetyl*-hexosamine
LLO: lipid-linked oligosaccharide
MS: mass spectrometry.
