# Supplementary material for: Influence of Protein Glycosylation on Campylobacter fetus Physiology
Source: Front Microbiol. 2020 Jun 17;11:1191. doi: 10.3389/fmicb.2020.01191 (PMC7313396; doi:10.3389/fmicb.2020.01191)
Supplement: Supplementary file 2 [file Presentation_1.pdf]

Supplementary Material

Results

PglA and PglJ from *Cff* and *Cj* have similar functions

Mass spectrometric analyses of trypsinated CmeA glycopeptides produced in the heterologous *E. coli* system were carried out to confirm the glycosyltransferase activities of *Cff*-PglA and PglJ observed in our western blot analyses. Full length *Cj*-N-glycan on the CmeA peptide (93-ATFENASKDF**N**R-104, m/z = 467.2251, z=3 or m/z = 700.3337, z=2, modified asparagine residue is in bold) was observed upon expression of *Cff*-PglA and *Cff*-PglJ in the respective *Cj-pgl* operon mutants (*pglA*^{mut} + *Cff-pglA* and *pglJ*^{mut} + *Cff-pglJ*). Here, glycopeptides with identical m/z and N-glycan compositions (diNAcBac-HexNAc$_5$-Hex) as the presence of the intact *Cj-pgl* operon (*pgl*^{WT}) or when the specific *pgl* operon mutation was complemented with the respective *Cj-pgl* genes (positive controls) were observed (*pglA*^{mut} + *Cj-pglA* and *pgJj*^{mut} + *Cj-pglJ*) (Supplementary MS data 2). This clearly indicates that PglA and PglJ proteins from *Cff* and *Cj* have identical functions that are transferring the first and the second GalNAc residue to the lipid-linked oligosaccharide (LLO) precursor, UndPP-diNAcBac, respectively. N-glycans observed upon expression of PglA and PglJ from either *Cff* or *Cj* in combination with the *Cj-pgl* operon lacking *pglJ* or *pglA* (samples *pglA*^{mut} + *Cff-pglJ, pglA*^{mut} + *Cj-pglJ, pglJ*^{mut} + *Cff-pglA and pglJ*^{mut} + *Cj-pglA*) were similar in composition and sequence when compared to the N-glycans produced in the control samples i.e. in the absence of the complementation plasmids (*pglA*^{mut} and *pglJ*^{mut}). Here, masses corresponding to the CmeA peptide (93-ATFENASKDF**N**R-104) with diNAcBac-HexNAc and diNAcBac-HexNAc$_2$ were detected (in agreement with the expected N-glycan products). As expected, only unglycosylated CmeA peptides were present in the absence of *pgl* genes.

Analysis of N-glycans produced by *Cff*-PglX and *Cff*-PglY in the heterologous *E. coli* system

To determine the N-glycan structures produced upon expression of *Cff-pglX* and *pglY* in the presence of defined *Cj-pgl* operon mutants (lacking *pglH*, *pglI* or *pglHI*) in *E. coli*, mass spectrometric analyses of CmeA glycopeptides was carried out (Supplementary MS data 2). We found that full length *Cj*-N-glycan was added to CmeA (93-ATFENASKDF**N**R-104) when the *pglH* mutation on the *Cj-pgl* operon was complemented with *Cj-pglH* (*pglH*^{mut} + *Cj-pglH*, positive control), that (as expected) diNAcBac-GalNAc$_2$ was produced in the absence of the complementation plasmid (*pglH*^{mut}) and that no glcyopeptides were observed in the absence of the *Cj-pgl* operon (negative control). Upon expression of *Cff-pglX* in p*pgl-pglH*::*kan* cells (*pglH*^{mut} + *Cff-pglX)* we detected the unmodified tri-saccharide substrate (diNAcBac-HexNAc$_2$) as well as tri-saccharide substrate modified with Hex, HexNAc-Hex$_2$ or HexNAc-Hex. Similarly upon expression of *pglY* alone (*pglH*^{mut} + *Cff-pglY)* we detected the formation of incomplete substrate (diNAcBac-HexNAc), unmodified substrate (diNAcBac-HexNAc$_2$) and substrate with one or two hexoses added to the second HexNAc of the N-glycan. Expression of *pglX* and *pglY* (*pglH*^{mut} + *Cff-pglXY* and *pglH*^{mut} + *Cff-pglX+Y)* resulted in the addition of Hex, HexNAc-Hex and HexNAc-Hex$_2$ (also observed upon expression of *pglX* alone). In addition we observed the addition of a single HexNAc as well as the addition of HexNAc$_2$ or HexNAc$_2$-Hex resulting in the formation of a diNAcBac-HexNAc$_4$ penta-

and a diNAcBac-HexNAc$_4$-Hex hexasaccharide; the latter composition would be consistent with the formation of the minor form of the native *Cff*-N-glycan structure.

In the p*pgl-pglI*::*kan* control strain the absence of a complementation plasmid (sample *pglI*^{mut}) or expression of *Cj-pglH*, (*pglI*^{mut} + *Cj-pglH*) led to the formation of diNAcBac-HexNAc$_5$ (as expected) and also diNAcBac-HexNAc$_6$. For the latter case it is worth mentioning that the addition of 4 HexNAc units by *Cj*-PglH was also observed by Ramirez and colleagues (*in vitro* and in the absence of PglI) and might be an artefact of the experimental conditions (Ramirez et al., 2018). Expression of *pglX* (*pglI*^{mut} + *Cff-pglX*) not only resulted in the formation of the substrate diNAcBac-HexNAc$_5$ (and diNAcBac-HexNAc$_6$) but also in shorter diNAcBac-HexNAc$_3$ and diNAcBac-HexNAc$_3$-Hex N-glycans, the latter structure indicates the addition of a Hex residue to an incomplete *Cj*-N-glycan *pglI* structure by PglX. Expression of PglY alone (*pglI*^{mut} + *Cff-pglY*) resulted in the formation of diNAcBac-HexNAc$_5$, diNAcBac-HexNAc$_6$ as well as in a shorter diNAcBac-HexNAc$_4$ N-glycan variant. Expression of PglX and PglY (*pglI*^{mut} + *Cff-pgXY, pglI*^{mut} + *Cff-pglX+Y*) resulted in the formation of diNAcBac with 3, 4, 5, or 6 HexNAc residues attached, representing the identical N-glycans formed in the absence of a complementation plasmid (*pglI*^{mut}) and shorter versions thereof. In the *pglHI* mutant background (*pglHI*^{mut}) we predominantly found diNAcBac with 2, 3, 4, or 5 HexNAc residues upon expression of *Cj-pglH* (*pglHI*^{mut} + *Cj-pglH*), PglX, PglY and PglX/PglY (samples *pglHI*^{mut} + *Cff-pglX, pglHI*^{mut} + *Cff-pglY*, *pglHI*^{mut} + *Cff-pglXY* and *pglHI*^{mut} + *Cff-pglX+Y*), however, in the absence of the complementation plasmid a diNAcBac-HexNAc$_2$-Hex variant could be seen. This was unexpected since no additional *pgl* gene was present that could explain the addition of the Hex residue that might therefore be added by an *E. coli* GTase; however, upon expression of *Cff-pglX* (*pglHI*^{mut} + *Cff-pglX*) we also detected a diNAcBac-HexNAc$_3$-Hex N-glycan variant indicating that this hexose residue could also be a product of this *Cff*-GTase.

Material and Methods

Digestion of CmeA

Isolated CmeA bands were processed as previously described (Shevchenko et al., 2006) with minor modifications. Briefly, CmeA-His$_6$ was enriched by Ni-NTA gravity flow as described (Feldman et al., 2005) and subsequently separated by 12% PAGE followed by Coomassie straining. CmeA-His$_6$ bands were excised and destained in a 50:50 solution of 50 mM NH_4HCO_3: 100% ethanol for 20 min at room temperature with shaking at 750 rpm. Destained bands were washed with 100% ethanol for 10 min at 750 rpm for dehydration and then rehydrated in 10 mM DTT in 50 mM NH_4HCO_3. Reduction was carried out for 60 min at 56°C with 750 rpm shaking. The reduction buffer was then removed, and the gel bands washed twice in 100% ethanol for 10 min to ensure the removal of DTT. Reduced ethanol washed samples were sequentially alkylated with 55 mM iodoacetamide in 50 mM NH_4HCO_3 in the dark for 45 min at RT. Alkylated samples were then washed with 2 rounds of 100% ethanol and then vacuum-dried. Alkylated samples were then rehydrated with 12 ng μl^{-1} trypsin (Promega, Madison WI) in 40 mM NH_4HCO_3 at 4°C for 1 hr. Excess trypsin was removed, gel pieces were covered in 40 mM NH_4HCO_3 and incubated overnight at 37°C. Peptides were concentrated and desalted using C_{18} stage tips (Ishihama et al., 2006; Rappsilber et al., 2007) and stored on tips at 4°C. Peptides were eluted in buffer B (0.1% formic acid, 80% MeCN) and dried before analysis by LC-MS.

Identification of glycosylated peptides using reversed phase LC-MS

Purified CmeA peptides prepared were resuspended in Buffer A* (2% acetonitrile, 0.1% TFA) and separated using a two-column chromatography set up composed of a PepMap100 C18 20 mm x 75 µm trap and a PepMap C18 500 mm x 75 µm analytical column (Thermo Fisher Scientific). Samples were concentrated onto the trap column at 5 µL/min for 5 minutes and infused into an Orbitrap Fusion™ Lumos™ Tribrid™ Mass Spectrometer (Thermo Fisher Scientific) at 300 nl/min via the analytical column using a Dionex Ultimate 3000 UPLC (Thermo Fisher Scientific). Then, 60 min gradients were run altering the buffer composition from 1% buffer B (0.1% formic acid, 80% MeCN) to 28% B over 35 min, then from 28% B to 40% B over 10 min, then from 40% B to 100% B over 2 min, the composition was held at 100% B for 3 min, and then dropped to 3% B over 5 min and held at 3% B for another 10 min. The Lumos™ Mass Spectrometer was operated in a data-dependent mode automatically switching between the acquisition of a single Orbitrap MS scan (120,000 resolution) every 3 seconds and Orbitrap HCD scans for each selected precursor (NCE 28, maximum fill time 100 ms, AGC $4*10^4$ with a resolution of 15000). For MS/MS events observed to contain the HexNAc oxonium ion 204.0867 three additional scans were triggered; One Orbitrap EThcD MS-MS scan (NCE 25; maximum fill time 250 ms, AGC $2*10^5$ with a resolution of 30000); One Orbitrap HCD MS-MS scan (stepped NCE of 25, 40 and 48, maximum fill time 250 ms, AGC $2*10^5$ with a resolution of 30000) and a ion-trap CID scan (NCE 35, maximum fill time 50ms).

Mass spectrometry data analysis of CmeA glycosylation

Identification of CmeA glycoprotein samples was accomplished using MaxQuant (v1.6.3.4) (Cox and Mann, 2008). Searches were performed against the CmeA sequence (Uniprot sequence: Q0PBE3) with carbamidomethylation of cysteine set as a fixed modification. Searches were performed with trypsin cleavage specificity allowing 2 mis-cleavage events and the variable modifications of oxidation of methionine, the *Campylobacter* glycan mass (elemental composition C56H91N7O34; Asn) and acetylation of protein N-termini. The precursor mass tolerance was set to 20 parts-per-million (ppm) for the first search and 10 ppm for the main search, with a maximum false discovery rate (FDR) of 1.0% set for protein and peptide identifications. Glycopeptides of CmeA were identified by manually interrogating possible glycopeptide scans based on the presence of the diagnostic oxonium ion (204.09 m/z) of HexNAc. For the CmeA glycopeptide ATFENASKDF**N**R glycopeptide ion intensities for the + 2 and +3 charge states were manually extracted and used to assess relative glycoform abundance. Representative annotated spectra for each glycoform and charge stated identified by MS/MS is provided (Supplementary MS data 2).

Figure S1

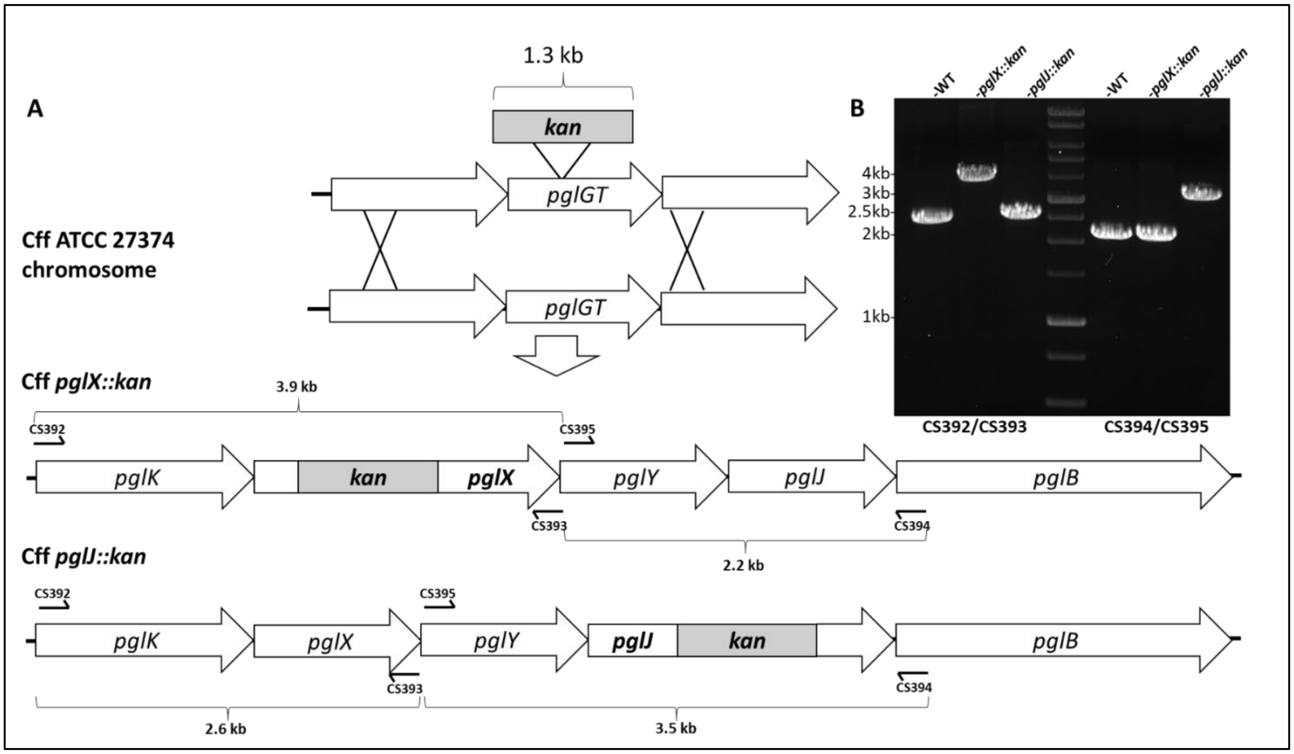

Figure S1. Insertional mutagenesis of *pglX* and *pglJ* in *C. fetus* subsp. *fetus* ATCC 27374. (A) The *pgl* genes were inactivated by insertion of the kanamycin resistance gene (*kan*) into the chromosome of *C. fetus* subsp. *fetus* ATCC 27374. PCR products were introduced by electroporation and double-crossover events resulted in the formation of *pglX*- and *pglJ*-. Primer sets CS392/CS393 and CS394/395, were used to confirm the correct insertion of the antibiotic cassette. **(B)** Agarose gel with PCR products amplified to verify the insertion of the cassette. Genomic DNA from WT or the respective *pgl* mutant strain that was used as template is indicated above each lane. The primer set used for each PCR reaction is indicated below the gel.

Figure S2

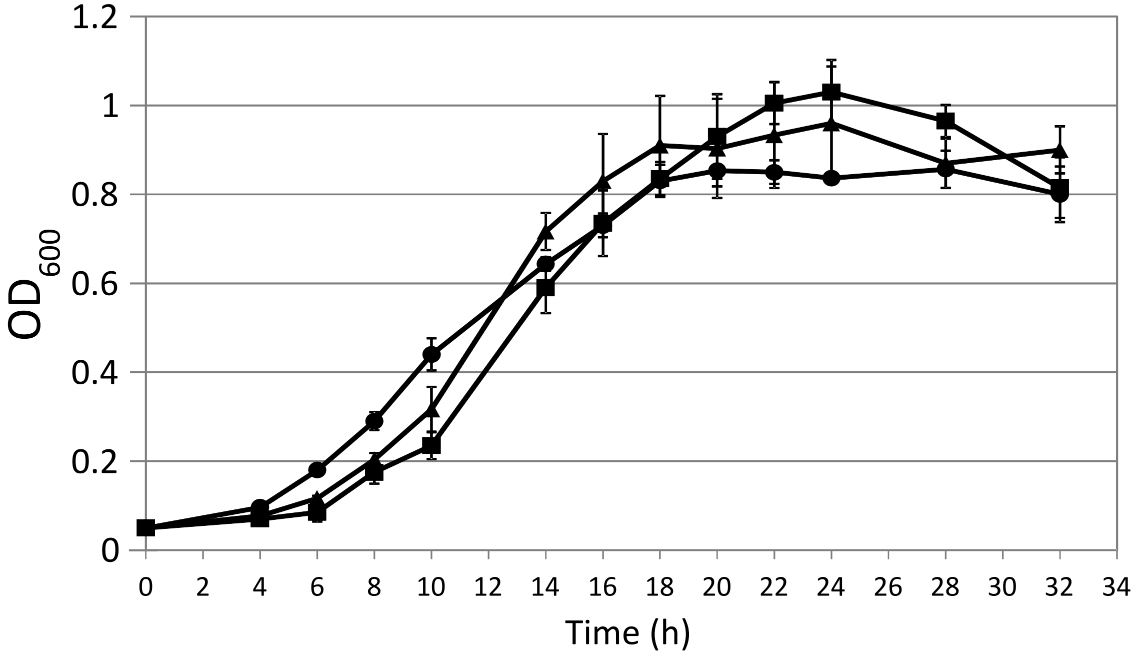

Figure S2. ***In vitro* growth of *Cff*.** *Cff* wildtype (circles), the *pglX* mutant (squares) and the *pglJ* mutant (triangles) were grown in BHI broth under microaerobic conditions with shaking at 37°C. Growth curves were recorded at OD_{600} over a time frame of 32 h. Results shown represent the average values from three independent biological replicates. The error bars indicate standard deviation for each data point.

Figure S3

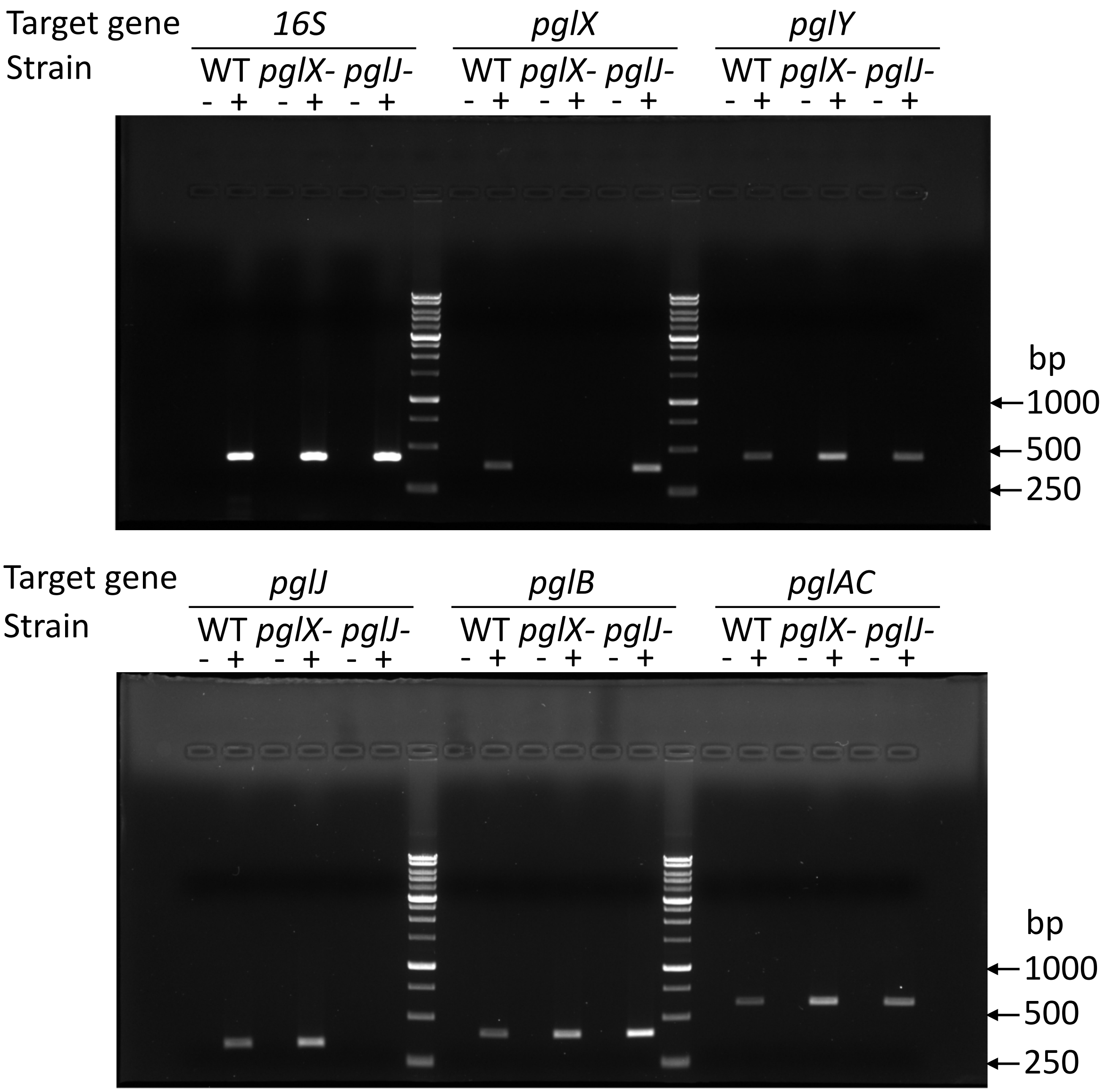

Figure S3. RT-PCR analyses of *pgl* genes. *Pgl* gene-specific PCR products obtained after reverse transcription of purified RNA from the *Cff* wild-type (WT), the *pglX* (*pglX-)* and the *pglJ* (*pglJ-)* mutant strains were analyzed by 0.8% agarose gel electrophoresis. (+) indicates RNA reverse transcribed with SuperScript, (-) indicates the no-SuperScript RT control. No polar effects were observed on the transcription of the genes downstream (target, as indicated) after integration of the *kan* cassette in either *pglX*- or *pglJ*-. Significant bands of the DNA standard (in base pairs, bp) are indicated on the left. The obtained gene-specific PCR products were in agreement with the expected sizes for 16S (421 bp), *pglJ* (330 bp), *pglY* (443 bp), *pglX* (371 bp), *pglB* (399 bp) and *pglAC* (609 bp).

Figure S4

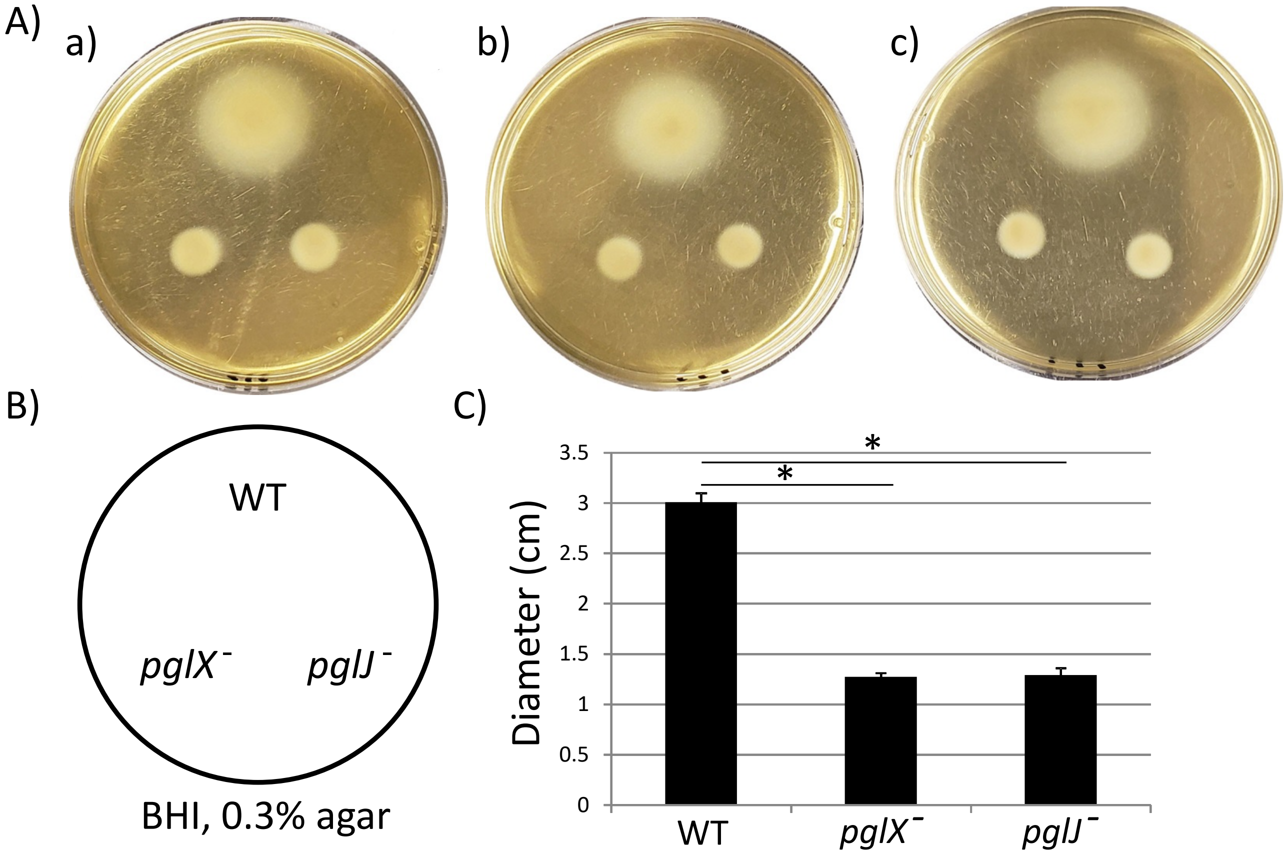

Figure S4. Mutation of *pglX* or *pglJ* resulted in reduced motility. (A) Triplicates of 0.3% BHI swarm agar plates **(a, b, c)** are shown at 60% of their original size. The low percentage of the agar allows the bacteria to swim and form a zone of growth around the point of inoculation. All three strains (*Cff*-wildtype (WT), the *pglX* (*pglX*-) and the *pglJ* (*pglJ*-) mutant) were analyzed in parallel (as depicted in **(B)**) on each plate to exclude plate-to-plate variations. **(C)** The average diameter (in cm) of the halo for each strain is depicted, standard deviations are indicated by error bars, statistically significant differences (p-value ≤ 0.001 analyzed by a two-tailed t- test) are indicated by an asterisk.

Figure S5

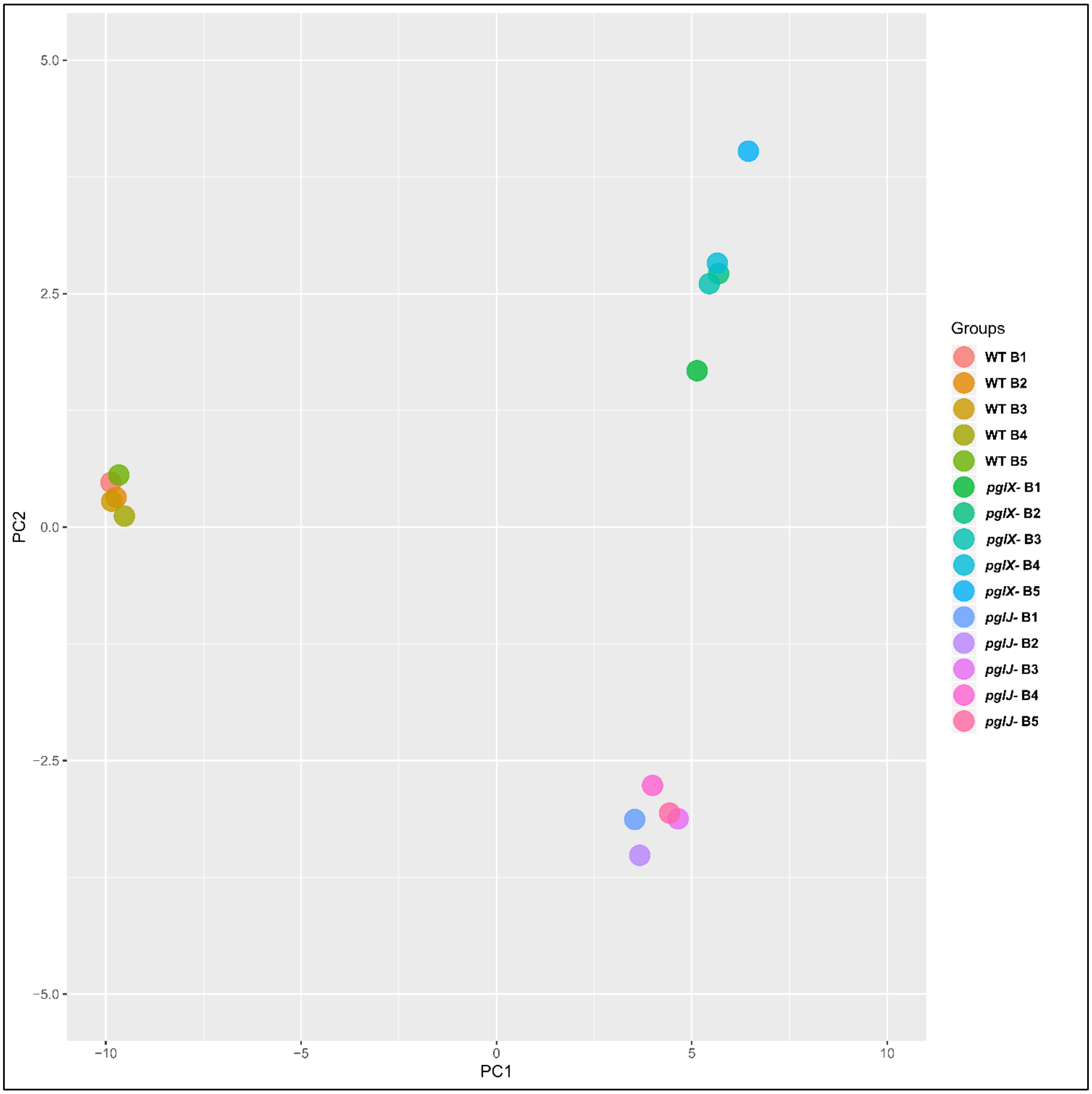

Figure S5. Principal component analysis (PCA) of LFQ proteome analysis of *C. fetus* subsp. *fetus* ATCC 27374 WT, *pglJ* and *pglX* mutants. PCA analysis reveals segregation of each sample group (5 replicates (B1-5) for WT, *pglX*- and *pglJ*-) as indicated by the colour scheme.

Figure S6

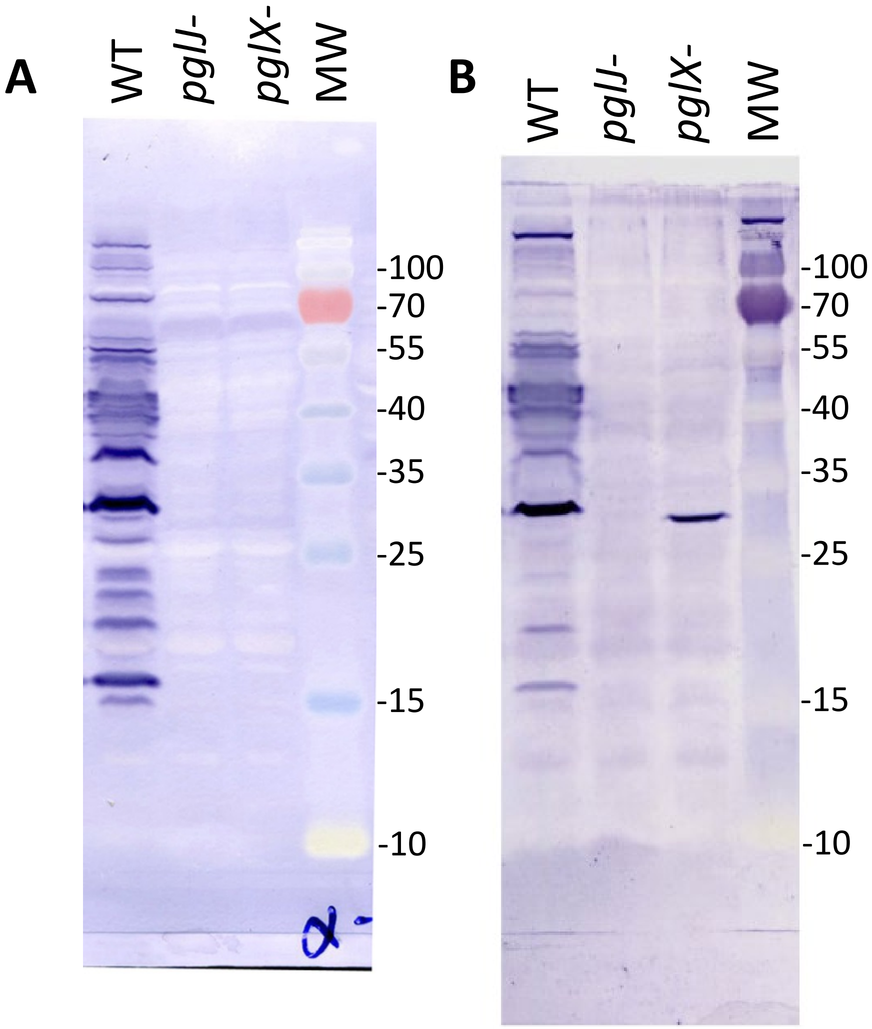

Figure S6. Pgl pathway product analysis of *Cf* WT and *pglX-* and *pglJ-* strains. Full scans (top to bottom) of the original western blots shown in Figure 3A and B are provided. **(A)** Western blot of whole-cell lysates with *Cff*-N-glycan-specific antiserum, and **(B)** wheat germ agglutinin reactivity of whole cell lysates of the WT and the *pglJ-* and *pglX-* strains are shown.

Figure S7A

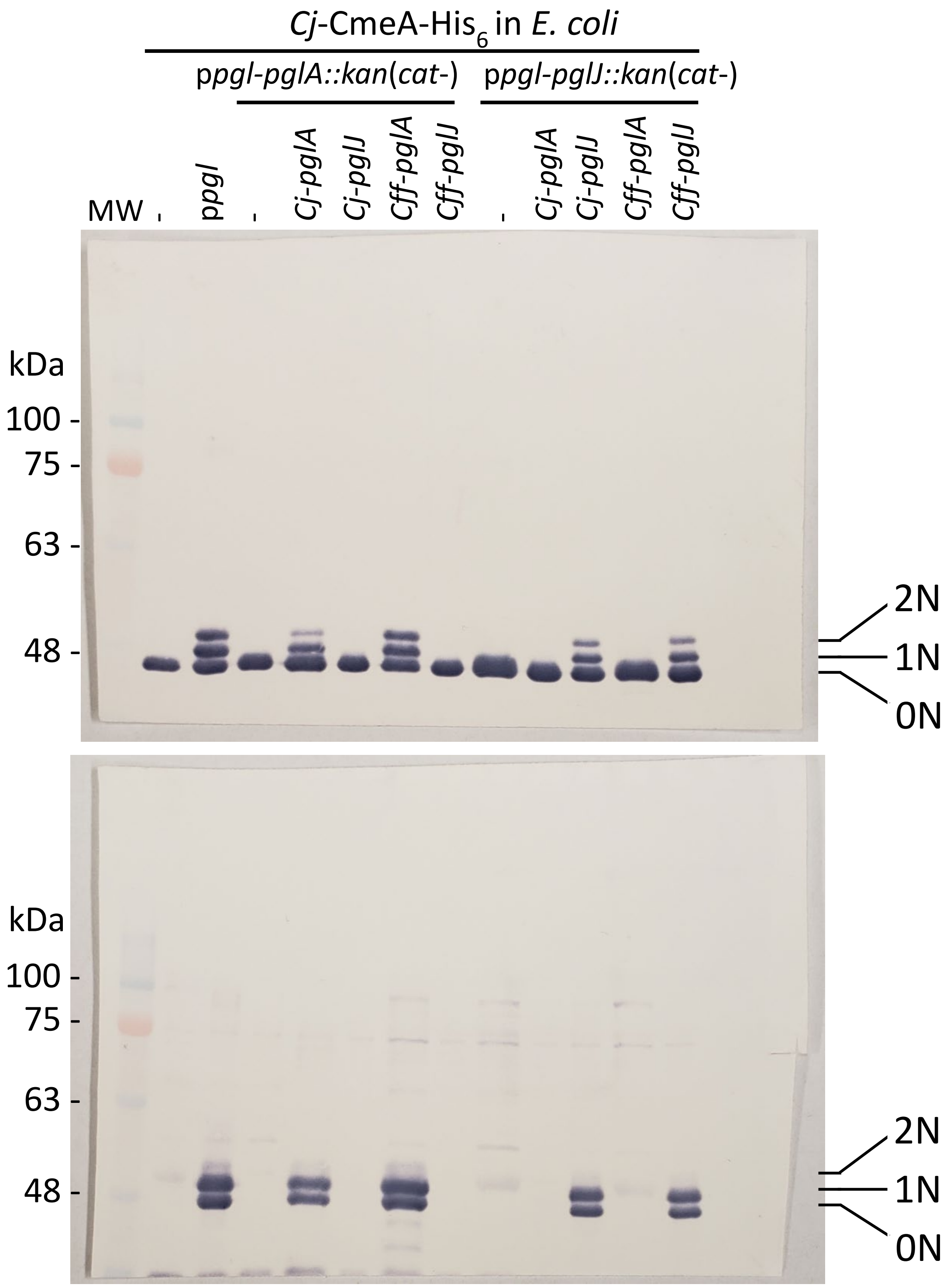
Cj-CmeA-His6 in E. coli
ppgl-pglA::kan(cat-)
ppgl-pglJ::kan(cat-)
MW
-
ppgl
-
Cj-pglA
Cj-pglJ
Cff-pglA
Cff-pglJ
-
Cj-pglA
Cj-pglJ
Cff-pglA
Cff-pglJ
kDa
100 -
75 -
63 -
48 -
2N
1N
0N
kDa
100 -
75 -
63 -
48 -
2N
1N
0N

Figure S7B

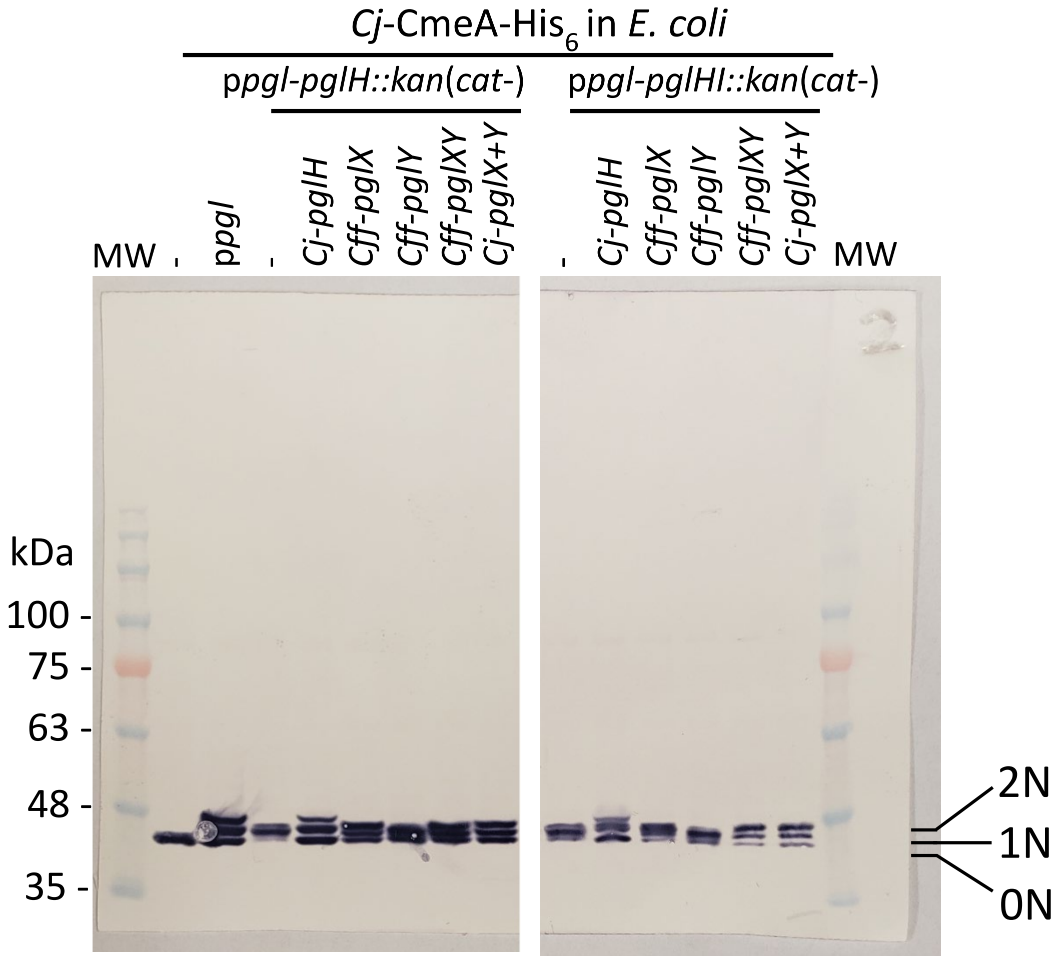

Figure S7. Functional analysis of *Cff-pgl* pathway glycosyltransferases (GTases) in the heterologous *E. coli* glycosylation system. Full scans (top to bottom) of the original western blots shown in Figure 5A and B are provided**.** The GTase-activities of **(A)** PglA and PglJ were analyzed in western blots of CmeA-His$_6$ with His$_6$-tag (upper panel) and *Cj*-N-glycan specific (lower panel), R1 antibodies. **(B)** PglX and PglY activities were analyzed with His$_6$-tag specific antibodies in western blots of CmeA-His$_6$ used as the glycan acceptor to determine N-glycosylation activities. Whole cell extracts (5 µg) of *E. coli* CLM24 expressing the indicated mutant plasmid/*pgl* gene combinations are indicated above each lane. None, mono, and di-glycosylated CmeA-His$_6$ proteins are labelled as 0N, 1N and 2N, respectively. Molecular weight markers (MW) in kDa are indicated on the left.

Table S2. Antibiotic resistance of *Cff* WT, *pglX*- and *pglJ*- strains

**Antibiotic**	***WT***	***pglJ-***	***pglX-***
Amoxicillin / Clavulanate	2	<= 1	<= 1
Ampicillin	4	<= 1	<= 1
Azithromycin	0.5	0.25	0.5
Cefoxitin (2nd gen.)	32	>32	32
Ceftiofur (3rd gen.)	>8	> 8	> 8
Chloramphenicol	8	4	4
Ciprofloxacin	0.5	0.5	0.5
Gentamicin	2	1	1
Sulfisoxazole	256	128	256
Tetracycline	<=4	<= 4	<= 4
Trimethoprim / Sulfamethoxazole	>4	4	>4

Sensititre™ was used to assess the minimum inhibitory concentration (MIC) of the indicated *Cff* strains. The data (in $\mu g/ml^{-1}$) represent one assay done at the Athens Veterinary Diagnostic Facility.

Table S3. Oligonucleotides used in this study.

**Oligonucleotide**	**Primer sequence (5' → 3')**	**Application**
CS469	TAAACCTGCAGGAAATTACCCAAGTTTTTCATAAATTTCCTC	PCR product to construct *Cff-pgl* mutants
CS470	TTTCTCGAGATTTACTCATAAACATAAGTTTCATTTGC	PCR product to construct *Cff-pgl* mutants
CS392	ATTTCTAGAAGGAGAGTTTGCAATGGCTTTAAATCTTATAGCAAGTATCG	Confirm *Cff-pglX::kan*
CS393	ATATGGATCCTCATTTTGCTGCTTCTTTTATCAAATTCAACC	Confirm *Cff-pglX::kan*
CS394	ATAAACTGCAGTTACCCAAGTTTTTCATAAATTTCCTCTAAATC	Confirm *Cff-pglJ::kan*
CS395	AAGGATCCAAAAGAAGCAGCAAAATGAATATAATCTTTTTTATTTCAGC	Confirm *Cff-pglJ::kan*
Cj-pglJ-F	ATAGGATCCTAA*GAAGGAG*ATATACAT**ATG**CAAAAATTAGGCATTTTTATTTATTC, *BamH*I	Clone *Cj-pglJ*
Cj-pglJ-R	TTCTCGAGCTATCCTAATAAATATTTCAAAGCATCGCGTGC, *Xho*I	Clone *Cj-pglJ*
CFF-pglJ-F	ATAGGATCCTAA*GAAGGAG*ATATACAT**ATG**AAAAAGTTAAGTGTTTTTATATATTC, *BamH*I	Clone *Cff-pglJ*
CFF-pglJ-R	TTCTCGAGCTACCCAAGTTTTTCATAAATTTCCTCTAAATC, *Xho*I	Clone *Cff-pglJ*
Cj-pglA-F	ATAGGATCCTAA*GAAGGAG*ATATACAT**ATG**AGAATAGGATTTTTATCACATGCAGG, *BamH*I	Clone *Cj-pglA*
Cj-pglA-R	TTCTCGAGCTATACATTCTTAATTACCCTATCATAAAGTTTTAAATAACG, *Xho*I	Clone *Cj-pglA*

CFF-pglA-F	ATAGGATCCTAA*GAAGGAG*ATATACAT**ATG**AAAATAG GTTTTTTATCACATTCG, *BamH*I	Clone *Cff-pglA*
CFF-pglA-R	TTCTCGAGCTATACATCTATAAATTCCTTATAAACTTCG AGATATTGTTTAG, *Xho*I	Clone *Cff-pglA*
Cj-pglH-F	ATAGGATCCTAA*GAAGGAG*ATATACAT**ATG**AAAATAA GCTTTATTATCGC, *BamH*I	Clone *Cj-pglH*
Cj-pglH-R	TTCTCGAGCTAGGCATTTTTAACCTCGACTATAAGCTT AAGCC, *Xho*I	Clone *Cj-pglH*
CFF-pglX-F	ATAGGATCCTAA*GAAGGAG*ATATACAT**ATG**AAAGTTTT ATTTATCATCT, *BamH*I	Clone *Cff-pglX*
CFF-pglX-R	TT**CTCGAG**AATACGTCTGCAGTCAATTTGCTGCTTCTT TTATCAAATTCAACC, *Pst*I, *Xho*I	Clone *Cff-pglX*
CFF-pglY-F	ATACTGCAGTAA*GAAGGAG*ATATACAT**ATG**GTTGAATT TGATAAAAGAAGC, *Pst*I	Clone *Cff-pglY*
CFF-pglY-R	TTCTCGAGTCATTGTTCGCACTCTTTTATAAATTTATAC CATTTATCTATG, *Xho*I	Clone *Cff-pglY*
pglHI-aph-F	/5Phos/TTAAAGACGGAAAAGCCCGAAGAGGAACTTG	Construct *ppglHI::kan*
pglHI-aph-R	/5Phos/AAAATCATACAGCTCGCGCGGATCTTTAAATGG	Construct *ppglHI::kan*
pglHI-pACYC-F	/5Phos/TTGGTTCCACTCTCTGTTGCGGGCAACTTCAG	Construct *ppglHI::kan*
pglHI-pACYC-R	/5Phos/AGTTGGAACCTCTTACGTGCCGATCAACGTC	Construct *ppglHI::kan*
RT-16S-F	CACACTGGAACTGAGACACG	RT-PCR
RT-16S-R	AGCGTCAGTTGAGTTCCAG	RT-PCR
RT-pglJ-F	GTGTAGGAAGACTTGATAGCGG	RT-PCR
RT-pglJ-R	TCATCACCTAAAAGCTCTCTTGC	RT-PCR
RT-pglY-F	AACCGAGCATGTTAGCCATAC	RT-PCR
RT-pglY-R	TCGTTCATGCTAGGCAAAGC	RT-PCR
RT-pglX-F	TGATTTAGGTACTGGGGATTGTG	RT-PCR
RT-pglX-R	TCTTTTTCAGGCTTACTTGCGTC	RT-PCR
RT-pglB-F	ATGATAGCTGGCTTTCATCAGC	RT-PCR
RT-pglB-R	AACTTGATGGATACCACCAGC	RT-PCR
RT-pglA-F	AGAGAATTTTACGAGGCAGCC	RT-PCR
RT-pglC-R	AGTGAAAATCACCGGACTTCC	RT-PCR

Restriction sites are underlined, ribosomal binding sites introduced by PCR are in italics, start codons are in bold, 5-prime phosphorylation is indicated by /5Phos/.

Table S4. Bacterial strains and plasmids

**Strain or Plasmid**	**Characteristic**	**Source**
E. coli		
DH5α	F-*endA1 hsdR17 supE44 thi-1 recA1* Δ (*argF-lacZYA*) *U169*(80d *lacZ* Δ *M15*) *gyrA96 λ-*	Invitrogen
BL21(λDE3)	F-, *omp*T, *hsd*S_B (r_B-, m_B-), *dcm*, *gal*, (λDE3)	Promega
JM110	*dam dcm supE44 hsdR17 thi leu rpsL1 lacY galK galT ara tonA thr tsx* Δ(*lac-proAB*)/F′ [*traD36 proAB*$^+$*lacI*^{q}*lacZ*ΔM15]	(Sambrook et al., 1989)
CLM24	W3110, *ΔwaaL*	(Feldman et al., 2005)
Campylobacter fetus		
C. fetus subsp. *fetus* ATCC 27374 [NCTC 10842]	Type B, isolated brain of sheep fetus	(Véron and Chatelain, 1973)
C. fetus subsp. *fetus pglX-*	Insertional mutation with kanR in *pglX*, Km^R	This study
C. fetus subsp. *fetus pglJ-*	Insertional mutation with kanR in *pglJ*, Km^R	This study
**Plasmids**		
pPCR-Script Amp SK(+)	Cloning vector	Stratagene
pMW2	pBluescript KS containing *C. jejuni* kanamycin cassette, Amp^R Km^R	(Wösten et al., 2010)
pPCR-Script-*Cffpgl*	PCR product (4868 bps) containing *Cff-pglKXYJ* inserted into *Eco*RV site of plasmid pPCR-Script Amp SK(+), Amp^R	(this study)
pPCR-Script-*CffpglX::kan*	pPCR-Script-Cffpgl with a kanamycin cassette inserted within *pglX* Amp^R, Km^R	(this study)
pPCR-Script-*CffpglY::kan*	pPCR-Script-Cffpgl with a kanamycin cassette inserted within *pglY* Amp^R, Km^R	(this study)
pPCR-Script-*CffpglJ::kan*	pPCR-Script-Cffpgl with a kanamycin cassette inserted within *pglJ* Amp^R, Km^R	(this study)
p*pgl*	Plasmid pACYC184 containing the 16 kb *C. jejuni pgl* locus, Cm^R	(Wacker et al., 2002)
p*pgl-pglH*::*kan*	p*pgl* with a kanamycin cassette inserted within *pglH*, Cm^R, Km^R	(Linton et al., 2005)
p*pgl-pglH*::*kan, cat-*	p*pgl* with a kanamycin cassette inserted within *pglH* and *cat* cassette removed, Km^R	(this study)
p*pgl-pglA*::*kan*	p*pgl* with a kanamycin cassette inserted within *pglA* Cm^R, Km^R	(Linton et al., 2005)

p*pgl-pglA*::*kan, cat-*	p*pgl* with a kanamycin cassette inserted within *pglA* and *cat* cassette removed, Km^R	(this study)
p*pgl-pglJ*::*kan*	p*pgl* with a kanamycin cassette inserted within *pglJ* Cm^R, Km^R	(Linton et al., 2005)
p*pgl-pglJ*::*kan, cat-*	p*pgl* with a kanamycin cassette inserted within *pglJ* and *cat* cassette removed, Km^R	(this study)
p*pgl-pglI*::*kan*	p*pgl* with a kanamycin cassette inserted within *pglI* Cm^R, Km^R	(Linton et al., 2005)
p*pgl-pglI*::*kan, cat-*	p*pgl* with a kanamycin cassette inserted within *pglI* and *cat* cassette removed, Km^R	(this study)
p*pgl-pglHI*::*kan*	p*pgl* with a kanamycin cassette replacing *pglHI*, Km^R	(this study)
pCE111/28	*C. jejuni-E. coli* expression vector with σ^{28} promoter from *flaA,* Cm^R	(Larsen et al., 2004)
pCE111/28 (*Cj-pglA*)	*pglA* of *C. jejuni* cloned into pCE107/70, Cm^R	This study
pCE111/28 (*Cj-pglJ*)	*pglJ* of *C. jejuni* cloned into pCE107/70, Cm^R	This study
pCE111/28 (*Cff-pglA*)	*pglA* of *C. fetus* subsp. *fetus* cloned into pCE107/70, Cm^R	This study
pCE111/28 (*Cff-pglJ*)	*pglJ* of *C. fetus* subsp. *fetus* cloned into pCE107/70, Cm^R	This study
pCE111/28 (*Cj-pglH*)	*pglH* of *C. jejuni* cloned into pCE107/70, Cm^R	This study
pCE111/28 (*Cff-pglX*)	*pglX* of *C. fetus* subsp. *fetus* cloned into pCE107/70, Cm^R	This study
pCE111/28 (*Cff-pglY*)	*pglY* of *C. fetus* subsp. *fetus* cloned into pCE107/70, Cm^R	This study
pCE111/28 (*Cff-pglXY*)	*pglXY* of *C. fetus* subsp. *fetus* cloned into pCE107/70, Cm^R	This study
pCE111/28 (*Cff-pglX*+*pglY*)	*pglX* and *pglY* of *C. fetus* subsp. *fetus* cloned into pCE107/70, Cm^R	This study
pIH18	Expression of CmeA-His_6 from *C. jejuni* in *E. coli,* $Spec^R$	(Hug et al., 2010)

References

Cox, J., and Mann, M. (2008). MaxQuant enables high peptide identification rates, individualized p.p.b.-range mass accuracies and proteome-wide protein quantification. *Nat Biotechnol* 26**,** 1367-1372. doi:10.1038/nbt.1511.

Feldman, M.F., Wacker, M., Hernandez, M., Hitchen, P.G., Marolda, C.L., Kowarik, M., Morris, H.R., Dell, A., Valvano, M.A., and Aebi, M. (2005). Engineering N-linked protein glycosylation with diverse O antigen lipopolysaccharide structures in Escherichia coli. *Proc Natl Acad Sci U S A* 102**,** 3016-3021. doi:10.1073/pnas.0500044102.

Hug, I., Couturier, M.R., Rooker, M.M., Taylor, D.E., Stein, M., and Feldman, M.F. (2010). *Helicobacter pylori* lipopolysaccharide is synthesized via a novel pathway with an evolutionary connection to protein N-glycosylation. *PLoS Pathog* 6**,** e1000819. doi:10.1371/journal.ppat.1000819.

Ishihama, Y., Rappsilber, J., and Mann, M. (2006). Modular stop and go extraction tips with stacked disks for parallel and multidimensional Peptide fractionation in proteomics. *J Proteome Res* 5**,** 988-994. doi:10.1021/pr050385q.

Larsen, J.C., Szymanski, C., and Guerry, P. (2004). N-linked protein glycosylation is required for full competence in *Campylobacter jejuni* 81-176. *J Bacteriol* 186**,** 6508-6514. doi:10.1128/JB.186.19.6508-6514.2004.

Linton, D., Dorrell, N., Hitchen, P.G., Amber, S., Karlyshev, A.V., Morris, H.R., Dell, A., Valvano, M.A., Aebi, M., and Wren, B.W. (2005). Functional analysis of the *Campylobacter jejuni* N-linked protein glycosylation pathway. *Mol Microbiol* 55**,** 1695-1703. doi:10.1111/j.1365-2958.2005.04519.x.

Ramirez, A.S., Boilevin, J., Mehdipour, A.R., Hummer, G., Darbre, T., Reymond, J.L., and Locher, K.P. (2018). Structural basis of the molecular ruler mechanism of a bacterial glycosyltransferase. *Nat Commun* 9**,** 445. doi:10.1038/s41467-018-02880-2.

Rappsilber, J., Mann, M., and Ishihama, Y. (2007). Protocol for micro-purification, enrichment, pre-fractionation and storage of peptides for proteomics using StageTips. *Nat Protoc* 2**,** 1896-1906. doi:10.1038/nprot.2007.261.

Sambrook, J., Fritsch, E.F., and Maniatis, T. (1989). *Molecular cloning: a laboratory manual.* Cold Spring Harbor Laboratory Press, New York. doi:10.1016/0167-7799(91)90068-S.

Shevchenko, A., Tomas, H., Havlis, J., Olsen, J.V., and Mann, M. (2006). In-gel digestion for mass spectrometric characterization of proteins and proteomes. *Nat Protoc* 1**,** 2856-2860. doi:10.1038/nprot.2006.468.

Véron, M., and Chatelain, R. (1973). Taxonomic study of the genus *Campylobacter* Sebald and Véron and designation of the neotype strain for the type species, *Campylobacter fetus* (Smith and Taylor) Sebald and Véron. *Int J Syst Bacteriol* 23**,** 122-134. doi:10.1099/00207713-23-2-122.

Wacker, M., Linton, D., Hitchen, P.G., Nita-Lazar, M., Haslam, S.M., North, S.J., Panico, M., Morris, H.R., Dell, A., Wren, B.W., and Aebi, M. (2002). N-linked glycosylation in

Campylobacter jejuni and its functional transfer into *E. coli*. *Science* 298**,** 1790-1793. doi:10.1126/science.298.5599.1790.

Wösten, M.M.S.M., Van Dijk, L., Veenendaal, A.K.J., De Zoete, M.R., Bleumink-Pluijm, N.M.C., and Van Putten, J.P.M. (2010). Temperature-dependent FlgM/FliA complex formation regulates *Campylobacter jejuni* flagella length. *Mol Microbiol* 75**,** 1577-1591. doi:10.1111/j.1365-2958.2010.07079.x.
