# Supplementary material for: Influence of Protein Glycosylation on Campylobacter fetus Physiology
Source: Front Microbiol. 2020 Jun 17;11:1191. doi: 10.3389/fmicb.2020.01191 (PMC7313396; doi:10.3389/fmicb.2020.01191)
Supplement: Supplementary file 3 [file Data_Sheet_1.PDF]

frontiers

Supplementary MS data 1

Glycopeptides identified from *C. fetus* subsp. *fetus* ATCC 27374

Sample	Protein	m/z	z	Glycan mass	Glycan composition	Peptide mass	Peptide sequence	Mascot Score	Page
ATCC 27374 WT	A0RP41	1093.0034	2	1243.52624	diNAcBac-HexNAc*5	941.4727	FGDFNITK	-	2
ATCC 27374 WT	A0RP41	1072.4865	2	1202.4923	diNAcBac-HexNAc*4-Hex	941.4727	FGDFNITK	28	3 and 4
ATCC 27374 WT	A0RP23	1110.039	2	1243.52314	diNAcBac-HexNAc*5	975.5469	SLKDINGTK	-	5
ATCC 27374 WT	A0RP23	1089.525	2	1202.49512	diNAcBac-HexNAc*4-Hex	975.5469	SLKDINGTK	27	6 and 7
ATCC 27374 WT	A0RP23	992.4863	3	1243.51942	diNAcBac-HexNAc*5	1731.9236	ANNDTIKSLKDINGTK	-	8
ATCC 27374 WT	A0RP23	978.81068	3	1202.49256	diNAcBac-HexNAc*4-Hex	1731.9236	ANNDTIKSLKDINGTK	22	9 and 10
ATCC 27374 *pglX-*	A0RN03	925.96518	2	634.28702	diNAcBac-HexNAc*2	1216.6354	ISDLNLTMPGR	24	11 and 12
ATCC 27374 *pglX-*	A0RP41	788.37733	2	634.27402	diNAcBac-HexNAc*2	941.4727	FGDFNITK	46	13 and 14
ATCC 27374 *pglX-*	A0RP23	789.40857	3	634.28623	diNAcBac-HexNAc*2	1731.9236	ANNDTIKSLKDINGTK	56	15 and 16
ATCC 27374 *pglX-*	A0RP41	887.94371	2	634.27468	diNAcBac-HexNAc*2	1140.6048	AKFGDFNITK	32	17 and 18
ATCC 27374 *pglX-*	A0RP23	703.87244	2	431.19004	diNAcBac-HexNac	975.5469	SLKDINGTK	38	19 and 20
ATCC 27374 *pglX-*	A0RP23	805.42076	2	634.28668	diNAcBac-HexNAc*2	975.5469	SLKDINGTK	46	21 and 22
ATCC 27374 *pglJ-*	A0RP23	805.4191	2	634.28623	diNAcBac-HexNAc*2	975.5469	SLKDINGTK	-	23
ATCC 27374 *pglJ-*	A0RP23	703.87244		432.19004	diNAcBac-HexNAc	975.5469	SLKDINGTK	-	24
ATCC 27374 *pglJ-*	A0RP23	602.33712	2	228.1194	diNAcBac	975.5469	SLKDINGTK	32	25
ATCC 27374 *pglJ-*	A0RP23	789.40857	3	634.28623	diNAcBac-HexNAc*2	1731.9236	ANNDTIKSLKDINGTK	-	26
ATCC 27374 *pglJ-*	A0RP23	722.04317	3	432.19004	diNAcBac-HexNAc	1731.9236	ANNDTIKSLKDINGTK	-	27
ATCC 27374 *pglJ-*	A0RP23	490.76609	4	228.11694	diNAcBac	1731.9236	ANNDTIKSLKDINGTK	26	28

For each glycopeptide derived from either the *Cff* wild-type (WT), the *pglX* mutant (*pglX*-) or the *pglJ* mutant (*pglJ*-), the protein ID, parent mass, glycan mass, glycan composition, peptide mass sequence, and MASCOT score (if available) are shown. All annotated spectra (page numbers are indicated) are included below.

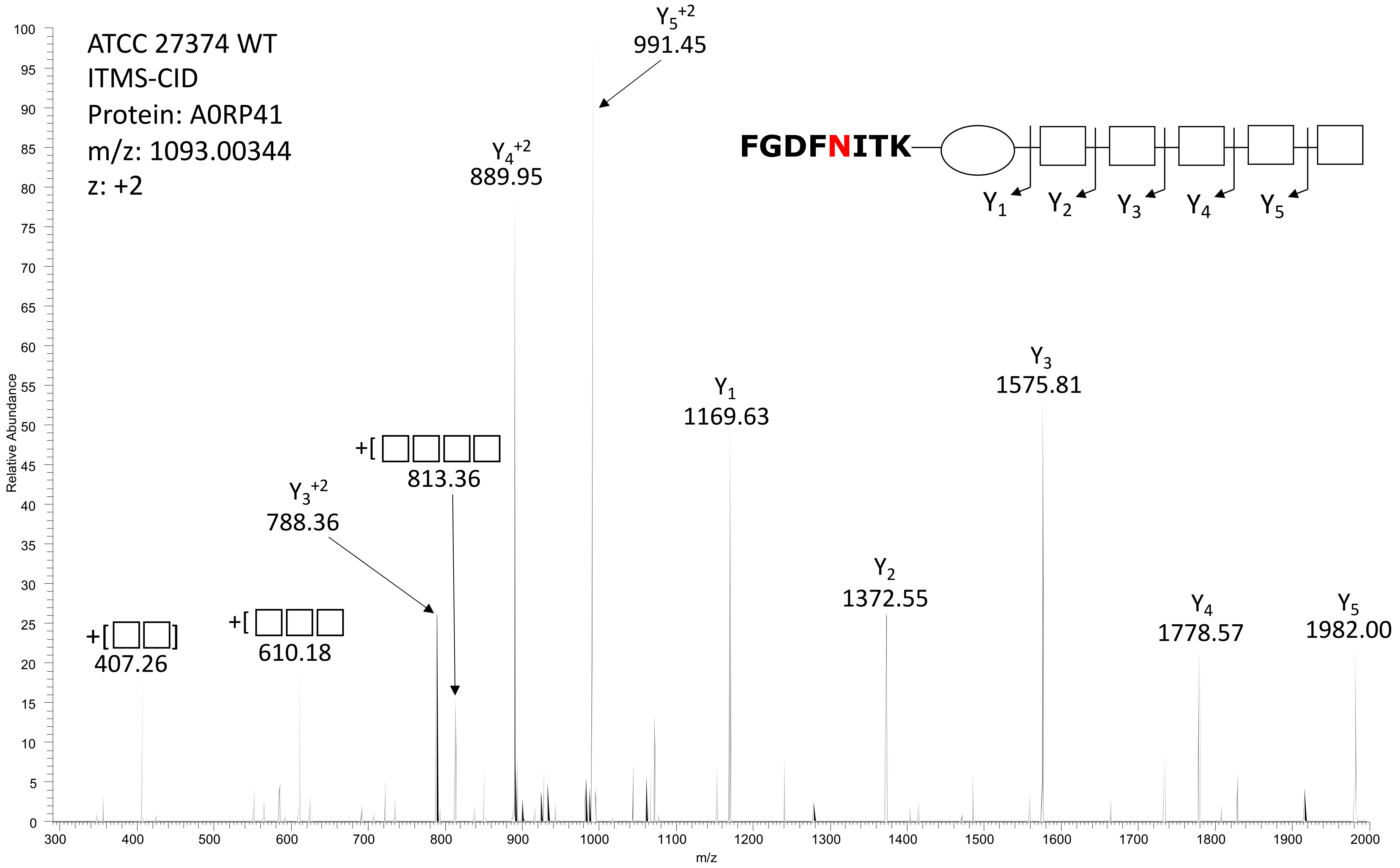
ATCC 27374 WT
ITMS-CID
Protein: A0RP41
m/z: 1093.00344
z: +2
FGDFNITK
Y1 Y2 Y3 Y4 Y5
Y5+2 991.45
Y4+2 889.95
Y1 1169.63
Y3 1575.81
Y2 1372.55
Y4 1778.57
Y5 1982.00
+[813.36
Y3+2 788.36
+[610.18
+[ ] 407.26
Relative Abundance
m/z

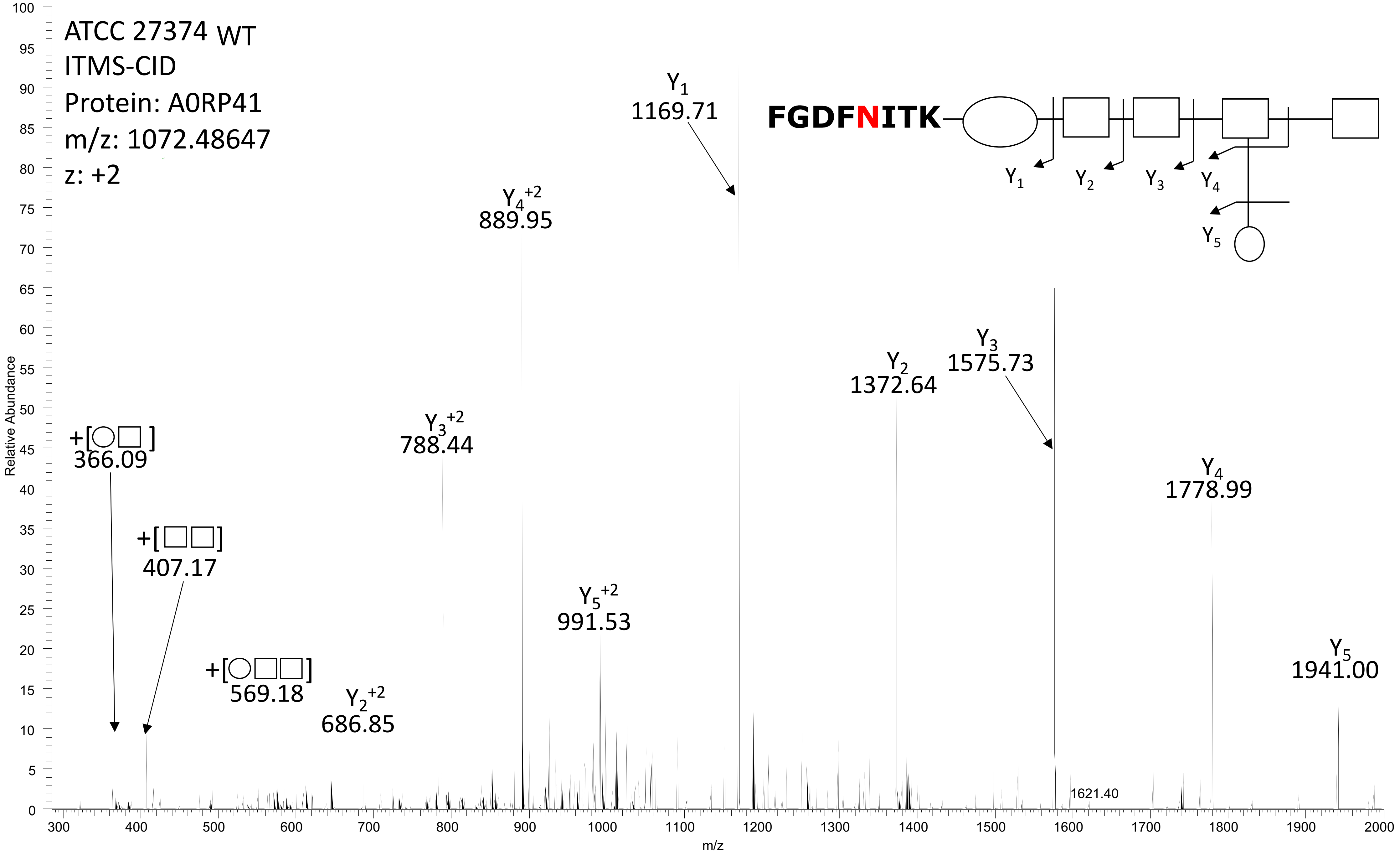
ATCC 27374 WT
ITMS-CID
Protein: A0RP41
m/z: 1072.48647
z: +2
FGDFNITK
Y1
Y2
Y3
Y4
Y5
Y1 1169.71
Y4+2 889.95
Y2 1372.64
Y3 1575.73
Y3+2 788.44
+[○□] 366.09
Y4 1778.99
+[□□] 407.17
Y5+2 991.53
+[○□□] 569.18
Y5 1941.00
Y2+2 686.85
1621.40
Relative Abundance
m/z

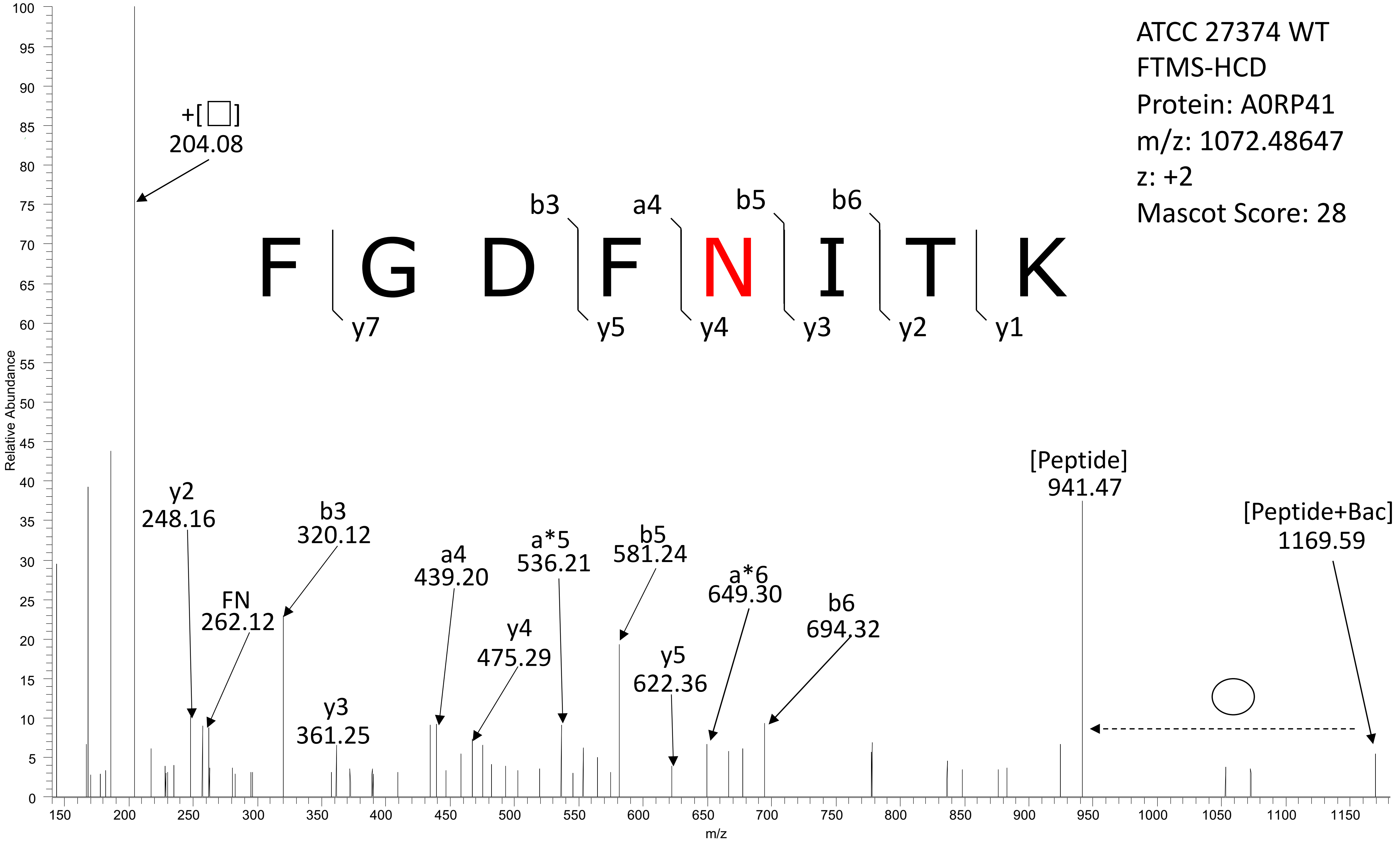

ATCC 27374 WT
FTMS-HCD
Protein: A0RP41
m/z: 1072.48647
z: +2
Mascot Score: 28
F G D F N I T K
b3 a4 b5 b6
y7 y5 y4 y3 y2 y1
+[ ] 204.08
y2 248.16
FN 262.12
b3 320.12
y3 361.25
a4 439.20
y4 475.29
a*5 536.21
b5 581.24
y5 622.36
a*6 649.30
b6 694.32
[Peptide] 941.47
[Peptide+Bac] 1169.59
Relative Abundance
m/z

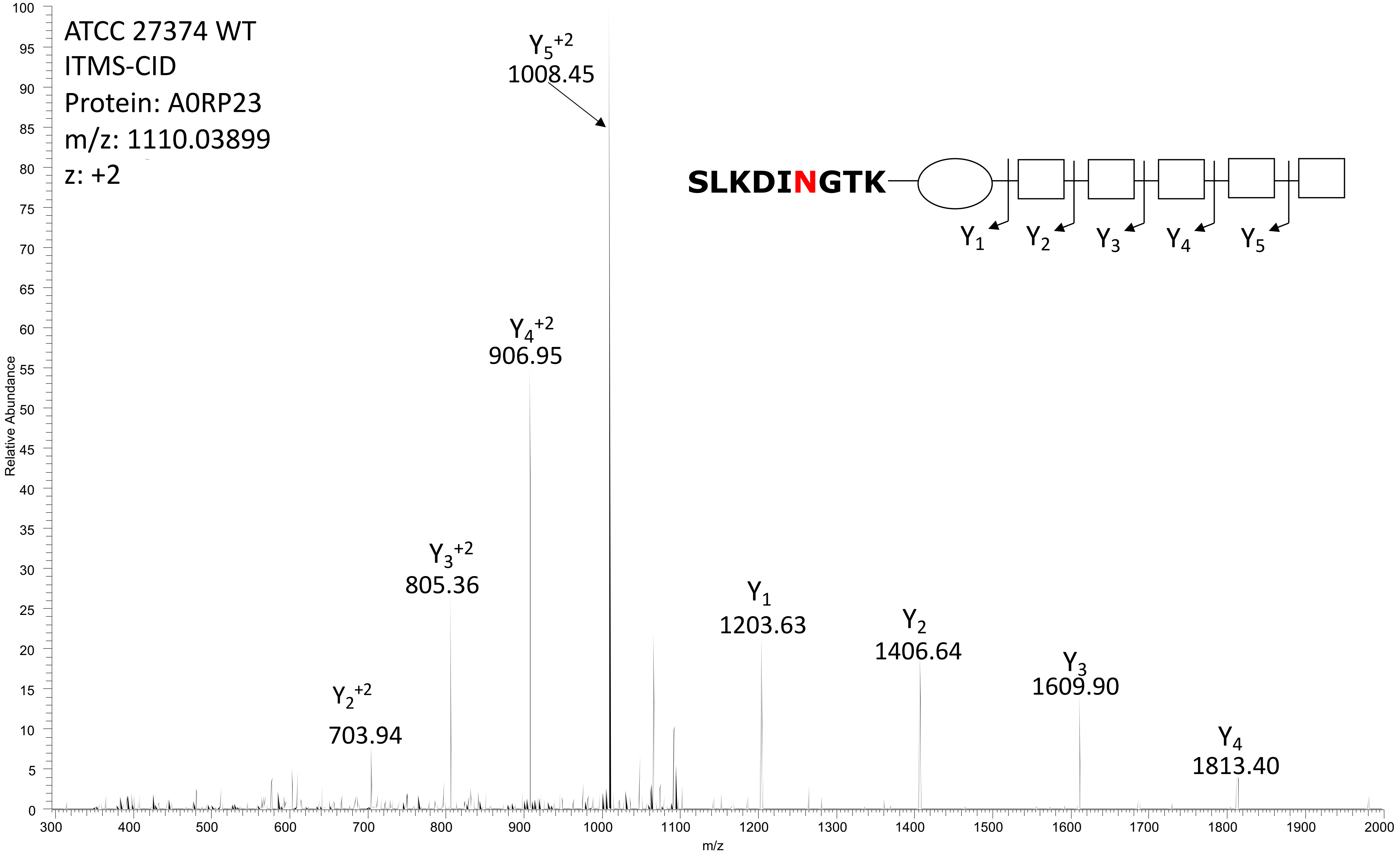

ATCC 27374 WT
ITMS-CID
Protein: A0RP23
m/z: 1110.03899
z: +2
SLKDINGTK
Y1
Y2
Y3
Y4
Y5
Y5+2
1008.45
Y4+2
906.95
Y3+2
805.36
Y2+2
703.94
Y1
1203.63
Y2
1406.64
Y3
1609.90
Y4
1813.40
Relative Abundance
m/z

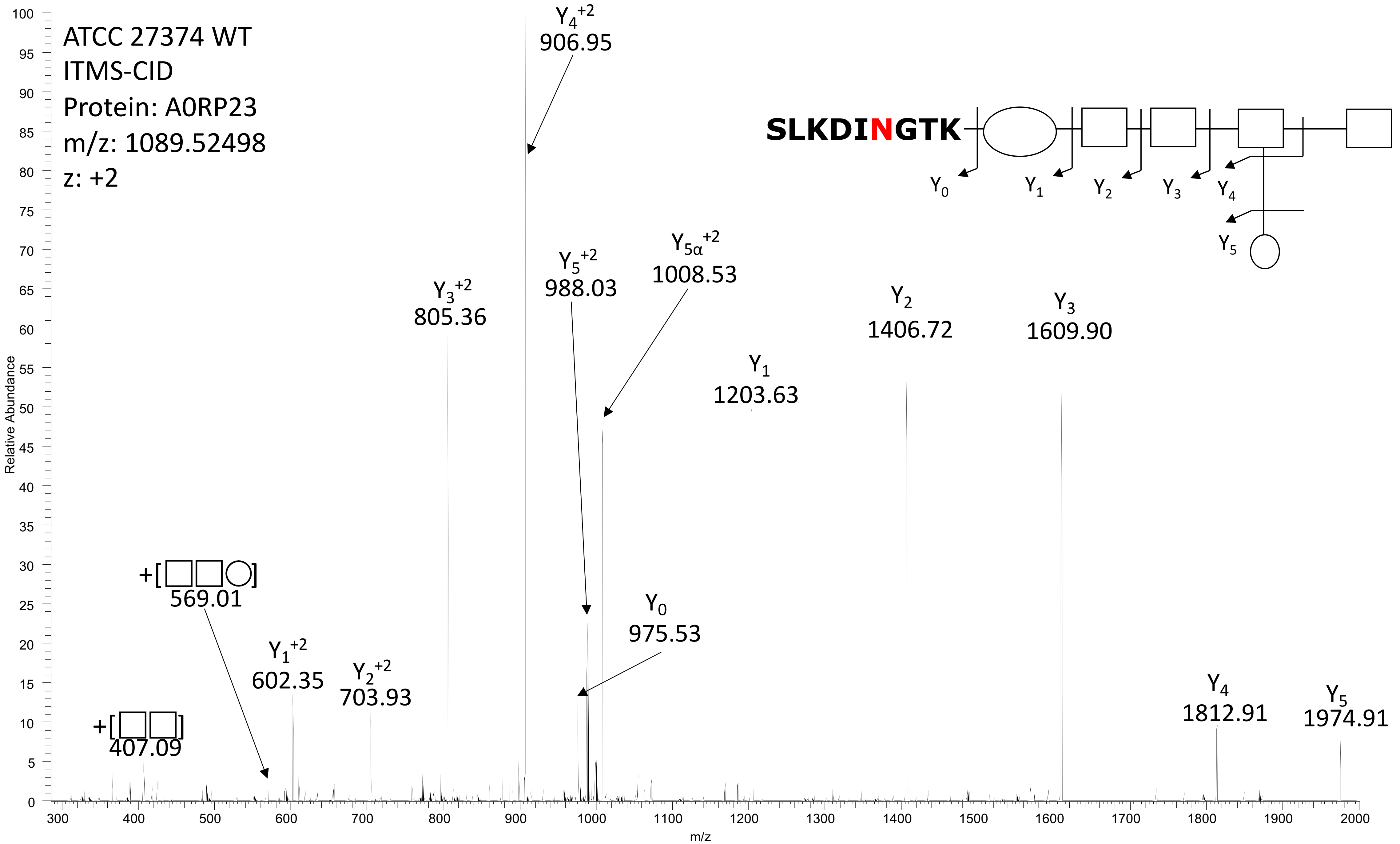

ATCC 27374 WT
ITMS-CID
Protein: A0RP23
m/z: 1089.52498
z: +2
SLKDINGTK
Y0 Y1 Y2 Y3 Y4 Y5
Y4+2 906.95
Y3+2 805.36
Y5+2 988.03
Y5α+2 1008.53
Y2 1406.72
Y3 1609.90
Y1 1203.63
Y0 975.53
+[□□○] 569.01
Y1+2 602.35
Y2+2 703.93
+[□□] 407.09
Y4 1812.91
Y5 1974.91
Relative Abundance
m/z

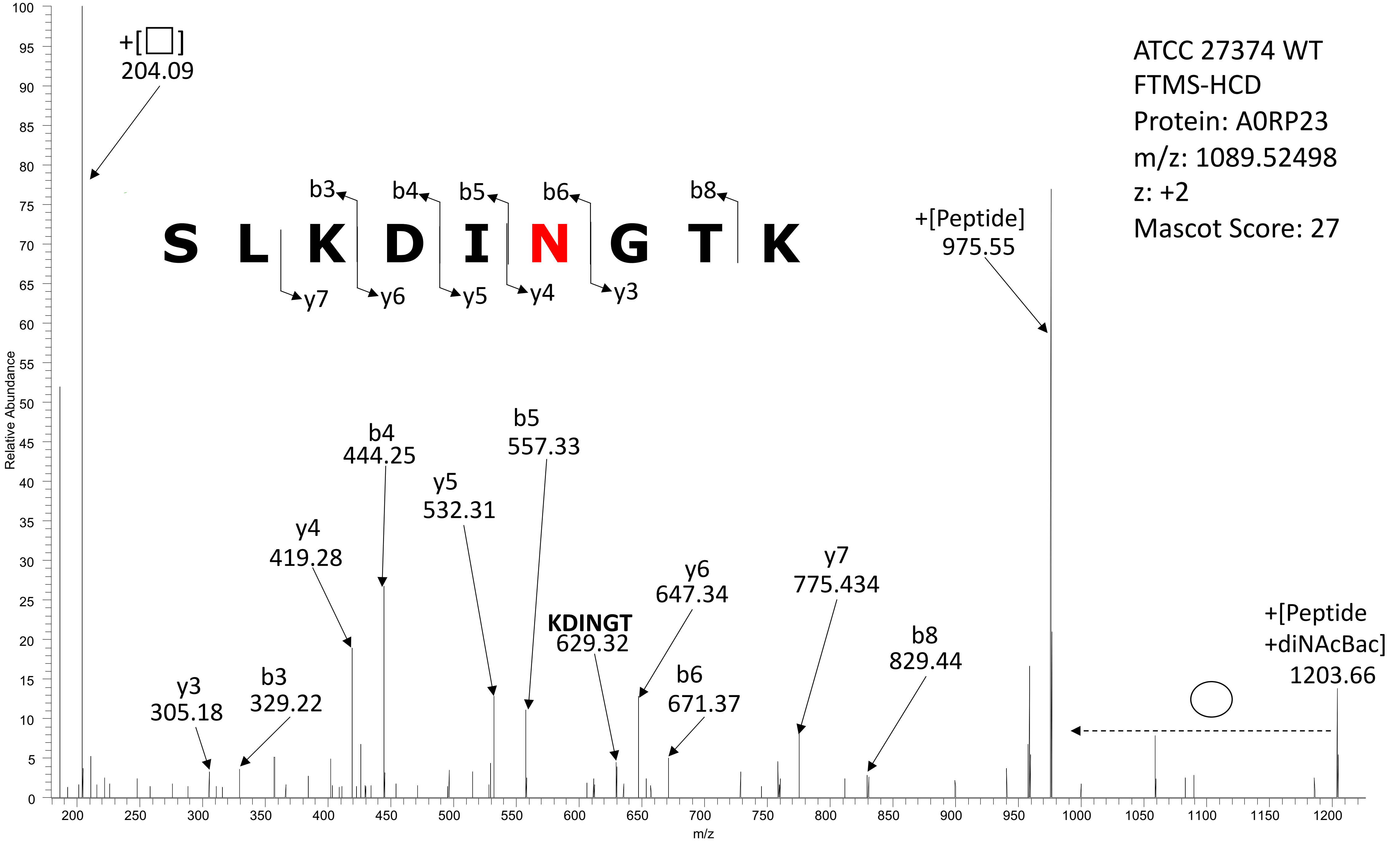

ATCC 27374 WT
FTMS-HCD
Protein: A0RP23
m/z: 1089.52498
z: +2
Mascot Score: 27
S L K D I N G T K
b3 b4 b5 b6 b8
y7 y6 y5 y4 y3
+[ ] 204.09
+[Peptide] 975.55
+[Peptide +diNAcBac] 1203.66
y3 305.18
b3 329.22
y4 419.28
b4 444.25
y5 532.31
b5 557.33
KDINGT 629.32
y6 647.34
b6 671.37
y7 775.434
b8 829.44
Relative Abundance
m/z

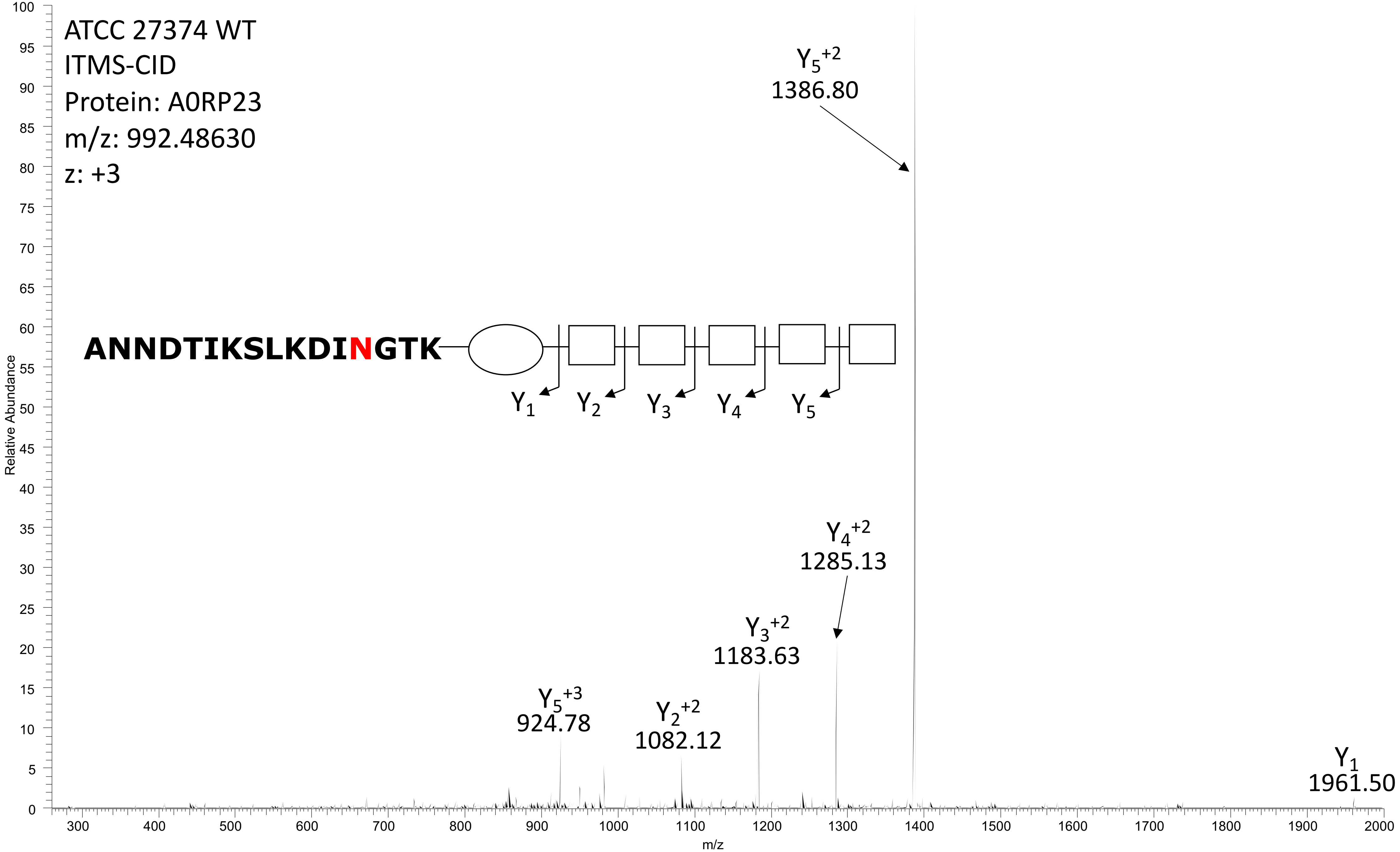
ATCC 27374 WT
ITMS-CID
Protein: A0RP23
m/z: 992.48630
z: +3
ANNDTIKSLKDINGTK
Y1
Y2
Y3
Y4
Y5
Y5+2
1386.80
Y4+2
1285.13
Y3+2
1183.63
Y2+2
1082.12
Y5+3
924.78
Y1
1961.50
Relative Abundance
m/z

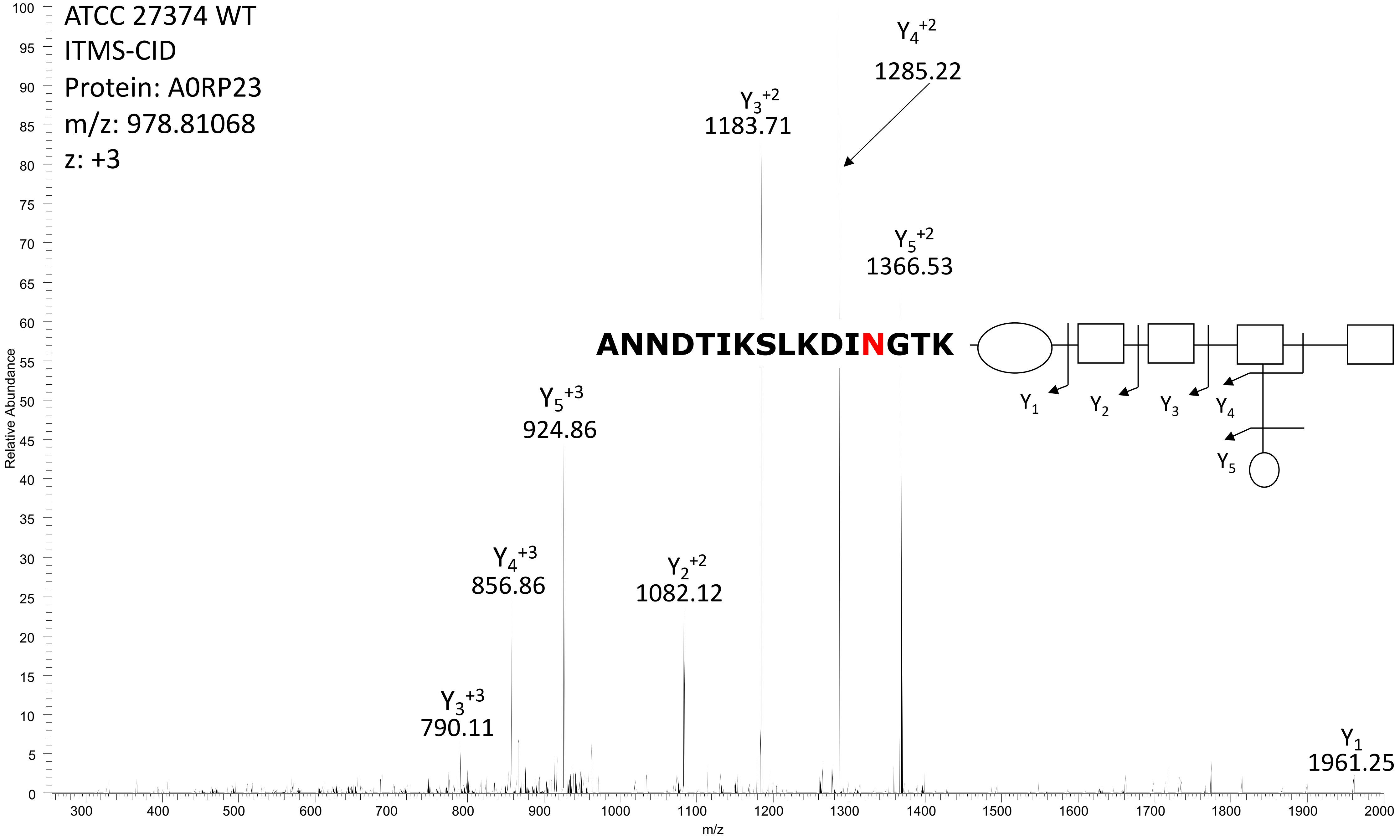
ATCC 27374 WT
ITMS-CID
Protein: A0RP23
m/z: 978.81068
z: +3
Y4+2 1285.22
Y3+2 1183.71
Y5+2 1366.53
ANNDTIKSLKDINGTK
Y1
Y2
Y3
Y4
Y5
Y5+3 924.86
Y4+3 856.86
Y2+2 1082.12
Y3+3 790.11
Y1 1961.25
Relative Abundance
m/z

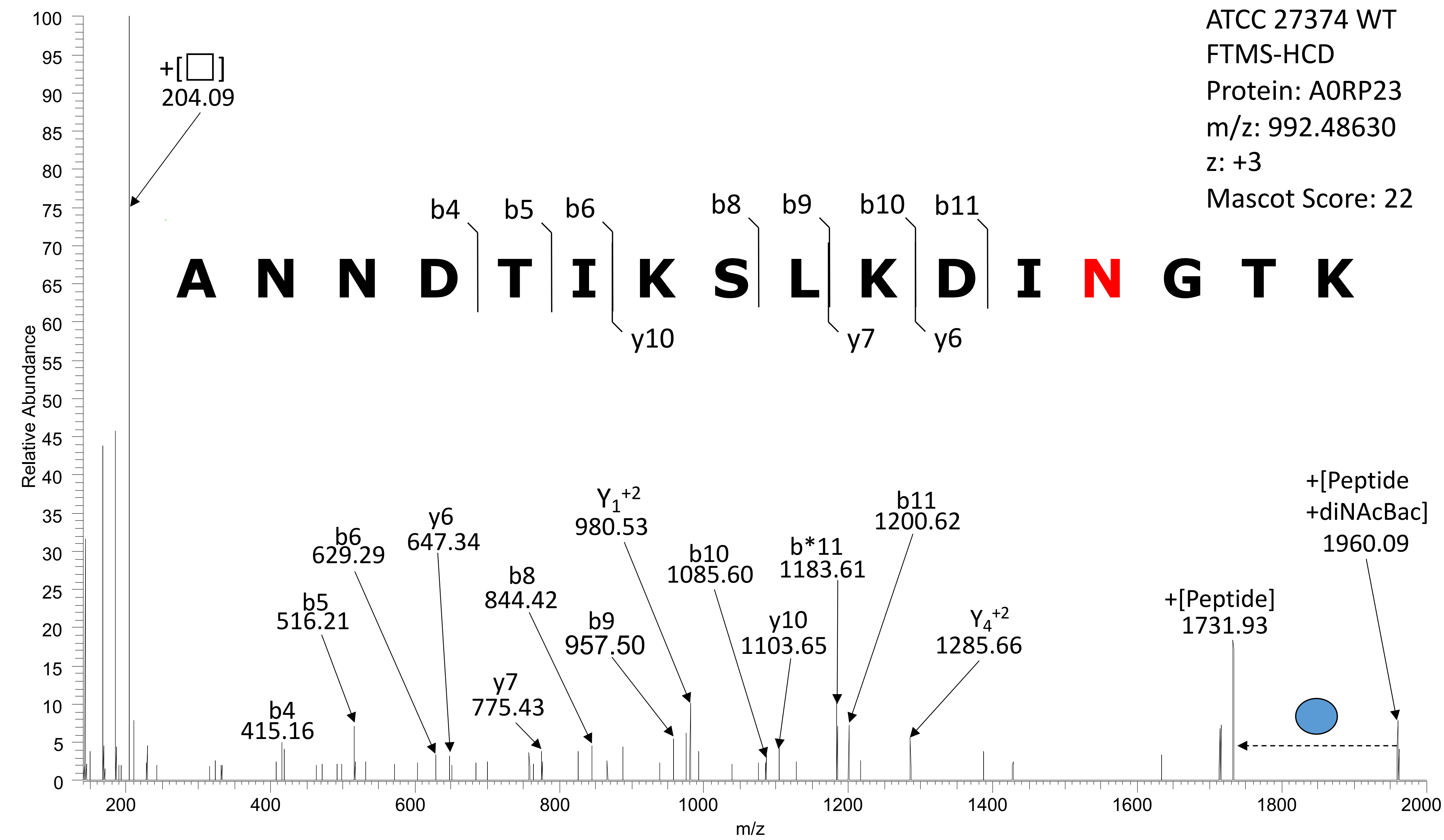
ATCC 27374 WT
FTMS-HCD
Protein: A0RP23
m/z: 992.48630
z: +3
Mascot Score: 22
A N N D T I K S L K D I N G T K
b4 b5 b6 b8 b9 b10 b11
y10 y7 y6
+[□] 204.09
b4 415.16
b5 516.21
b6 629.29
y6 647.34
y7 775.43
b8 844.42
b9 957.50
Y1+2 980.53
b10 1085.60
y10 1103.65
b*11 1183.61
b11 1200.62
Y4+2 1285.66
+[Peptide] 1731.93
+[Peptide +diNAcBac] 1960.09
Relative Abundance
m/z
0 5 10 15 20 25 30 35 40 45 50 55 60 65 70 75 80 85 90 95 100
200 400 600 800 1000 1200 1400 1600 1800 2000

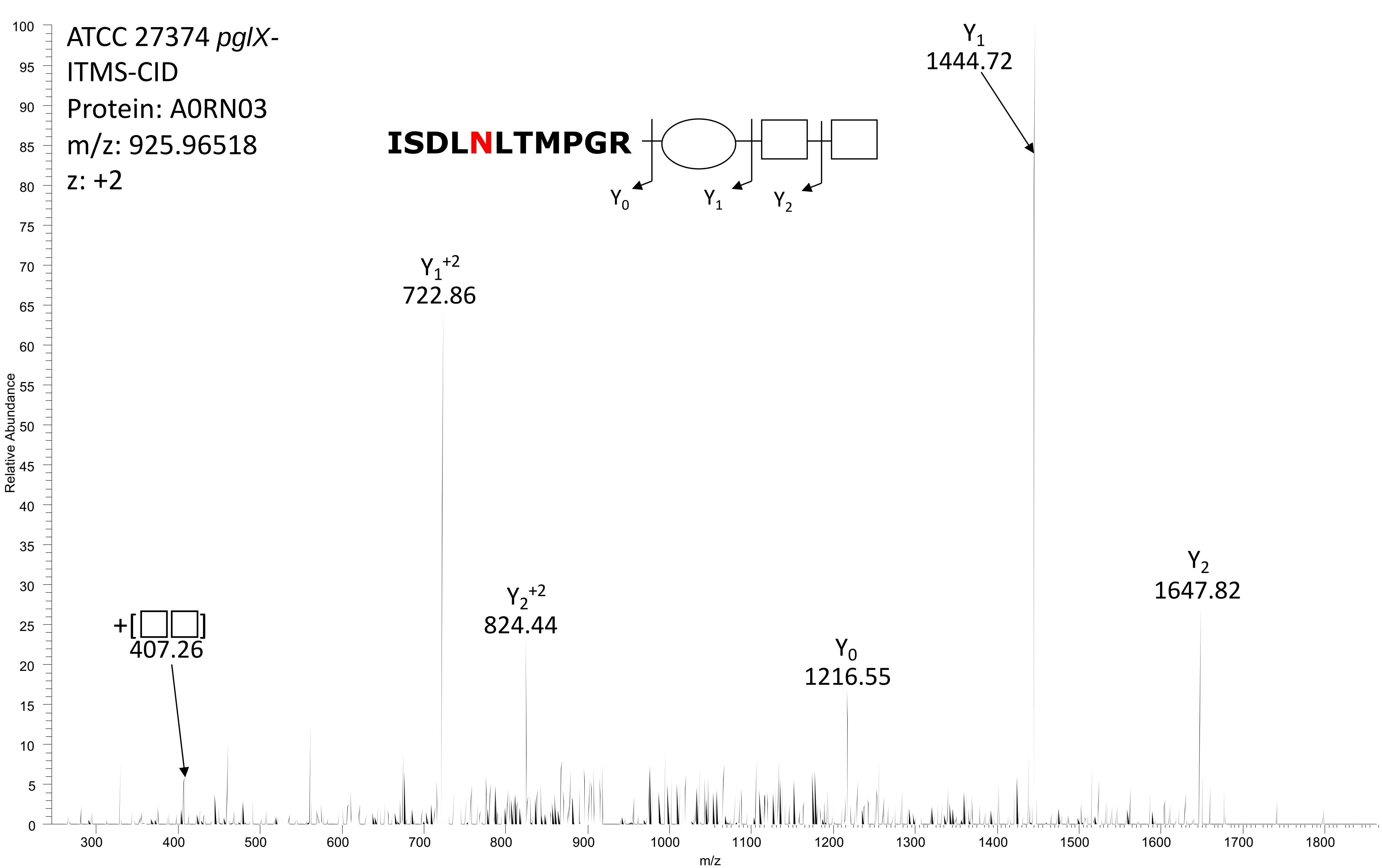
ATCC 27374 pglX-
ITMS-CID
Protein: A0RN03
m/z: 925.96518
z: +2
ISDLNLTMPGR
Y0
Y1
Y2
Y1
1444.72
Y1+2
722.86
Y2+2
824.44
+[□□]
407.26
Y0
1216.55
Y2
1647.82
Relative Abundance
m/z
0
5
10
15
20
25
30
35
40
45
50
55
60
65
70
75
80
85
90
95
100
300
400
500
600
700
800
900
1000
1100
1200
1300
1400
1500
1600
1700
1800

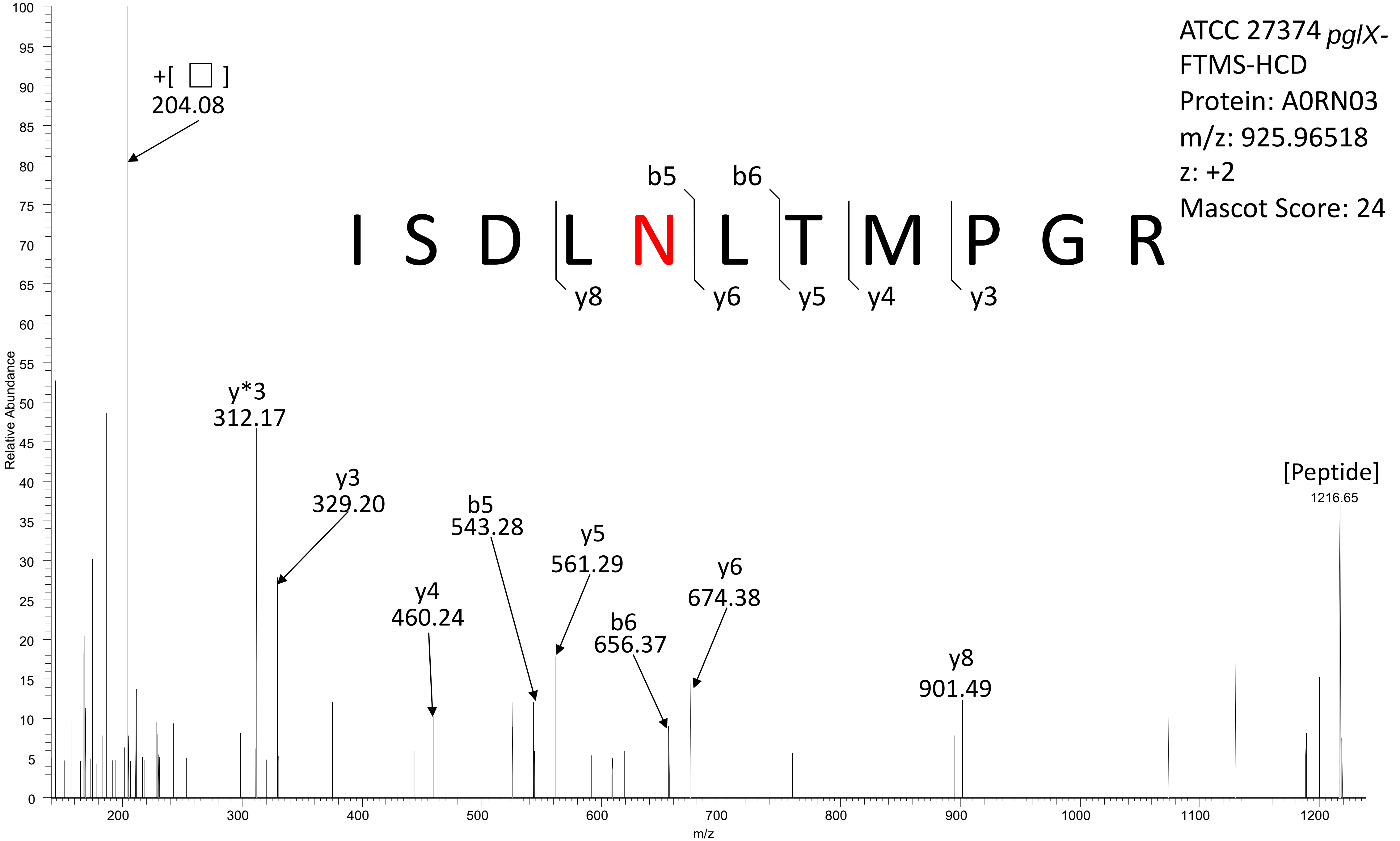
ATCC 27374 pglX-
FTMS-HCD
Protein: A0RN03
m/z: 925.96518
z: +2
Mascot Score: 24
I S D L N L T M P G R
b5 b6
y8 y6 y5 y4 y3
+[ ]
204.08
y*3
312.17
y3
329.20
y4
460.24
b5
543.28
y5
561.29
b6
656.37
y6
674.38
y8
901.49
[Peptide]
1216.65
Relative Abundance
m/z

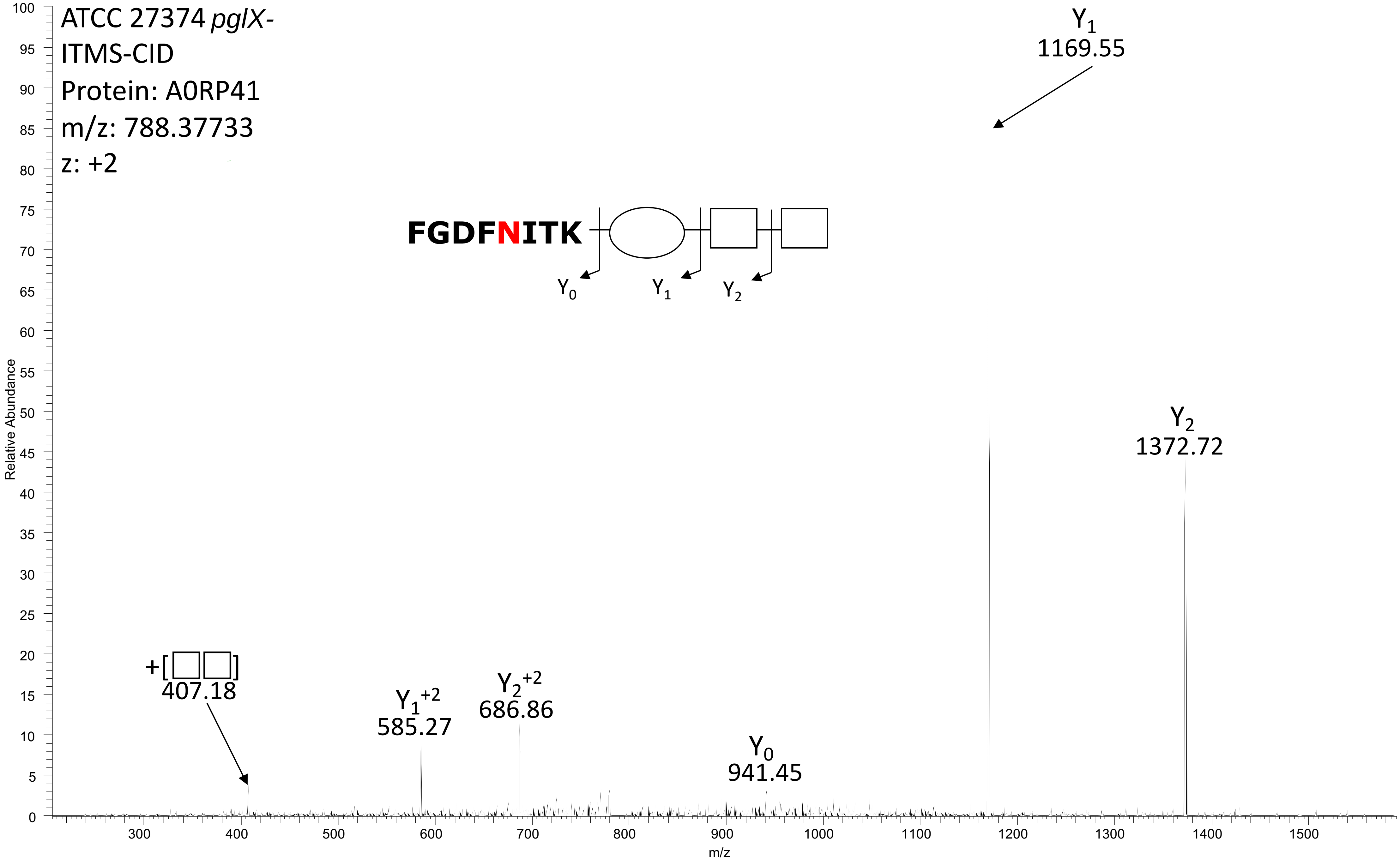

ATCC 27374 *pglX-*
ITMS-CID
Protein: A0RP41
m/z: 788.37733
z: +2
FGDFNITK
Y_0
Y_1
Y_2
Y_1 1169.55
Y_2 1372.72
+[□□] 407.18
Y_1^{+2} 585.27
Y_2^{+2} 686.86
Y_0 941.45
Relative Abundance
m/z

ATCC 27374 *pglX*-
ITMS-CID
Protein: A0RP41
m/z: 788.37733
z: +2
Mascot Score: 46

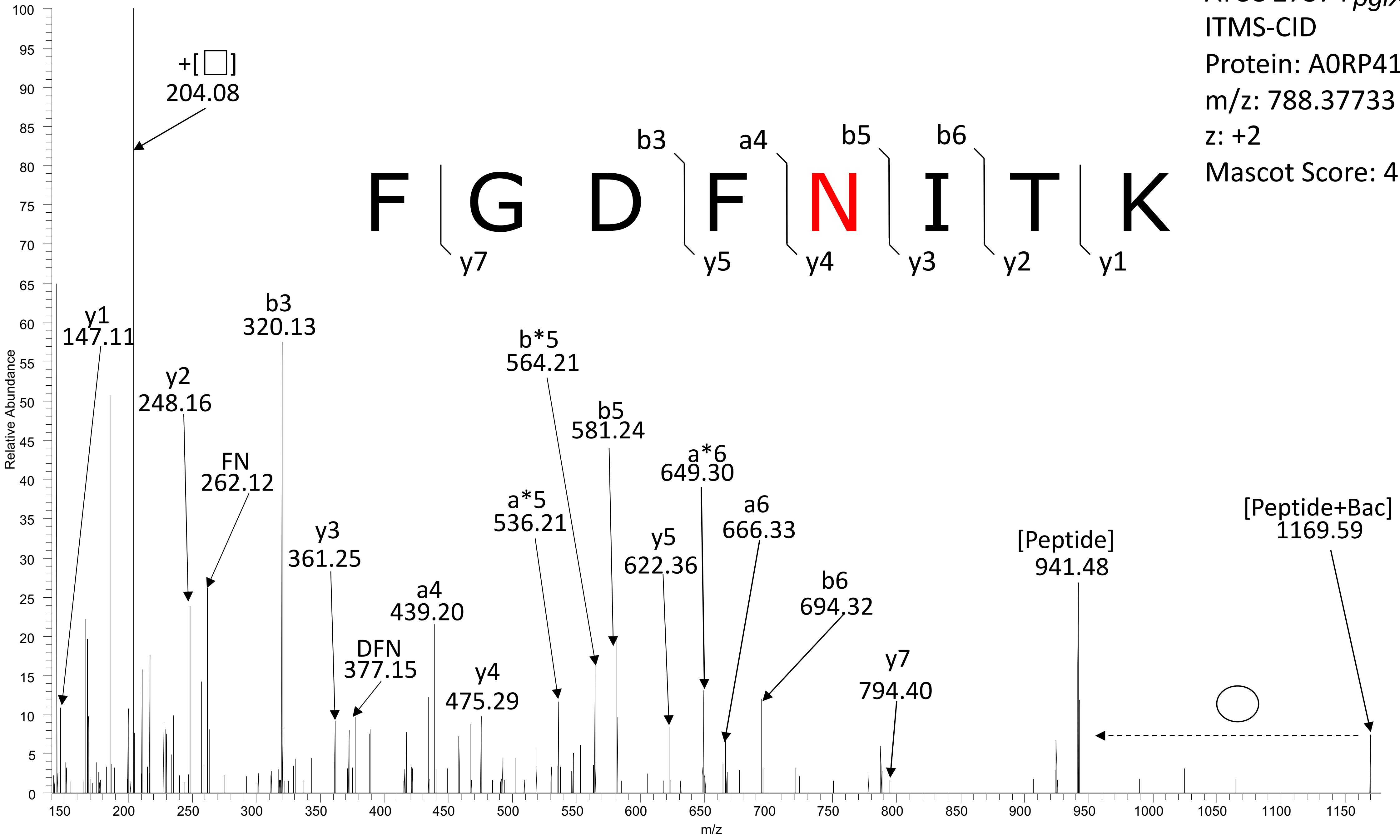

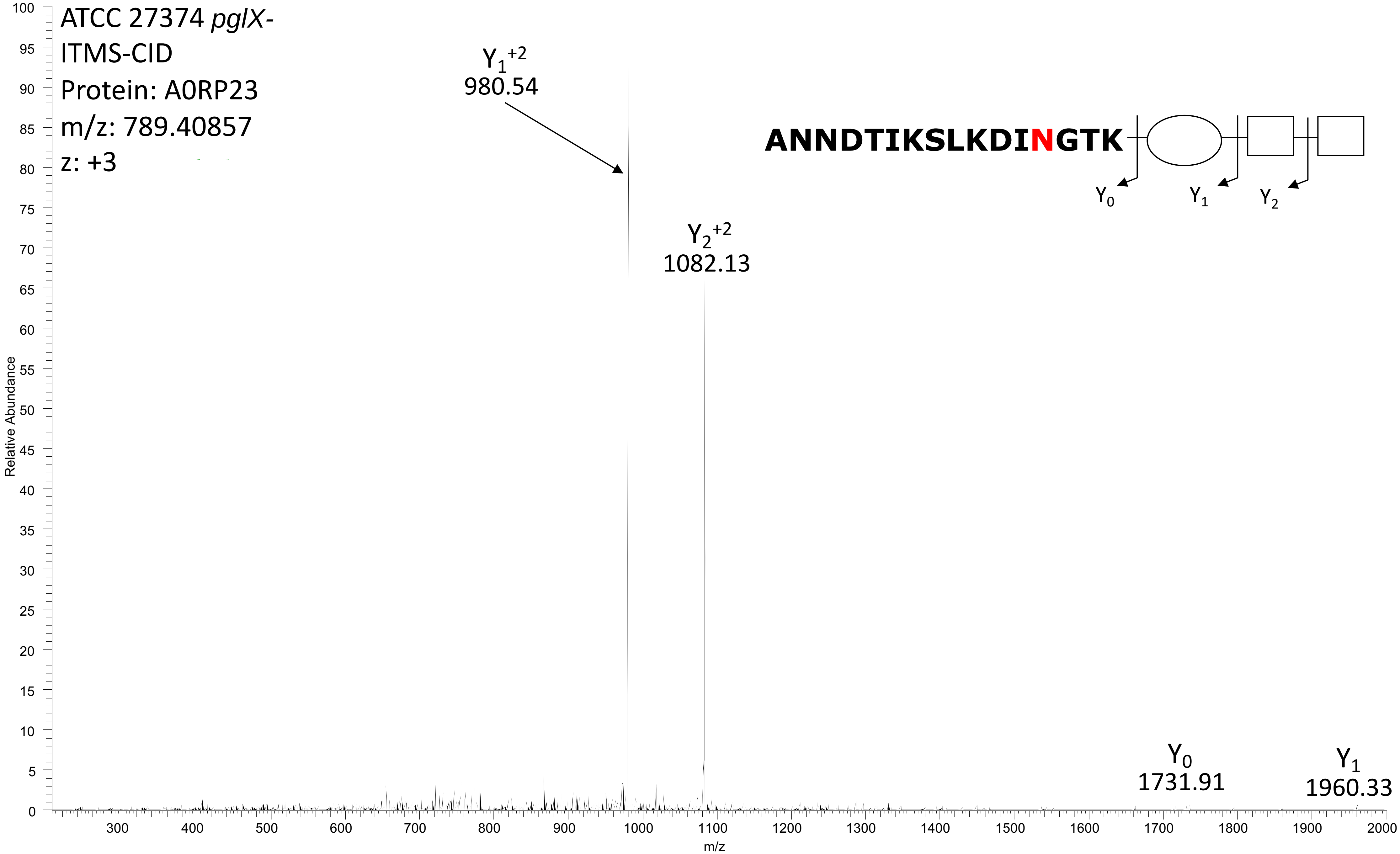
ATCC 27374 pglX-
ITMS-CID
Protein: A0RP23
m/z: 789.40857
z: +3
ANNDTIKSLKDINGTK
Y0
Y1
Y2
Y1+2
980.54
Y2+2
1082.13
Y0
1731.91
Y1
1960.33
Relative Abundance
m/z

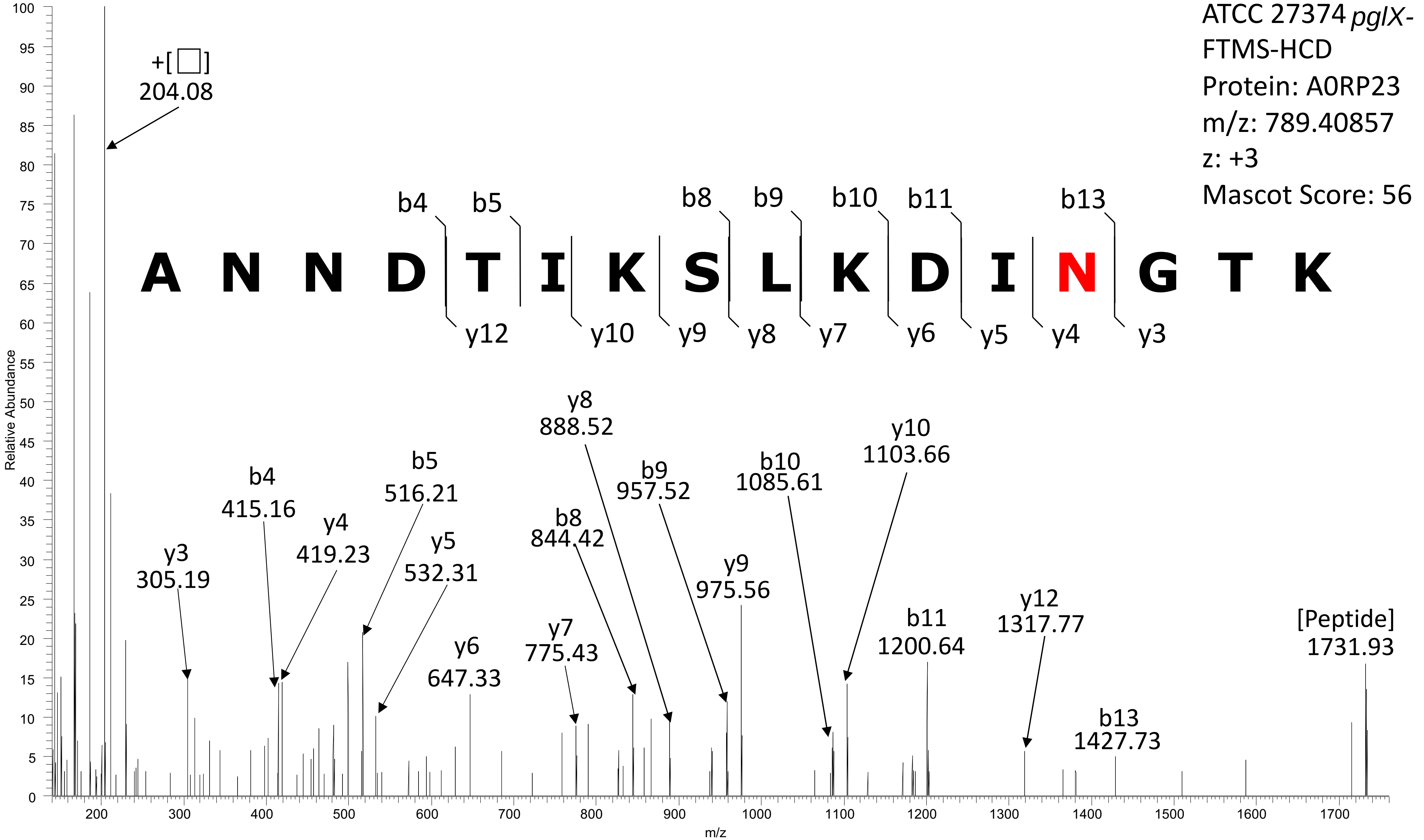
ATCC 27374 pglX-
FTMS-HCD
Protein: A0RP23
m/z: 789.40857
z: +3
Mascot Score: 56
A N N D T I K S L K D I N G T K
b4 b5 b8 b9 b10 b11 b13
y12 y10 y9 y8 y7 y6 y5 y4 y3
+[□] 204.08
y3 305.19
b4 415.16
y4 419.23
b5 516.21
y5 532.31
y6 647.33
y7 775.43
b8 844.42
y8 888.52
b9 957.52
y9 975.56
b10 1085.61
y10 1103.66
b11 1200.64
y12 1317.77
b13 1427.73
[Peptide] 1731.93
Relative Abundance
m/z

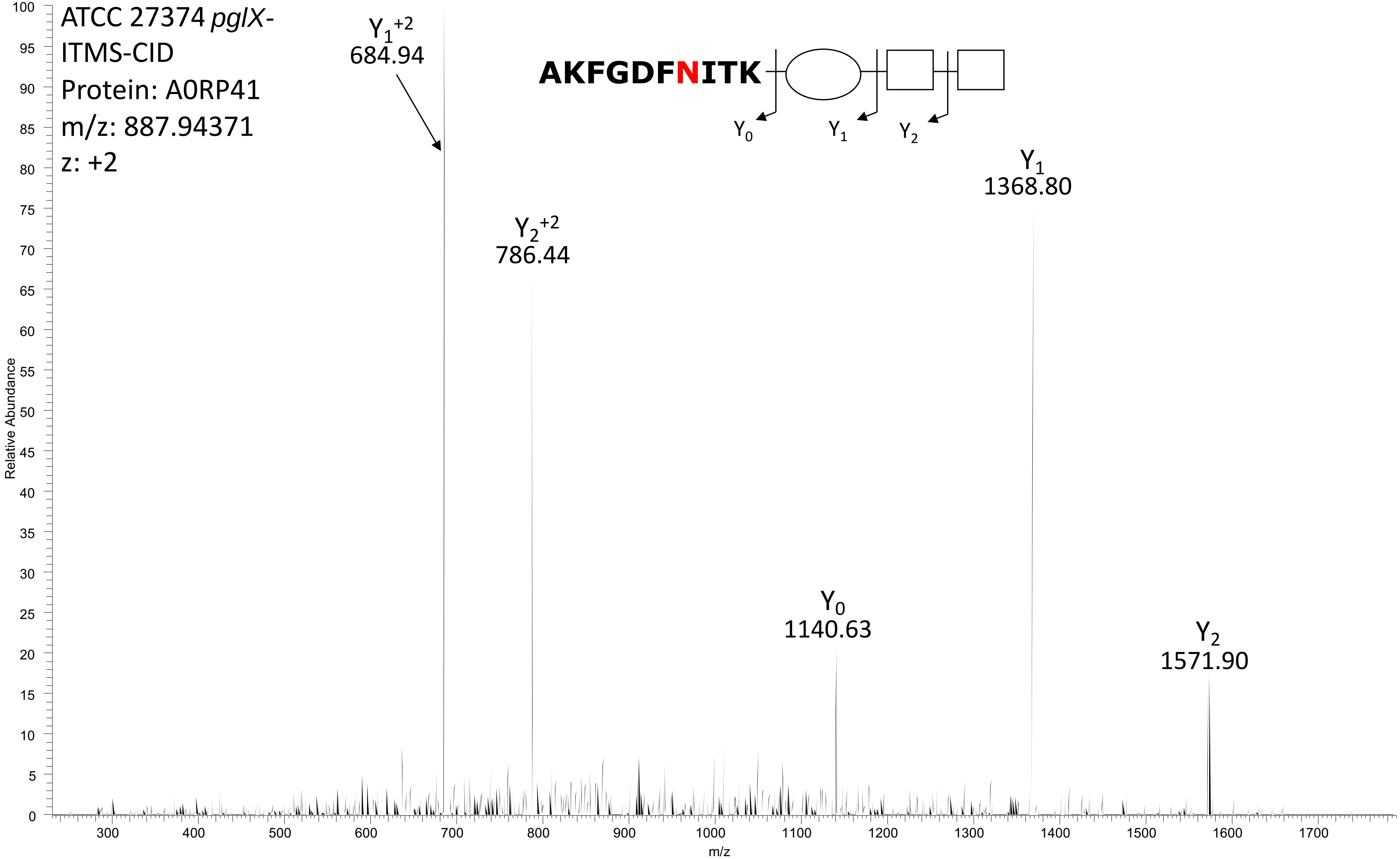
ATCC 27374 *pglX-*
ITMS-CID
Protein: A0RP41
m/z: 887.94371
z: +2
AKFGDFNITK
Y_0
Y_1
Y_2
Y_1^{+2} 684.94
Y_2^{+2} 786.44
Y_0 1140.63
Y_1 1368.80
Y_2 1571.90
Relative Abundance
m/z

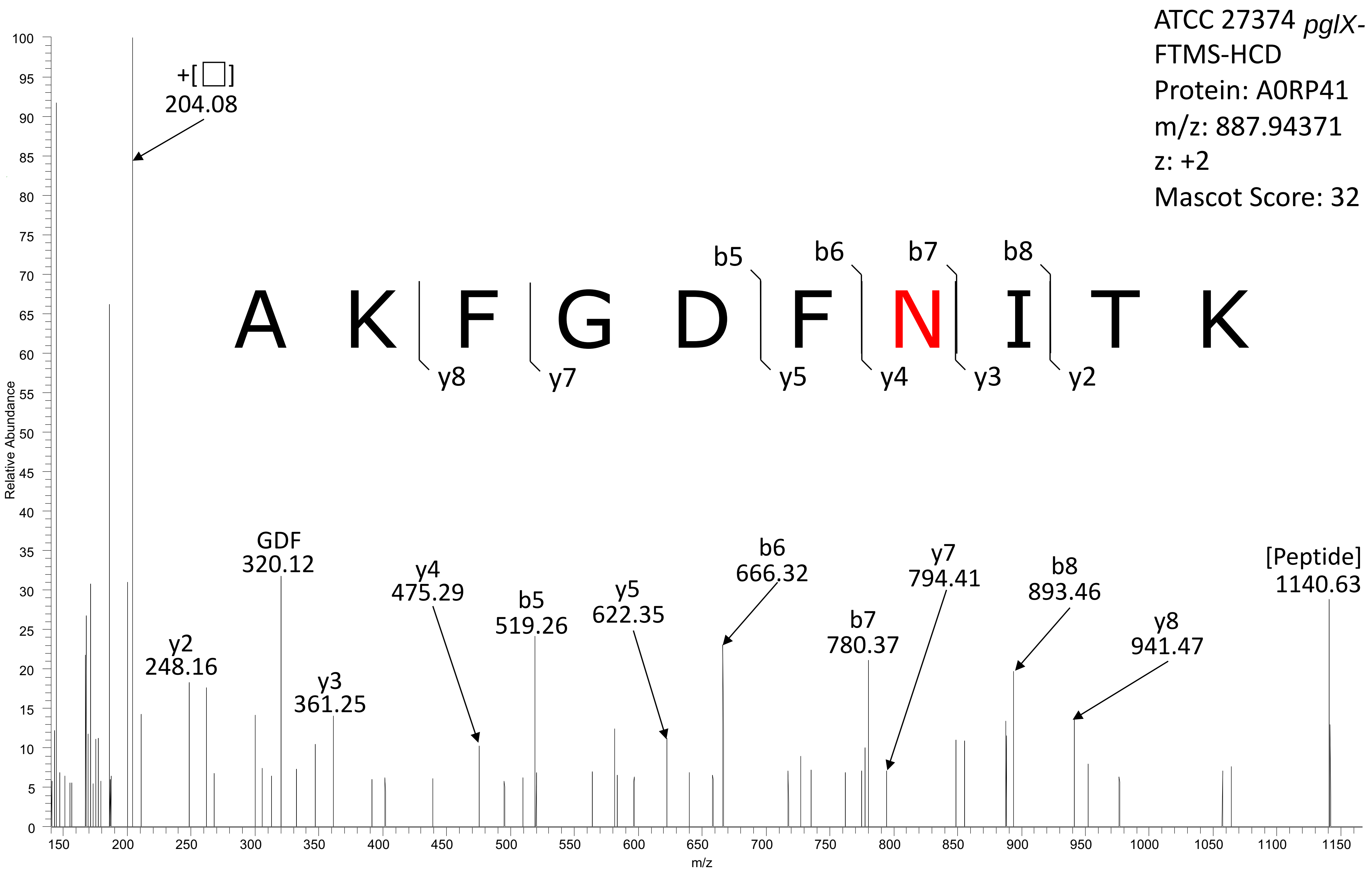
ATCC 27374 pglX-
FTMS-HCD
Protein: A0RP41
m/z: 887.94371
z: +2
Mascot Score: 32
A K F G D F N I T K
b5 b6 b7 b8
y8 y7 y5 y4 y3 y2
+[ ] 204.08
GDF 320.12
y2 248.16
y3 361.25
y4 475.29
b5 519.26
y5 622.35
b6 666.32
b7 780.37
y7 794.41
b8 893.46
y8 941.47
[Peptide] 1140.63
Relative Abundance
m/z

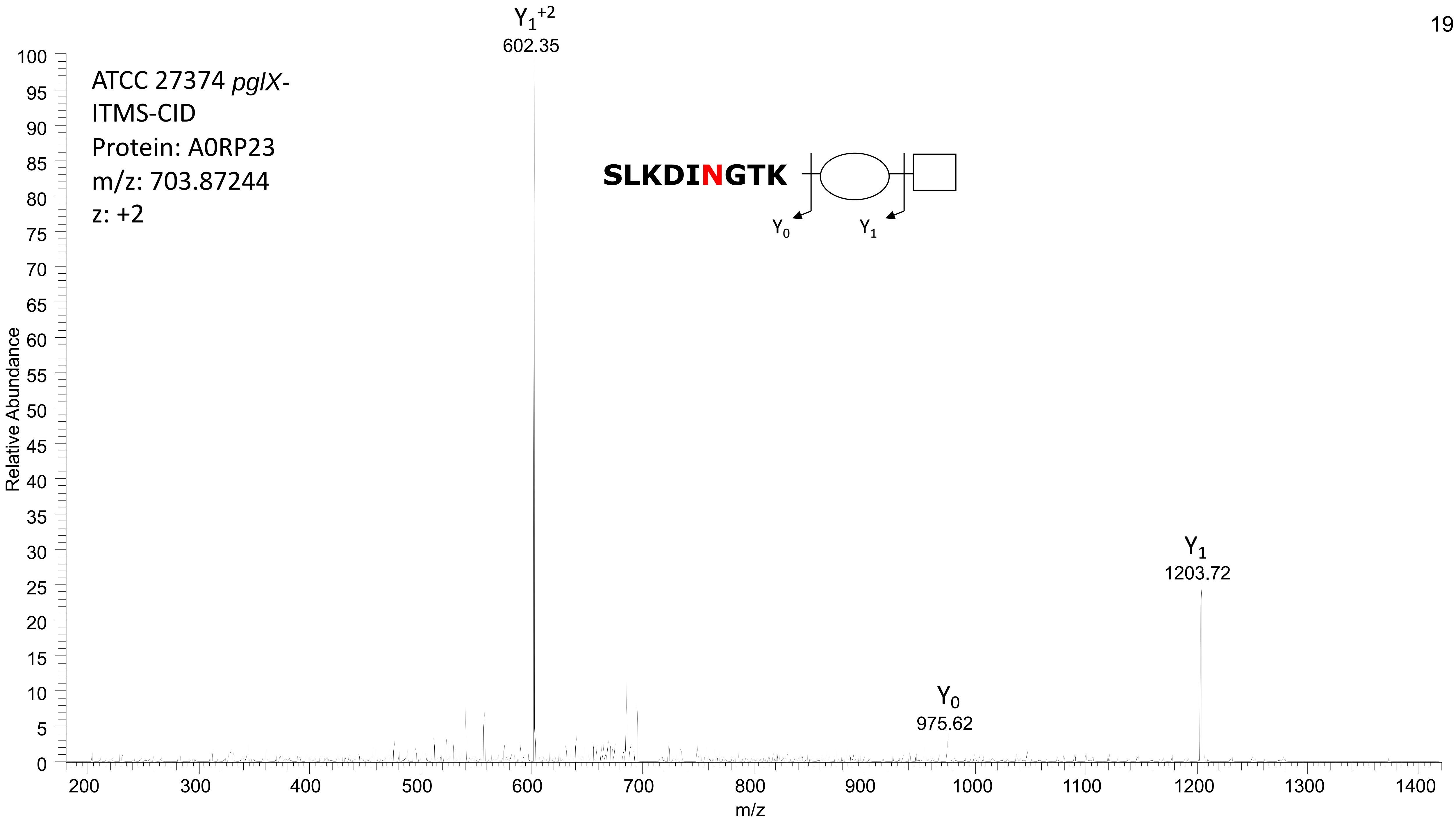
ATCC 27374 pglX-
ITMS-CID
Protein: A0RP23
m/z: 703.87244
z: +2
SLKDINGTK
Y0
Y1
Y1+2
602.35
Y1
1203.72
Y0
975.62
Relative Abundance
m/z
0 5 10 15 20 25 30 35 40 45 50 55 60 65 70 75 80 85 90 95 100
200 300 400 500 600 700 800 900 1000 1100 1200 1300 1400

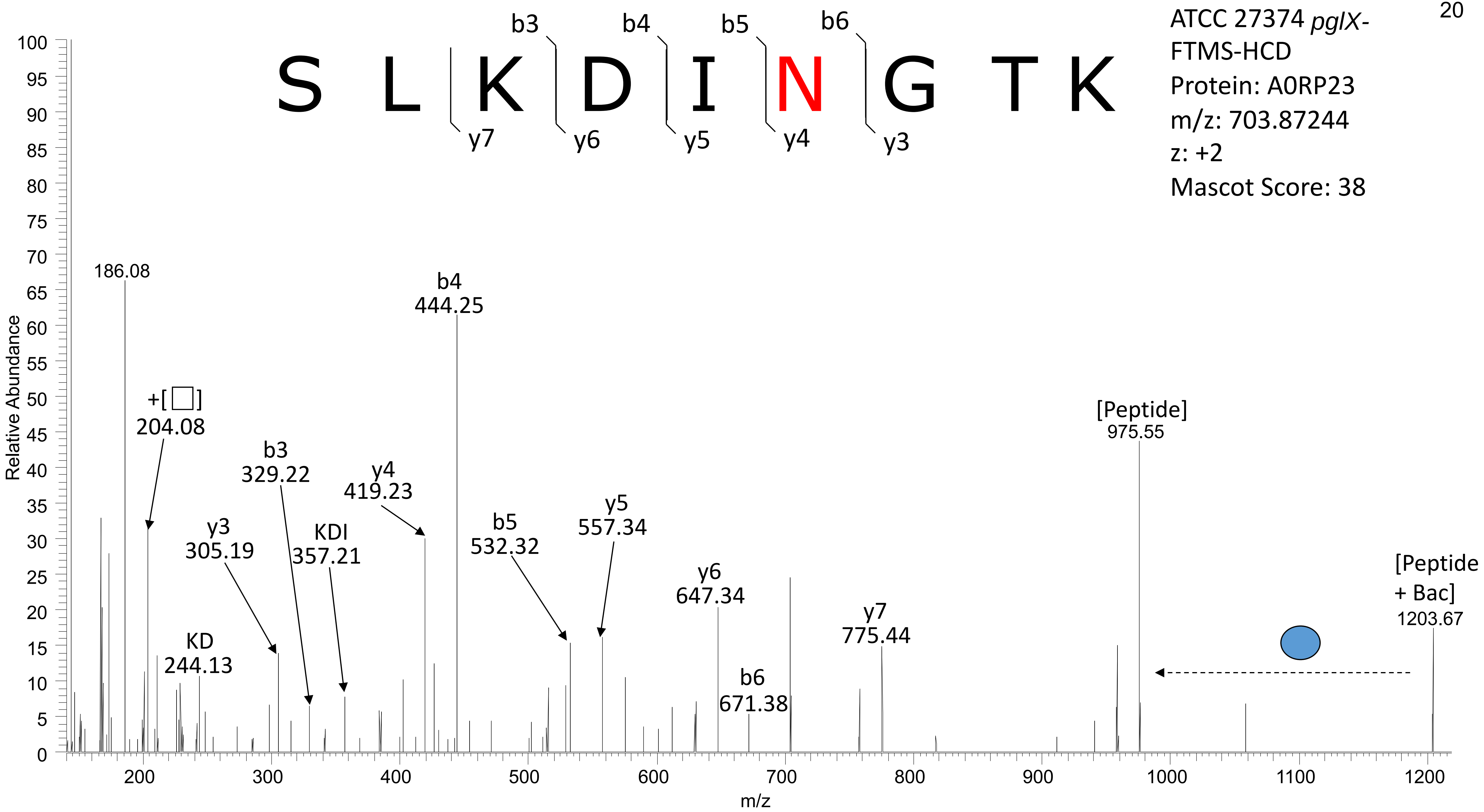

ATCC 27374 *pglX*-
FTMS-HCD
Protein: A0RP23
m/z: 703.87244
z: +2
Mascot Score: 38
S L K D I N G T K
b3 b4 b5 b6
y7 y6 y5 y4 y3
Relative Abundance
m/z
186.08
+[ ]
204.08
KD
244.13
y3
305.19
b3
329.22
KDI
357.21
y4
419.23
b4
444.25
b5
532.32
y5
557.34
y6
647.34
b6
671.38
y7
775.44
[Peptide]
975.55
[Peptide
+ Bac]
1203.67

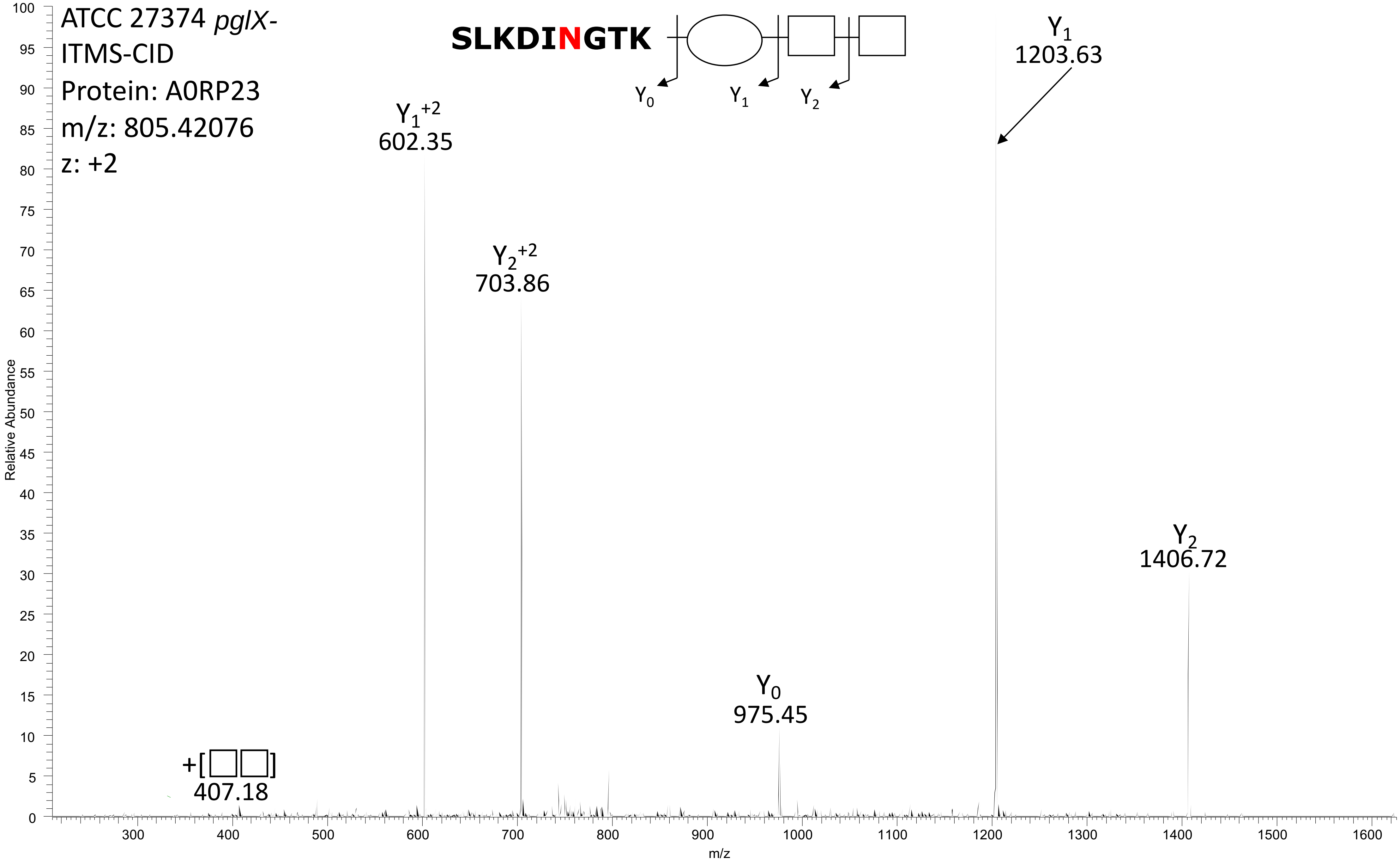
ATCC 27374 *pglX*-
ITMS-CID
Protein: A0RP23
m/z: 805.42076
z: +2
SLKDINGTK
Y0
Y1
Y2
Y1+2
602.35
Y2+2
703.86
Y0
975.45
Y1
1203.63
Y2
1406.72
+[□□]
407.18
Relative Abundance
m/z

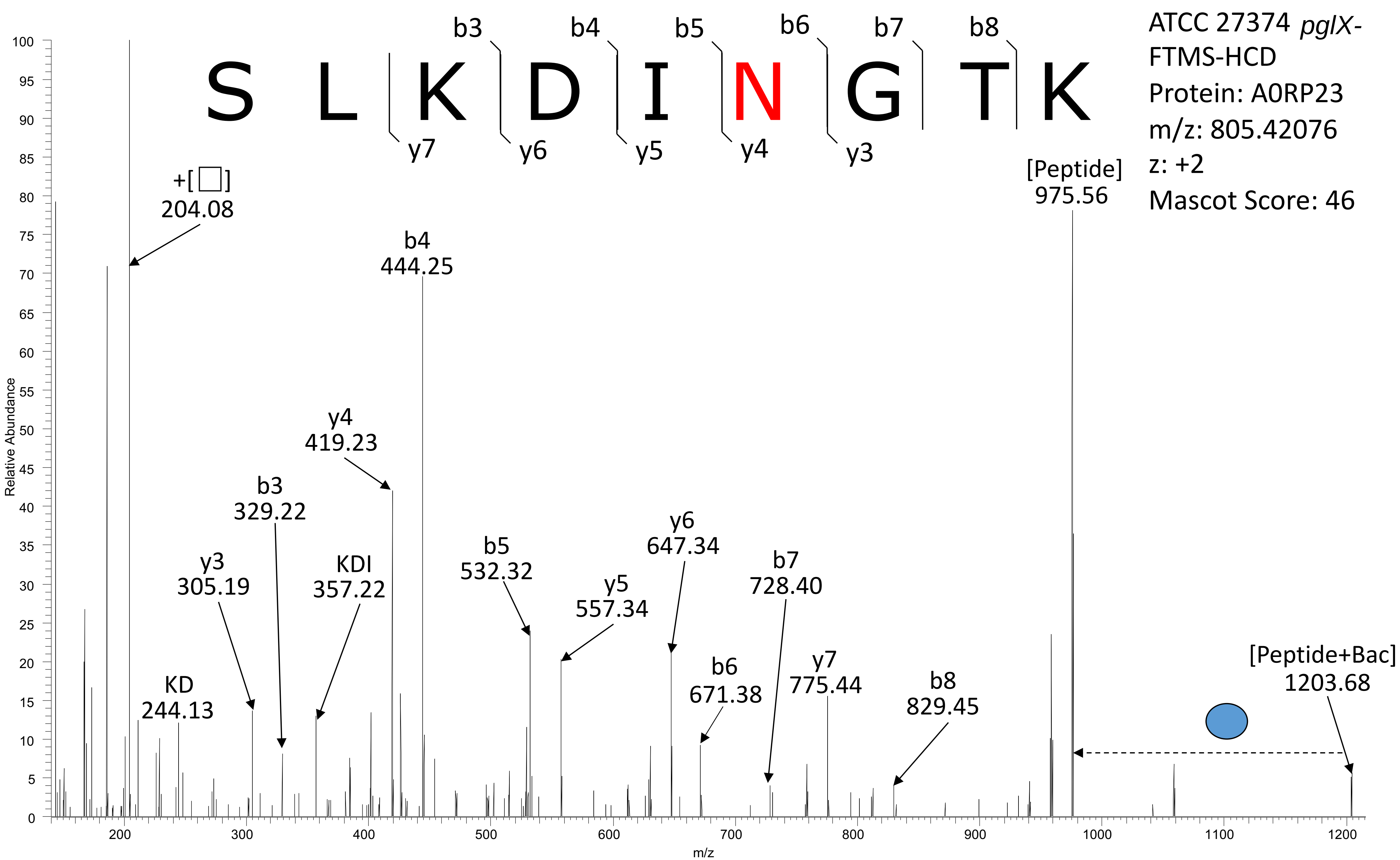

ATCC 27374 *pglX*-
FTMS-HCD
Protein: A0RP23
m/z: 805.42076
z: +2
Mascot Score: 46
S L K D I N G T K
b3 b4 b5 b6 b7 b8
y7 y6 y5 y4 y3
+[□] 204.08
b4 444.25
y4 419.23
b3 329.22
y3 305.19
KDI 357.22
KD 244.13
b5 532.32
y5 557.34
y6 647.34
b6 671.38
b7 728.40
y7 775.44
b8 829.45
[Peptide] 975.56
[Peptide+Bac] 1203.68
Relative Abundance
m/z

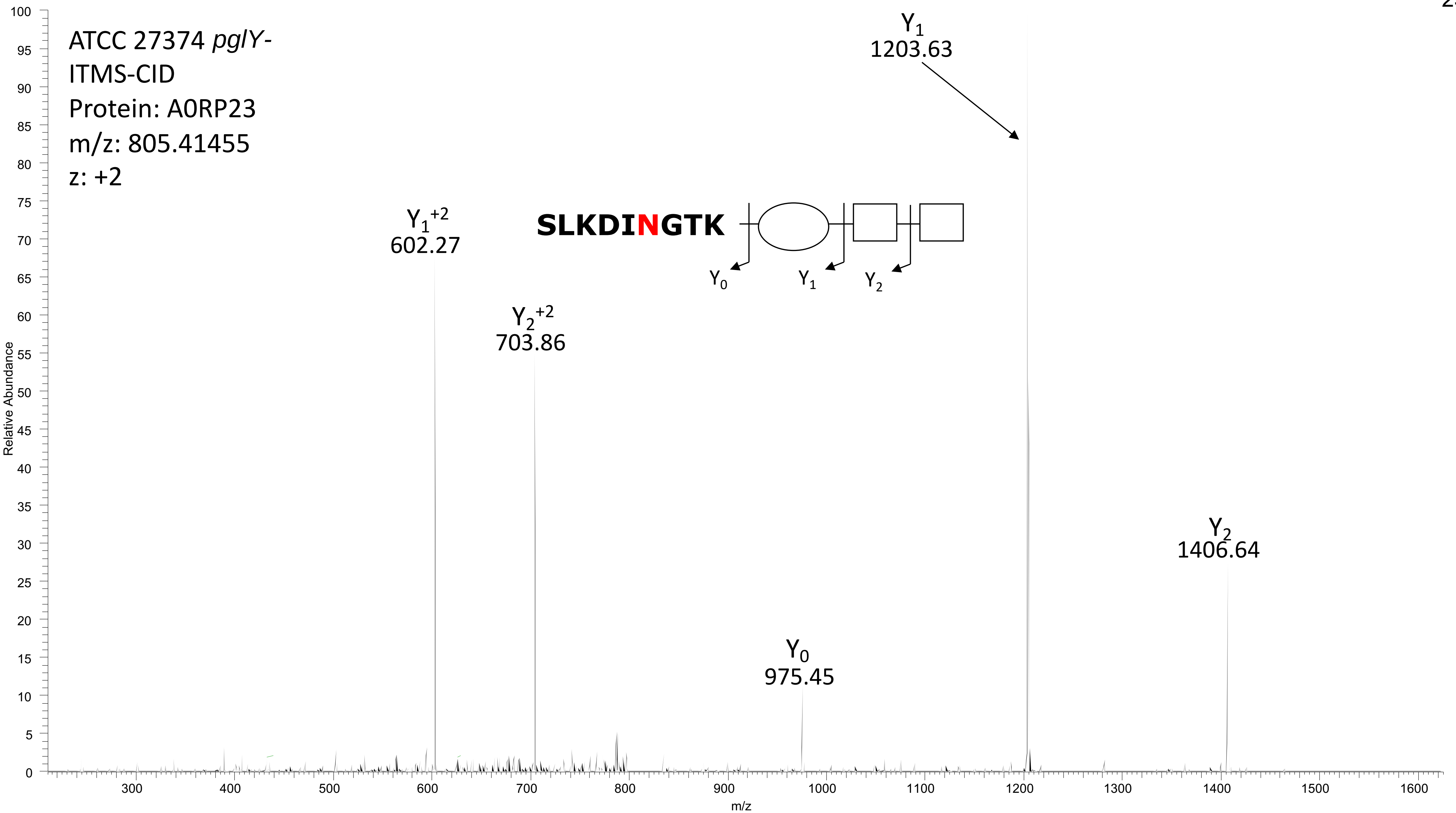

ATCC 27374 *pglY-*
ITMS-CID
Protein: A0RP23
m/z: 805.41455
z: +2
SLKDINGTK
Y_0
Y_1
Y_2
Y_1 1203.63
Y_1^{+2} 602.27
Y_2^{+2} 703.86
Y_2 1406.64
Y_0 975.45
Relative Abundance
m/z

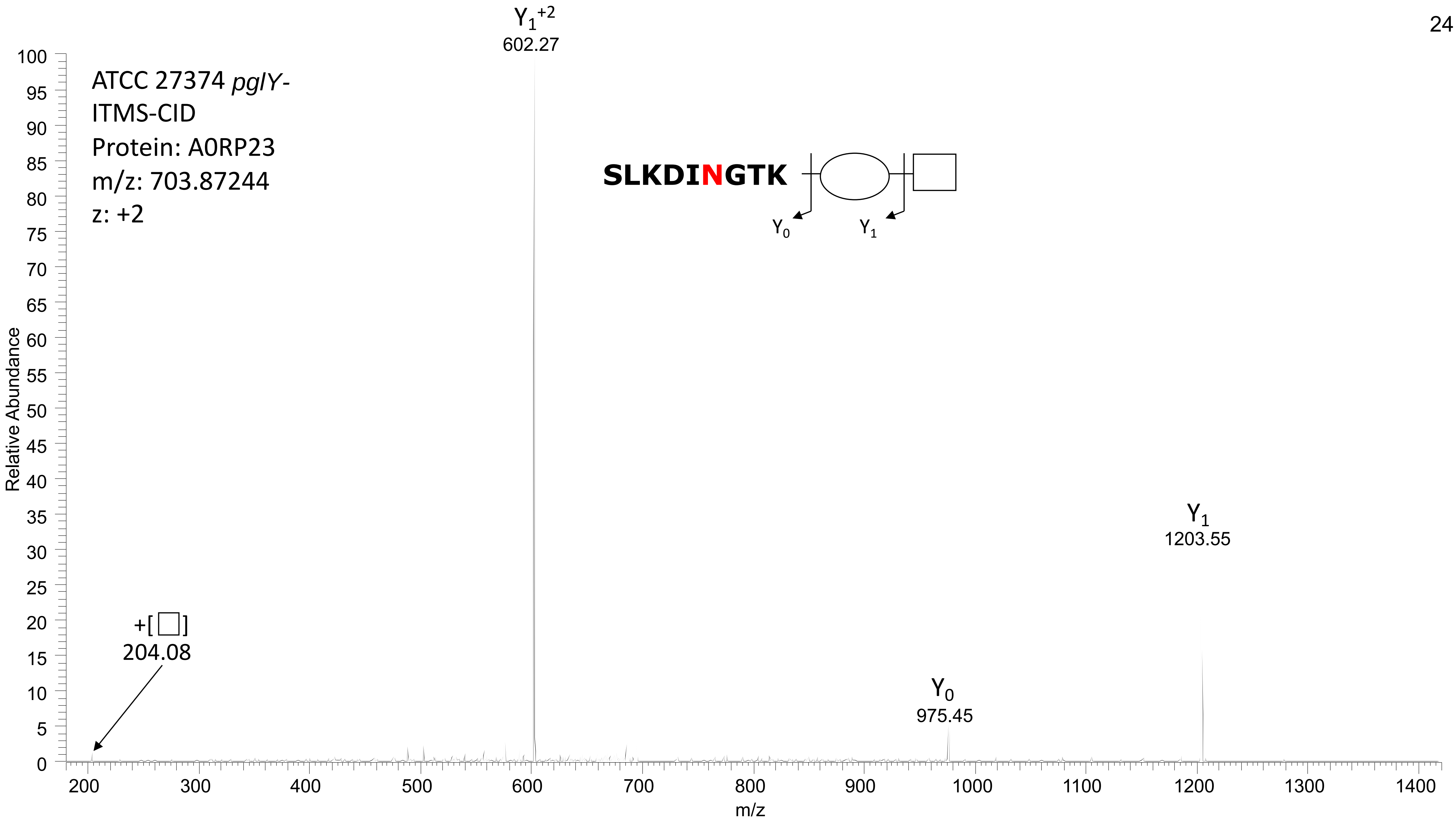

ATCC 27374 *pglY-*
ITMS-CID
Protein: A0RP23
m/z: 703.87244
z: +2
SLKDINGTK
Y0
Y1
Y1+2
602.27
Y1
1203.55
Y0
975.45
+[ ]
204.08
Relative Abundance
m/z

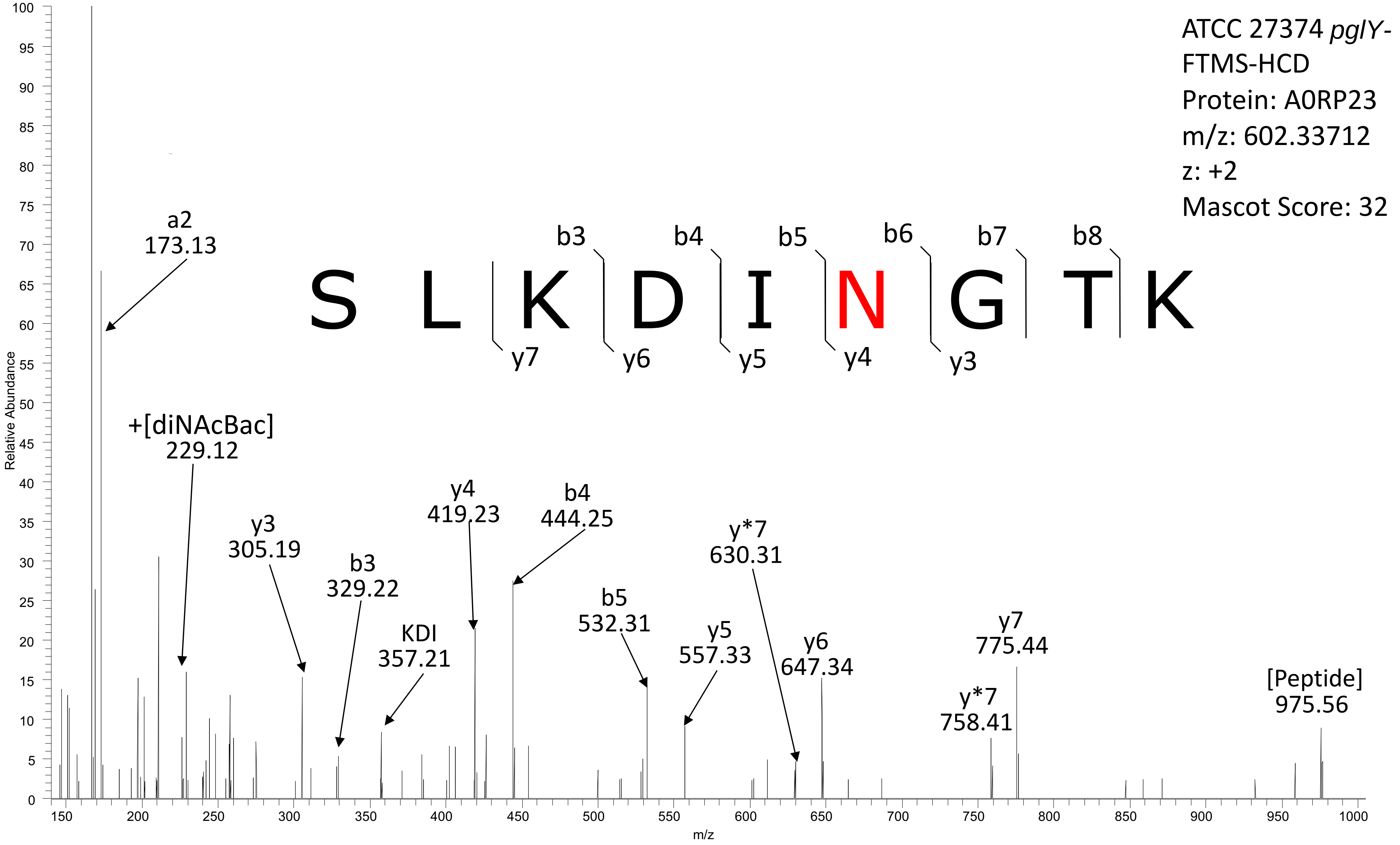
ATCC 27374 pglY-
FTMS-HCD
Protein: A0RP23
m/z: 602.33712
z: +2
Mascot Score: 32
S L K D I N G T K
b3 b4 b5 b6 b7 b8
y7 y6 y5 y4 y3
a2 173.13
+[diNAcBac] 229.12
y3 305.19
b3 329.22
KDI 357.21
y4 419.23
b4 444.25
b5 532.31
y5 557.33
y*7 630.31
y6 647.34
y*7 758.41
y7 775.44
[Peptide] 975.56
Relative Abundance
m/z

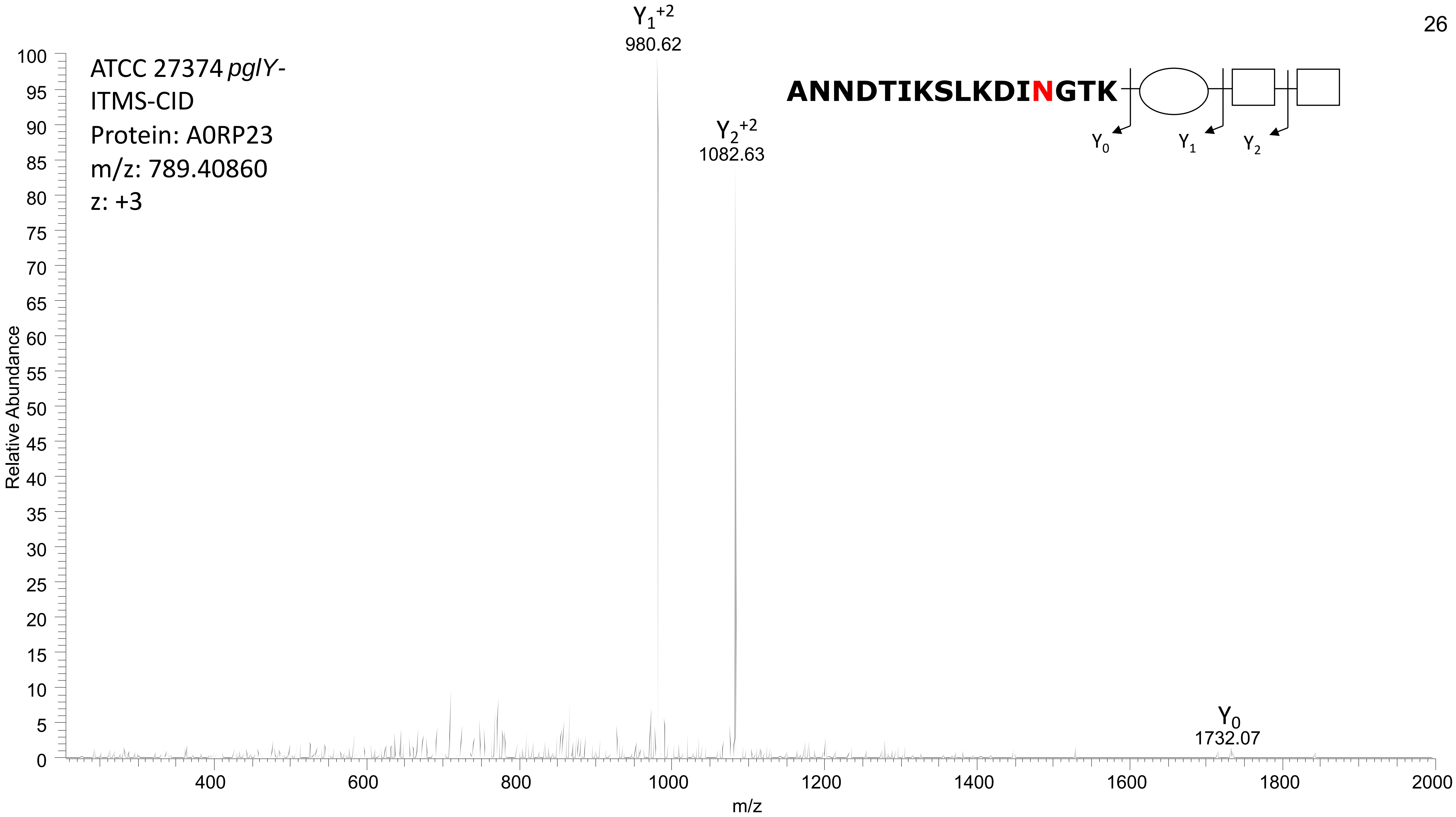
ATCC 27374 *pglY-*
ITMS-CID
Protein: A0RP23
m/z: 789.40860
z: +3
ANNDTIKSLKDINGTK
Y_0
Y_1
Y_2
Y_1^{+2}
980.62
Y_2^{+2}
1082.63
Y_0
1732.07
Relative Abundance
m/z

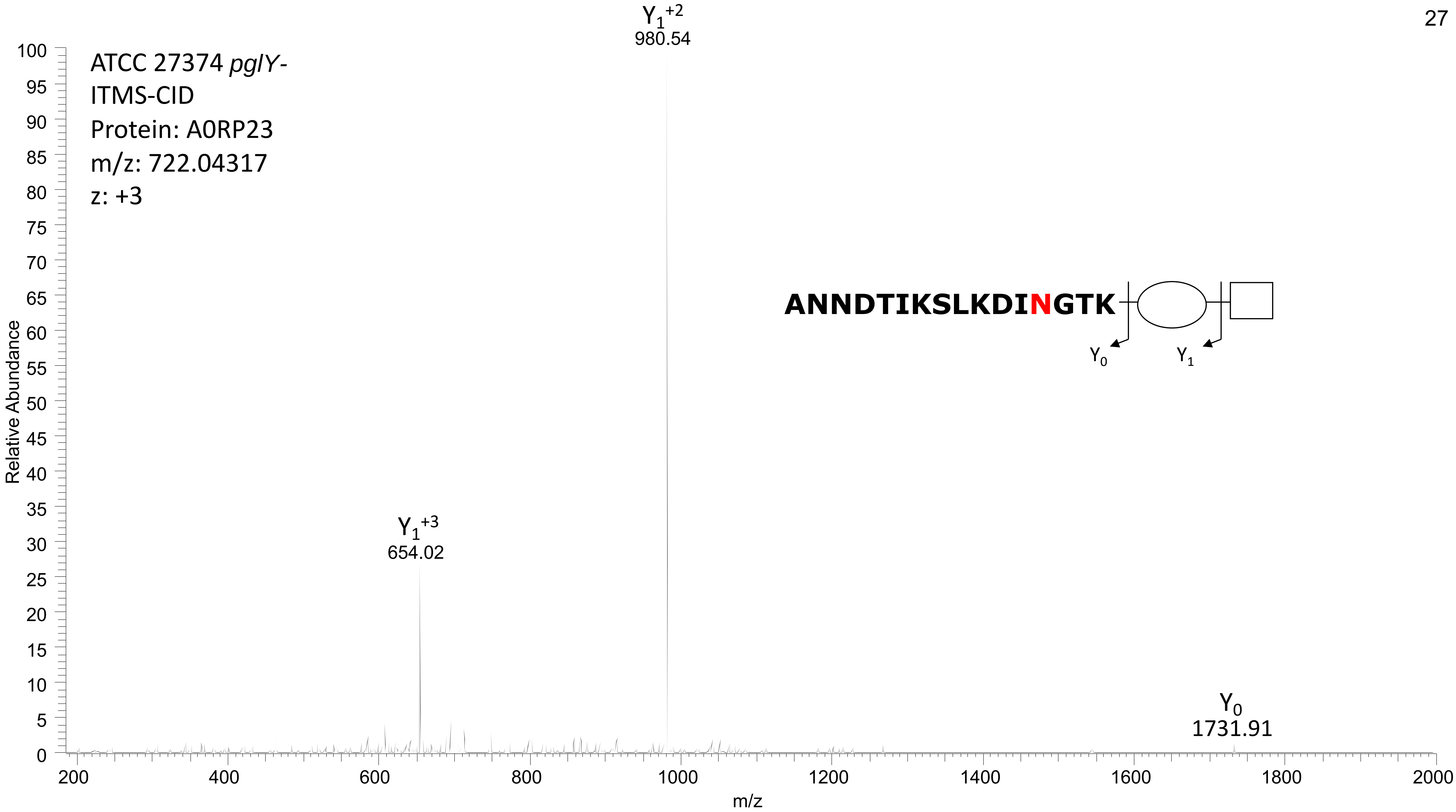
ATCC 27374 pglY-
ITMS-CID
Protein: A0RP23
m/z: 722.04317
z: +3
Y1+2
980.54
Y1+3
654.02
Y0
1731.91
ANNDTIKSLKDINGTK
Y0
Y1
Relative Abundance
m/z
0
5
10
15
20
25
30
35
40
45
50
55
60
65
70
75
80
85
90
95
100
200
400
600
800
1000
1200
1400
1600
1800
2000

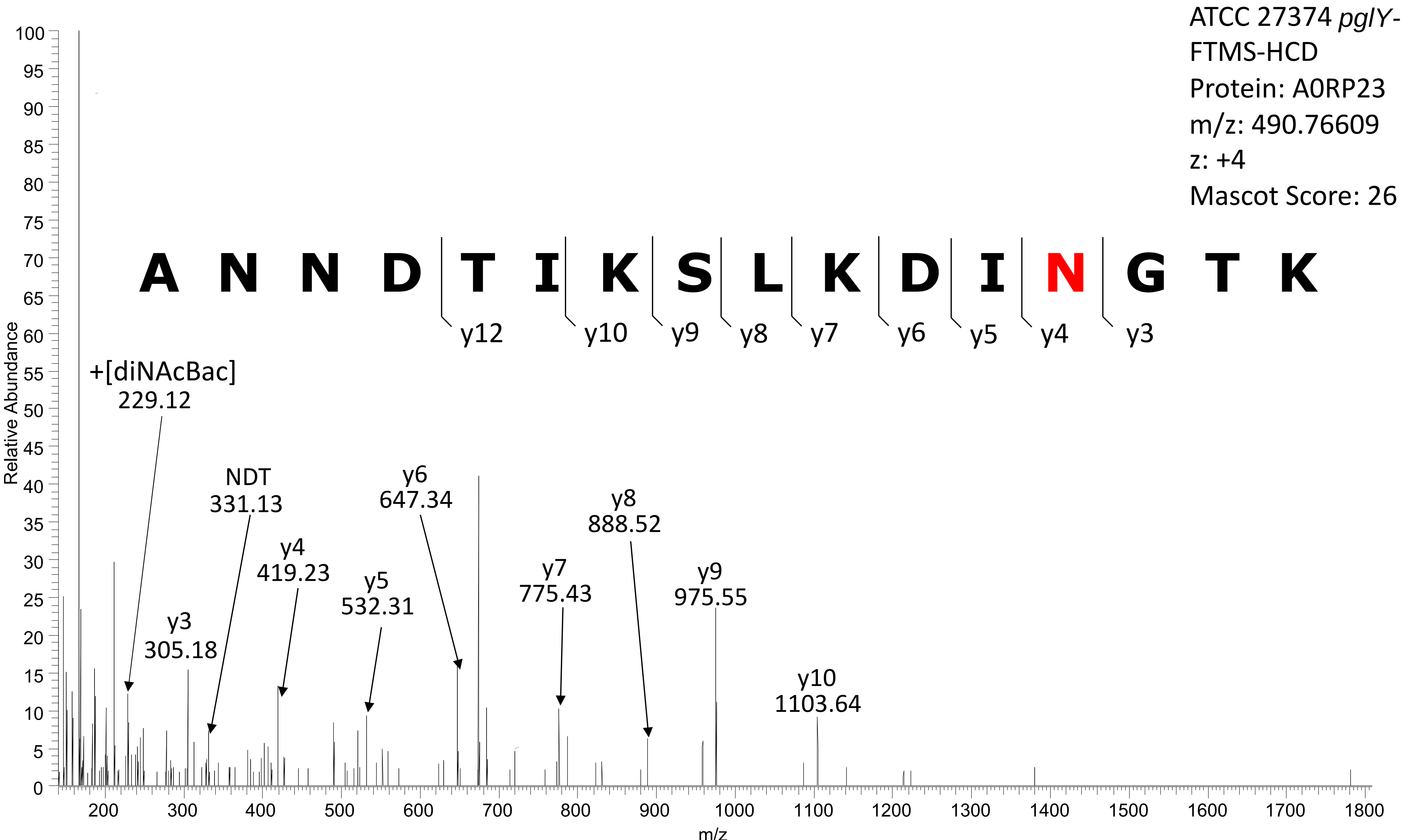
ATCC 27374 *pglY-*
FTMS-HCD
Protein: A0RP23
m/z: 490.76609
z: +4
Mascot Score: 26
A N N D T I K S L K D I N G T K
y12
y10
y9
y8
y7
y6
y5
y4
y3
+[diNAcBac]
229.12
NDT
331.13
y3
305.18
y4
419.23
y5
532.31
y6
647.34
y7
775.43
y8
888.52
y9
975.55
y10
1103.64
Relative Abundance
m/z
