# Supplementary material for: Influence of Protein Glycosylation on Campylobacter fetus Physiology
Source: Front Microbiol. 2020 Jun 17;11:1191. doi: 10.3389/fmicb.2020.01191 (PMC7313396; doi:10.3389/fmicb.2020.01191)
Supplement: Supplementary file 4 [file Data_Sheet_2.PDF]

Supplementary MS data 2

N-glycosylation of CmeA in the heterologous *E. coli* complementation system

Modification (N-glycan mass)	Peptide XIC (m/z)	z	Identified by MS + MS in p*pgl*-derivative + complement, (charge, z)	Page(s)
unmodifed	467.2251<br>700.3337	3<br>2	All samples	-
diNAcBac-HexNAc$_5$-Hex (1405.555)	935.7436<br>1403.1183	3<br>2	*pgl*^{WT}, (3, 2)<br>*pglA*^{mut} + *Cj-pglA*, (3, 2)<br>*pglA*^{mut} + *Cff-pglA*, (2)<br>*pglJ*^{mut} + *Cj-pglJ*, (2)<br>*pglJ*^{mut} + *Cff-pglJ*, (2)<br>*pglH*^{mut} + *Cj-pglH* , (3, 2)	3, 4<br>3, 4<br>4<br>4<br>4<br>3, 4
diNAcBac (228.11)	543.2638<br>814.3918	3<br>2	*pglA*^{mut} , (2)<br>*pglA*^{mut} + *Cj-pglJ* (3, 2)<br>*pglA*^{mut} + *Cff-pglJ* (3, 2)<br>*pglJ*^{mut} + *Cj-pglA* (2)<br>*pglJ*^{mut} + *Cff-pglA* (2)	5<br>5 ,6<br>5 ,6<br>5<br>5
diNAcBac-HexNAc (431.19724)	610.9575<br>915.932	3<br>2	*pglJ*^{mut}, (3, 2)<br>*pglJ*^{mut} + *Cj-pglA*, (3, 2)<br>*pglJ*^{mut} + *Cff-pglA* , (3, 2)<br>*pglH*^{mut} + *Cff-pglY*, (2)<br>*pglHI*^{mut}, (2)<br>*pglHI*^{mut} + *Cj-pglH*, (2)<br>*pglHI*^{mut} + *Cff-pglY*, (2)	7, 8, 9<br>7, 8, 9<br>7, 8, 9<br>8, 9<br>8, 9<br>8, 9<br>8, 9
diNAcBac-HexNAc$_2$ (634.27681)	1017.4721<br>678.6502	2<br>3	*pglH*^{mut}, (2, 3)<br>*pglHI*^{mut}, (2, 3)<br>*pglH*^{mut} + *Cff-pglX*, (2)<br>*pglH*^{mut} + *Cff-pglY*, (2)<br>*pglHI*^{mut} + *Cj-pglH*, (2, 3)<br>*pglHI*^{mut} + *Cff-pglX*, (2)<br>*pglHI*^{mut} + *Cff-pglY*, (2, 3)	10, 11<br>10, 11<br>10<br>10<br>10, 11<br>10<br>10, 11
diNAcBac-HexNAc$_5$ (1243.51657)	881.7306<br>1322.0914	3<br>2	*pglI*^{mut}, (3, 2)<br>*pglI*^{mut} + *Cj-pglH*, (3, 2)<br>*pglI*^{mut} + *Cff-pglY*, (3, 2)<br>*pglI*^{mut} + *Cff-pglX*, (2)<br>*pglI*^{mut} + *Cff-pglXY*, (3, 2)<br>*pglI*^{mut} + *Cff-pglX+Y*, (3, 2)<br>*pglHI*^{mut} + *Cj-pglH* ,(3, 2)	12, 13<br>12, 13<br>12, 13<br>13<br>12, 13<br>12, 13<br>12, 13

diNAcBac-HexNAc$_6$ (1446.59476)	949.4234<br>1423.632	3<br>2	*pglI*^{mut}, (3, 2)<br>*pglI*^{mut} + *Cj-pglH*, (3, 2)<br>*pglI*^{mut} + *Cff-pglX*, (3)<br>*pglI*^{mut} + *Cff-pglY*, (3, 2)<br>*pglI*^{mut} + *Cff-pglXY*, (3)<br>*pglI*^{mut} + *Cff-pglX+Y*, (3)<br>*pglHI*^{mut} + *Cj-pglH* , (3, 2)	14, 15<br>14, 15<br>14<br>14, 15<br>14<br>14<br>14, 15
diNAcBac-HexNAc$_2$-Hex (796.3297)	732.6683<br>1098.4983	3<br>2	*pglHI*^{mut}, (3, 2)<br>*pglH*^{mut} + *Cff-pglX*, (2)<br>*pglH*^{mut} + *Cff-pglY*, (3, 2)<br>*pglH*^{mut} + *Cff-pglXY* (2)<br>*pglHI*^{mut} + *Cj-pglH*, (3, 2)<br>*pglHI*^{mut} + *Cff-pglY*, (3, 2)	16, 17<br>17<br>16, 17<br>17<br>16, 17<br>16, 17
diNAcBac-HexNAc$_3$-Hex$_2$ (1161.46207)	854.3791<br>1281.0647	3<br>2	No MS + MS data (3)<br>*pglH*^{mut} + *Cff-pglX*, (2)<br>*pglH*^{mut} + *Cff-pglXY*, (2)	-<br>18<br>18
diNAcBac-HexNAc$_3$-Hex (999.40891)	800.3614<br>1200.0382	3<br>2	*pglH*^{mut} + *Cff-pglX*, (3, 2)<br>*pglH*^{mut} + *Cff-pglXY*, (3, 2)<br>*pglI*^{mut} + *Cff-pglX*, (2)<br>*pglHI*^{mut} + *Cff-pglX*, (2)	19, 20<br>19, 20<br>20<br>20
diNAcBac-HexNAc$_2$-Hex$_2$ (958.38115)	1179.5243	2	*pglH*^{mut} + *Cff-pglY*, (2)	21
diNAcBac-HexNAc$_4$-Hex (1202.48905)	868.0548<br>1301.5782	2<br>3	No MS + MS data, (2)<br>*pgl*^{WT}, (3)<br>*pglA*^{mut} + *Cff-pglA*, (3)<br>*pglH*^{mut} + *Cff-pglXY*, (3)	-<br>22<br>22<br>22
diNAcBac-HexNAc$_4$ (1040.43431)	814.0366<br>1220.5509	2<br>3	*pglH*^{mut} + *Cff-pglXY*, (3)<br>*pglH*^{mut} + *Cff-pglX+Y*, (2, 3)<br>*pglI*^{mut} + *Cff-pglY*, (3)<br>*pglI*^{mut} + *Cff-pglXY*, (2, 3)<br>*pglI*^{mut} + *Cff-pglX+Y*, (2, 3)<br>*pglHI*^{mut} + *Cff-pglXY*, (3)<br>*pglHI*^{mut} + *Cff-pglX+Y*, (2, 3)	24<br>23, 24<br>24<br>23, 24<br>23, 24<br>24<br>23, 24
diNAcBac-HexNAc$_3$ (837.35793)	746.3444<br>1119.0127	3<br>2	*pglH*^{mut} + *Cff-pglX+Y*, (2)<br>*pglI*^{mut} + *Cff-pglX*, (2)<br>*pglI*^{mut} + *Cff-pglXY*, (2)<br>*pglI*^{mut} + *Cff-pglX+Y*, (2)<br>*pglHI*^{mut} + *Cff-pglX*, (3, 2)<br>*pglHI*^{mut} + *Cff-pglXY*, (2)<br>*pglHI*^{mut} + *Cff-pglX+Y*, (2)	26<br>26<br>26<br>26<br>25, 26<br>26<br>26

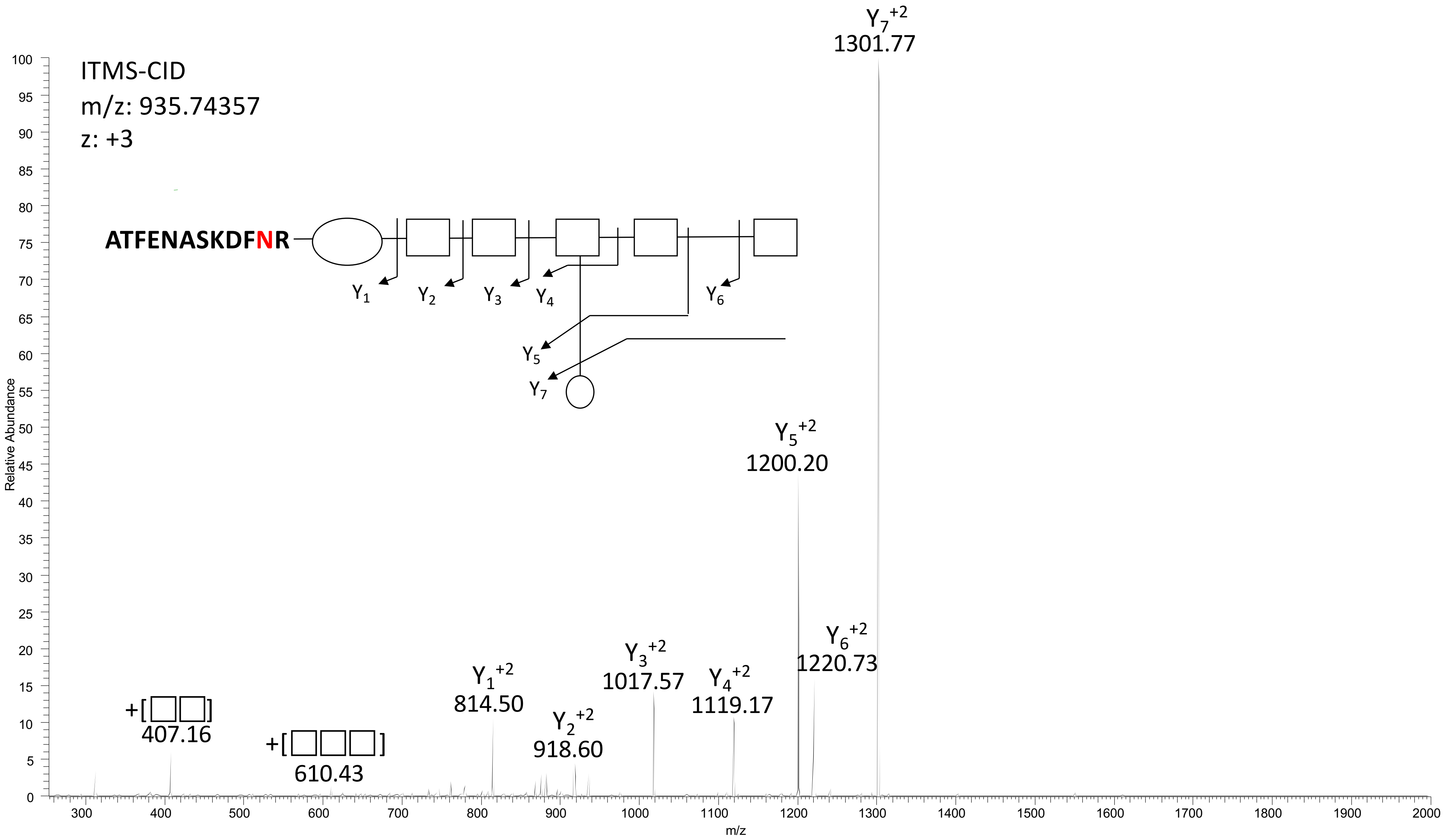
ITMS-CID
m/z: 935.74357
z: +3
ATFENASKDFNR
Y1
Y2
Y3
Y4
Y6
Y5
Y7
Y7+2
1301.77
Y5+2
1200.20
Y6+2
1220.73
Y3+2
1017.57
Y4+2
1119.17
Y1+2
814.50
Y2+2
918.60
407.16
610.43
Relative Abundance
m/z

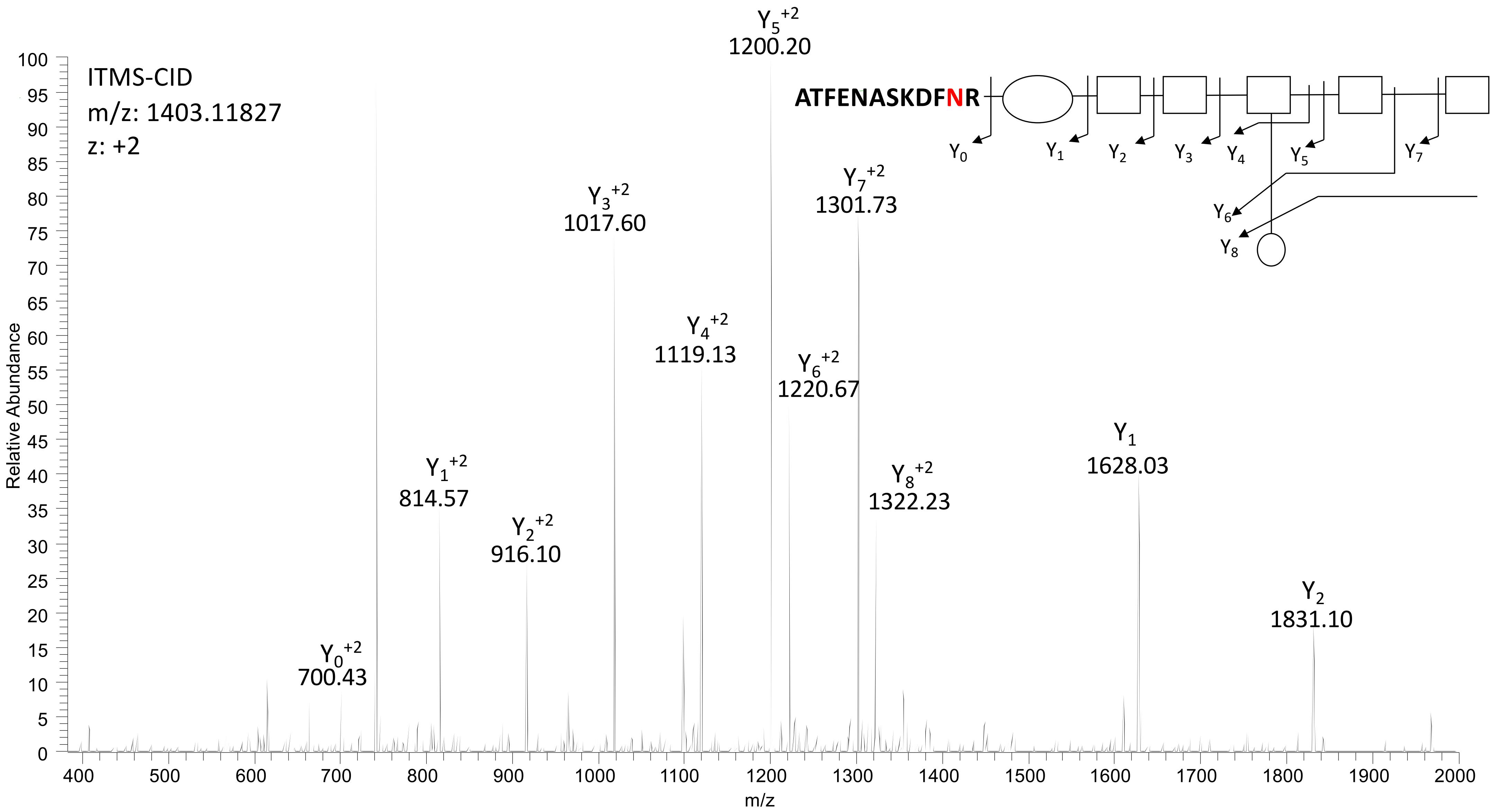

ITMS-CID
m/z: 1403.11827
z: +2
ATFENASKDFNR
Y0
Y1
Y2
Y3
Y4
Y5
Y6
Y7
Y8
Y5+2
1200.20
Y3+2
1017.60
Y7+2
1301.73
Y4+2
1119.13
Y6+2
1220.67
Y1
1628.03
Y1+2
814.57
Y8+2
1322.23
Y2+2
916.10
Y2
1831.10
Y0+2
700.43
Relative Abundance
m/z

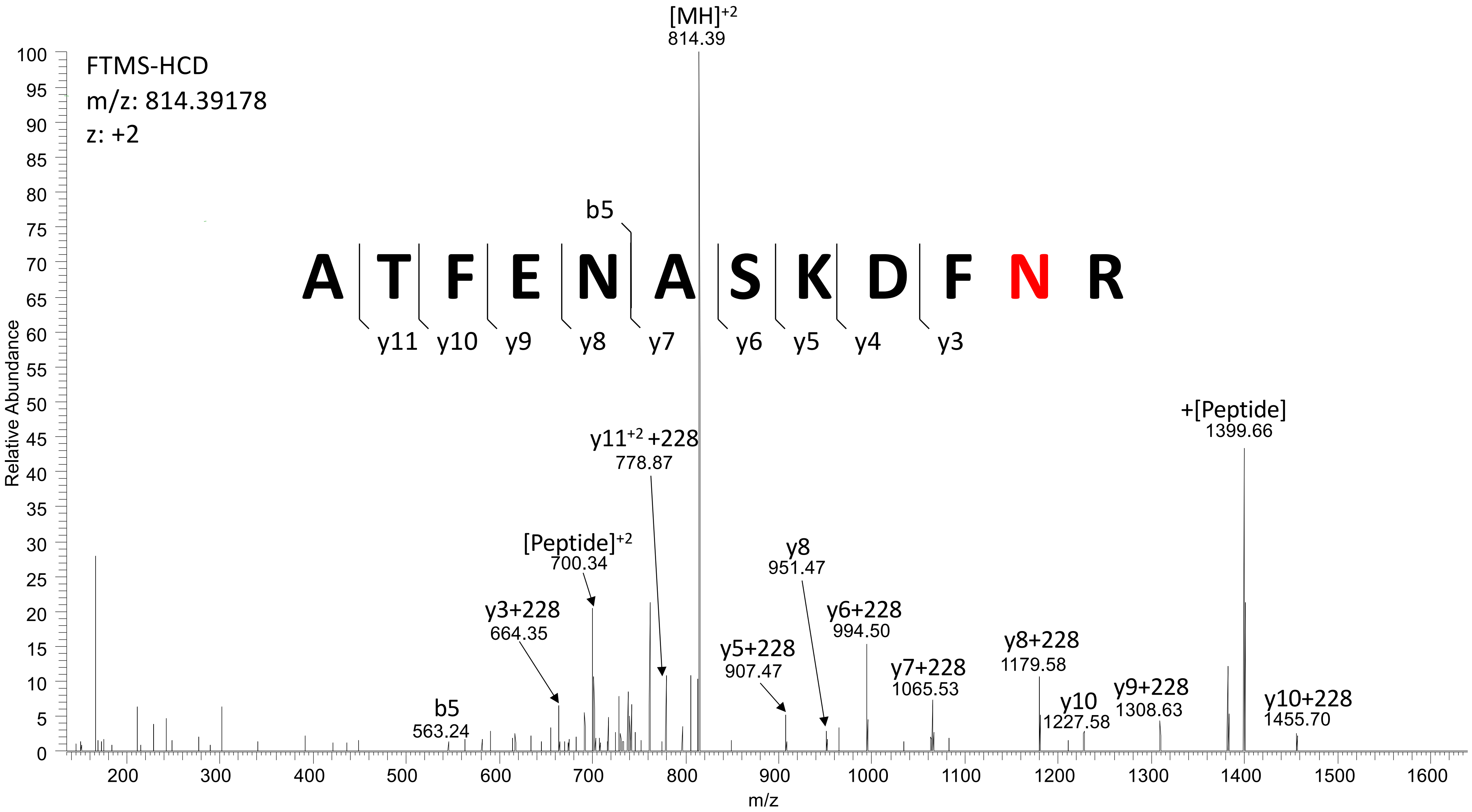
FTMS-HCD
m/z: 814.39178
z: +2
[MH]+2
814.39
b5
A T F E N A S K D F N R
y11 y10 y9 y8 y7 y6 y5 y4 y3
+[Peptide]
1399.66
y11+2 +228
778.87
[Peptide]+2
700.34
y8
951.47
y3+228
664.35
y6+228
994.50
y5+228
907.47
y8+228
1179.58
y7+228
1065.53
b5
563.24
y10
1227.58
y9+228
1308.63
y10+228
1455.70
Relative Abundance
m/z

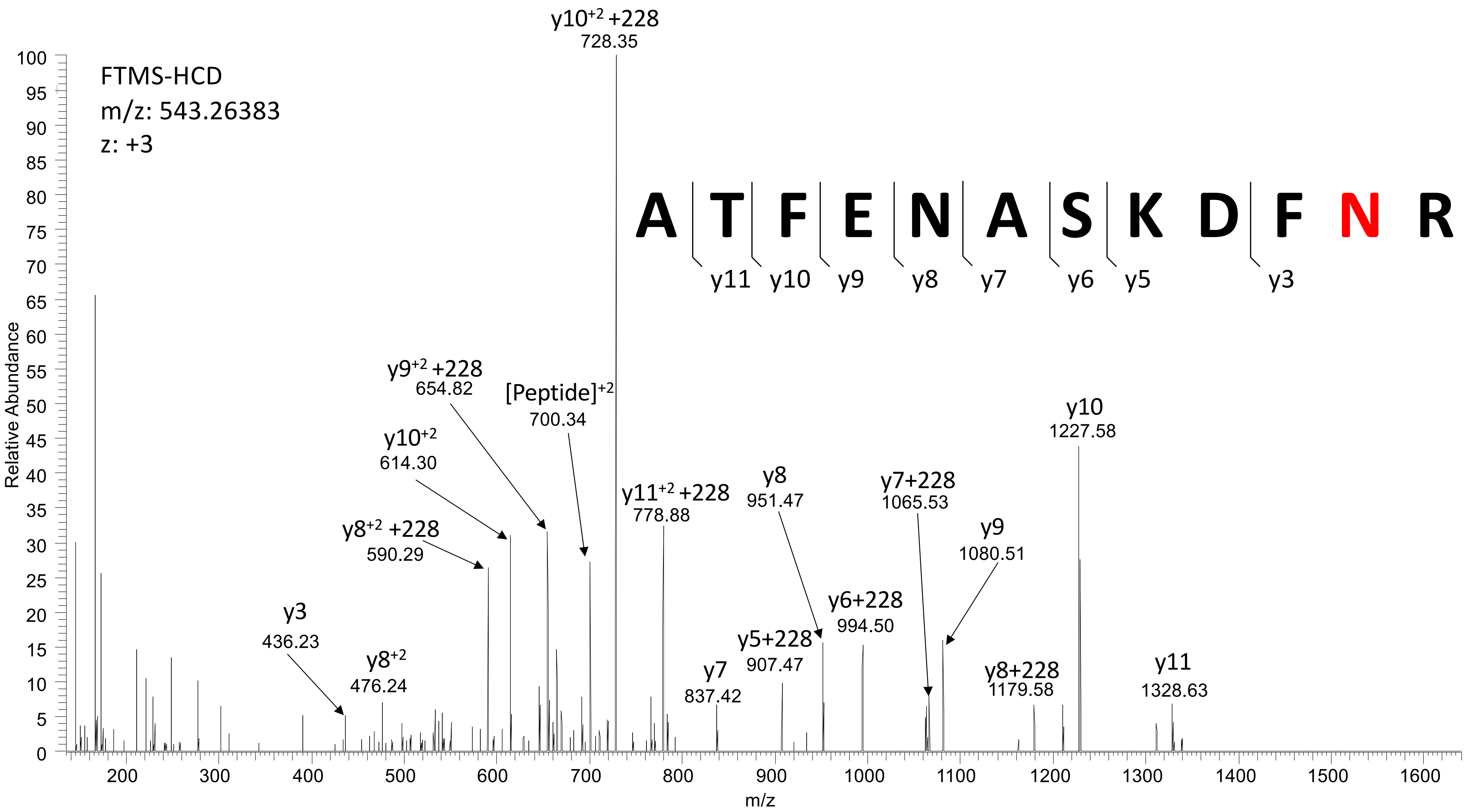
FTMS-HCD
m/z: 543.26383
z: +3
A T F E N A S K D F N R
y11 y10 y9 y8 y7 y6 y5 y3
y10+2 +228 728.35
y9+2 +228 654.82
[Peptide]+2 700.34
y10+2 614.30
y8+2 +228 590.29
y11+2 +228 778.88
y8 951.47
y7+228 1065.53
y10 1227.58
y9 1080.51
y6+228 994.50
y5+228 907.47
y3 436.23
y8+2 476.24
y7 837.42
y8+228 1179.58
y11 1328.63
Relative Abundance
m/z

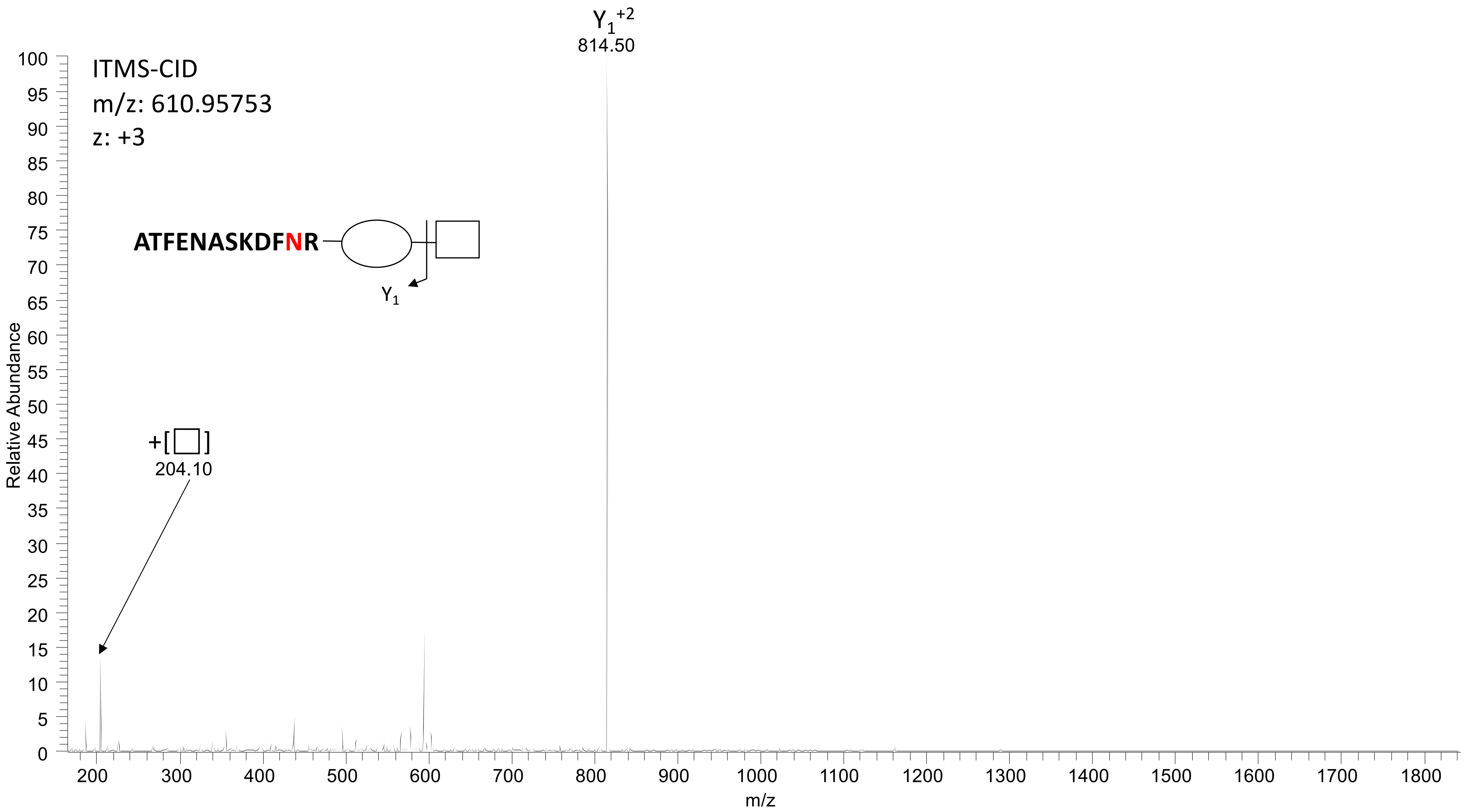

ITMS-CID
m/z: 610.95753
z: +3
ATFENASKDFNR
Y1
Y1+2
814.50
+[]
204.10
Relative Abundance
m/z

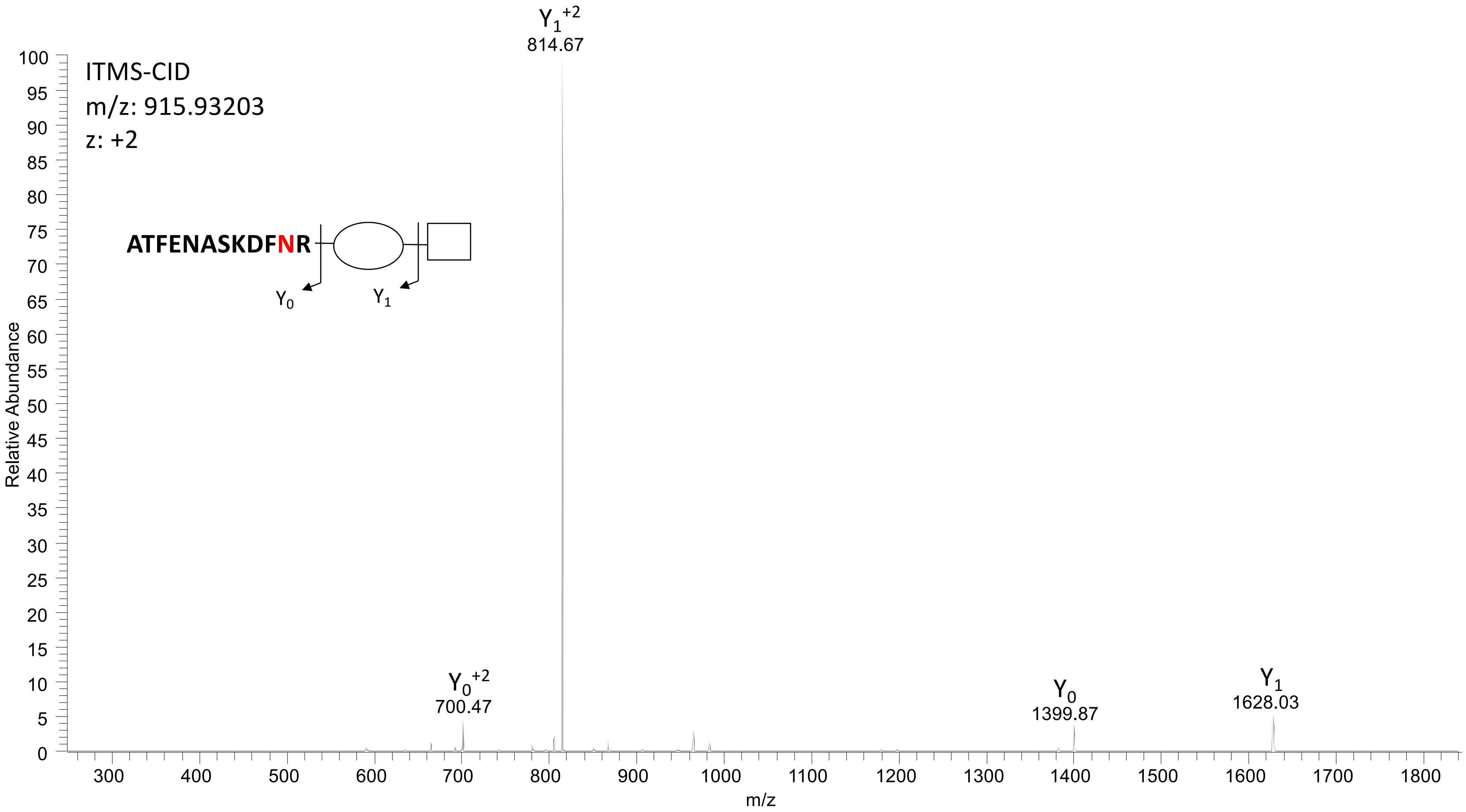
ITMS-CID
m/z: 915.93203
z: +2
ATFENASKDFNR
Y0
Y1
Y1+2
814.67
Y0+2
700.47
Y0
1399.87
Y1
1628.03
Relative Abundance
m/z

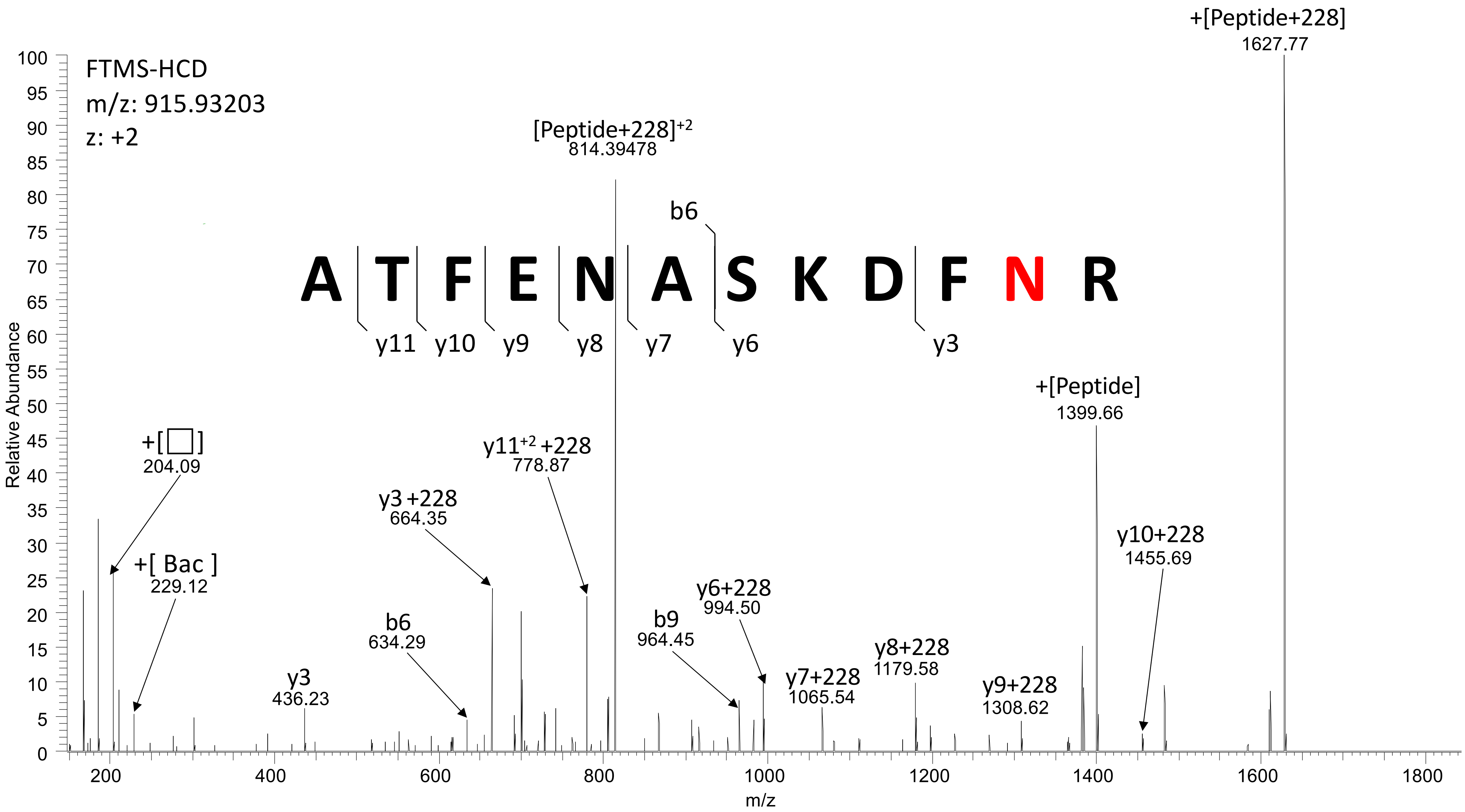
FTMS-HCD
m/z: 915.93203
z: +2
A T F E N A S K D F N R
b6
y11 y10 y9 y8 y7 y6 y3
+[Peptide+228]
1627.77
[Peptide+228]+2
814.39478
+[Peptide]
1399.66
+[ ]
204.09
+[Bac]
229.12
y11+2 +228
778.87
y3 +228
664.35
y10+228
1455.69
y6+228
994.50
b9
964.45
b6
634.29
y7+228
1065.54
y8+228
1179.58
y9+228
1308.62
y3
436.23
Relative Abundance
m/z

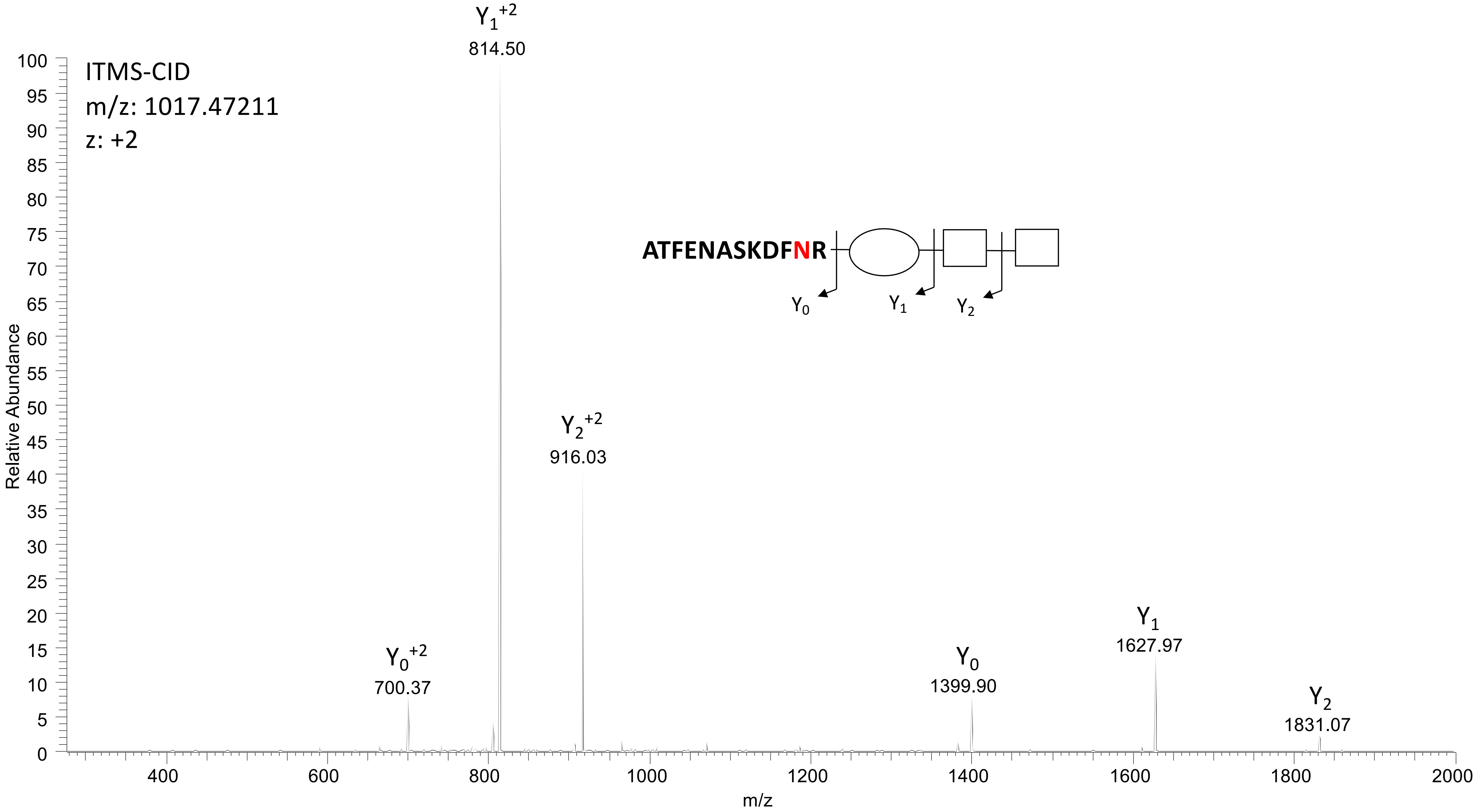

ITMS-CID
m/z: 1017.47211
z: +2
ATFENASKDFNR
Y_0
Y_1
Y_2
Y_1^{+2}
814.50
Y_2^{+2}
916.03
Y_0^{+2}
700.37
Y_0
1399.90
Y_1
1627.97
Y_2
1831.07
Relative Abundance
m/z

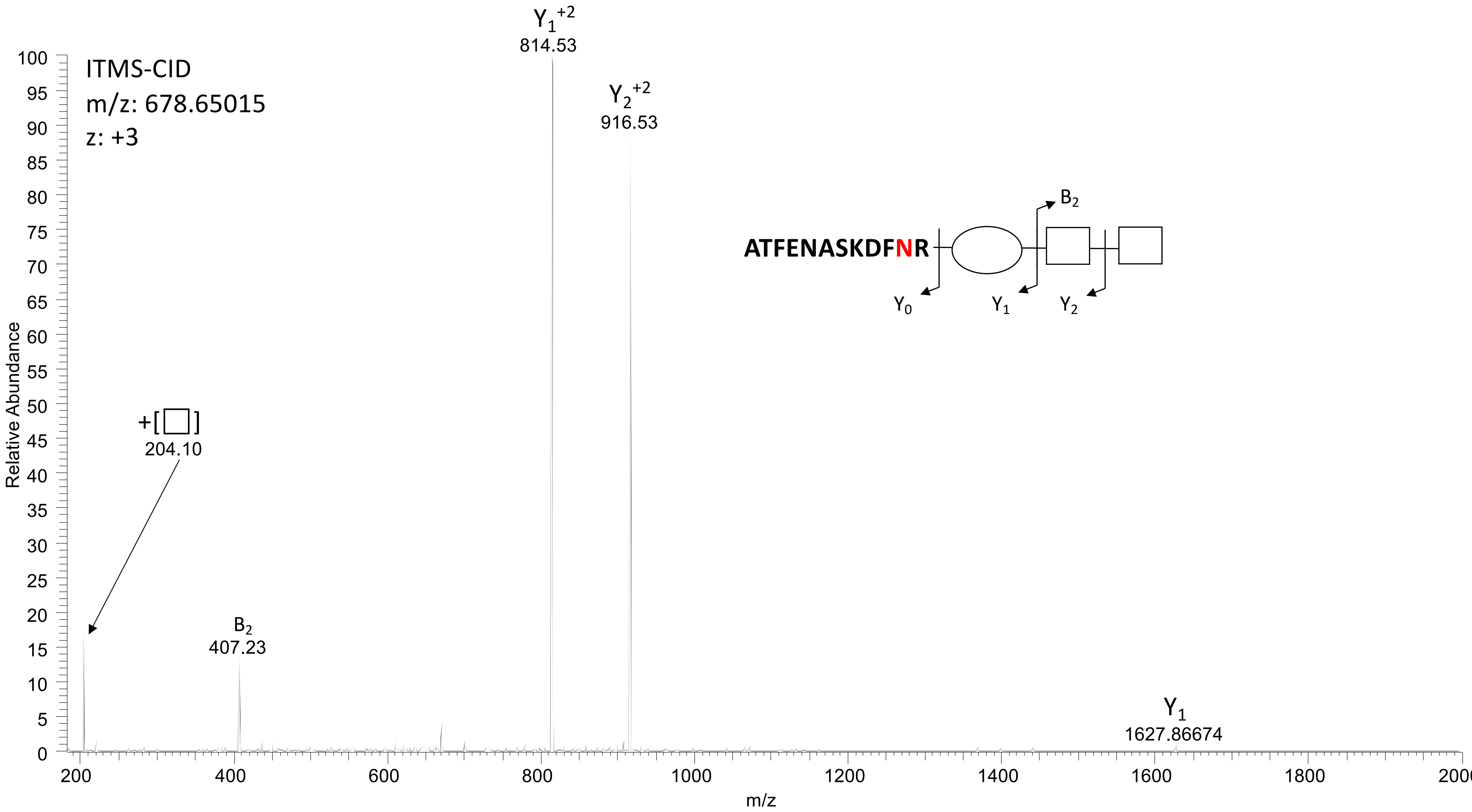

ITMS-CID
m/z: 678.65015
z: +3
Y1+2
814.53
Y2+2
916.53
+[□]
204.10
B2
407.23
Y1
1627.86674
ATFENASKDFNR
B2
Y0
Y1
Y2
Relative Abundance
m/z

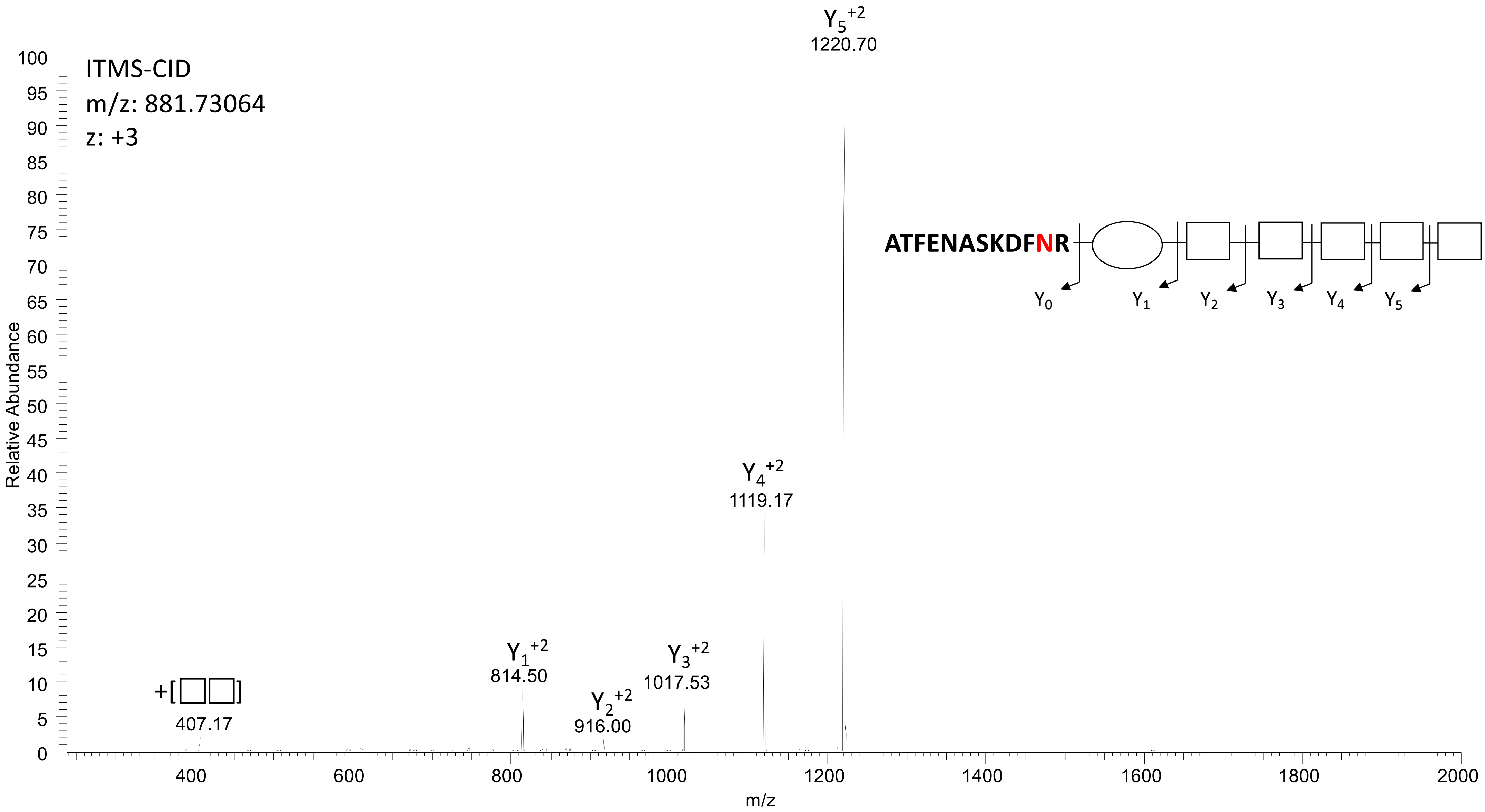
ITMS-CID
m/z: 881.73064
z: +3
Relative Abundance
m/z
ATFENASKDFNR
Y0 Y1 Y2 Y3 Y4 Y5
Y5 +2 1220.70
Y4 +2 1119.17
Y3 +2 1017.53
Y2 +2 916.00
Y1 +2 814.50
+[ ] 407.17

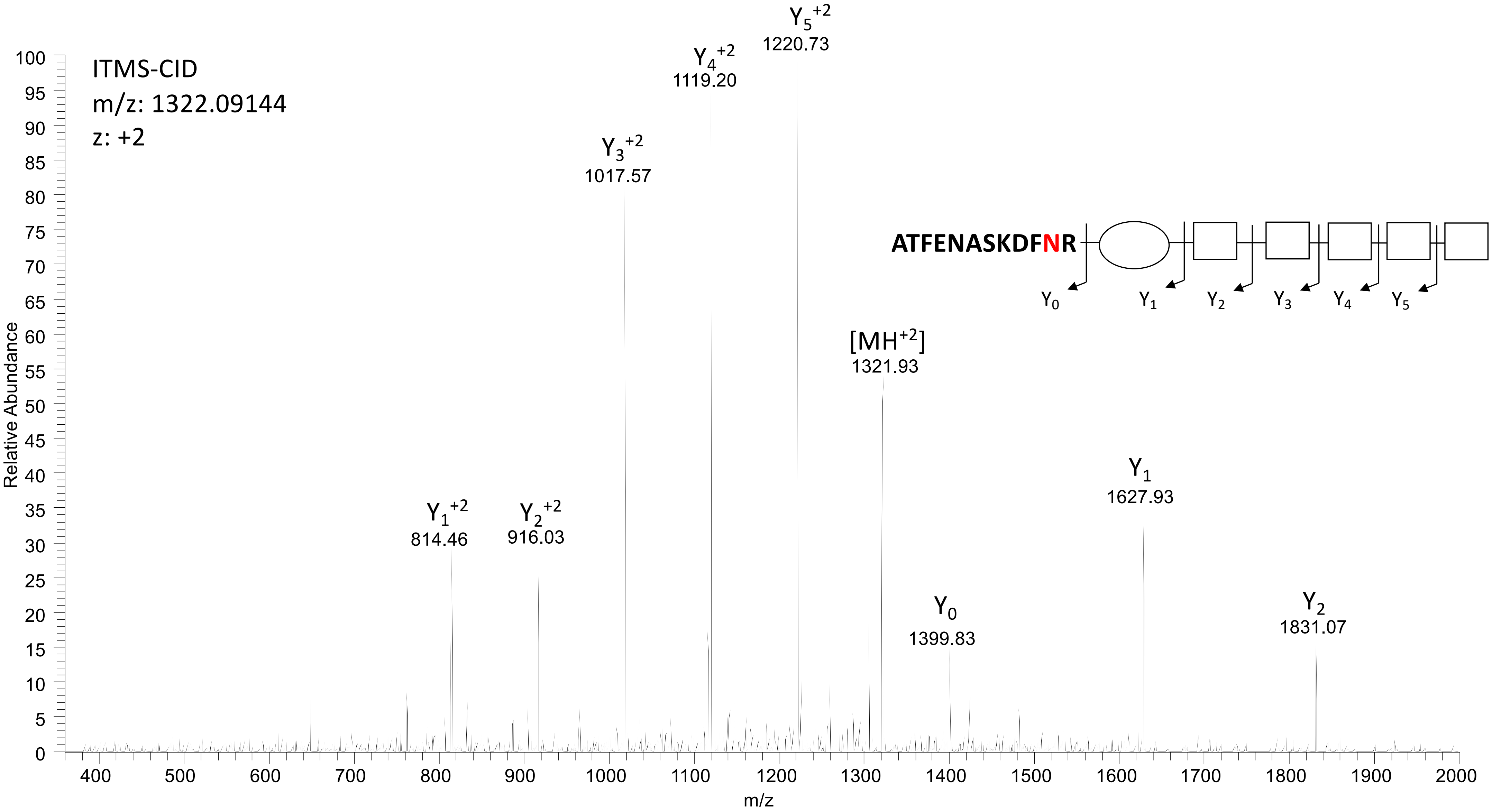
ITMS-CID
m/z: 1322.09144
z: +2
Relative Abundance
m/z
Y1+2
814.46
Y2+2
916.03
Y3+2
1017.57
Y4+2
1119.20
Y5+2
1220.73
[MH+2]
1321.93
Y0
1399.83
Y1
1627.93
Y2
1831.07
ATFENASKDFNR
Y0
Y1
Y2
Y3
Y4
Y5

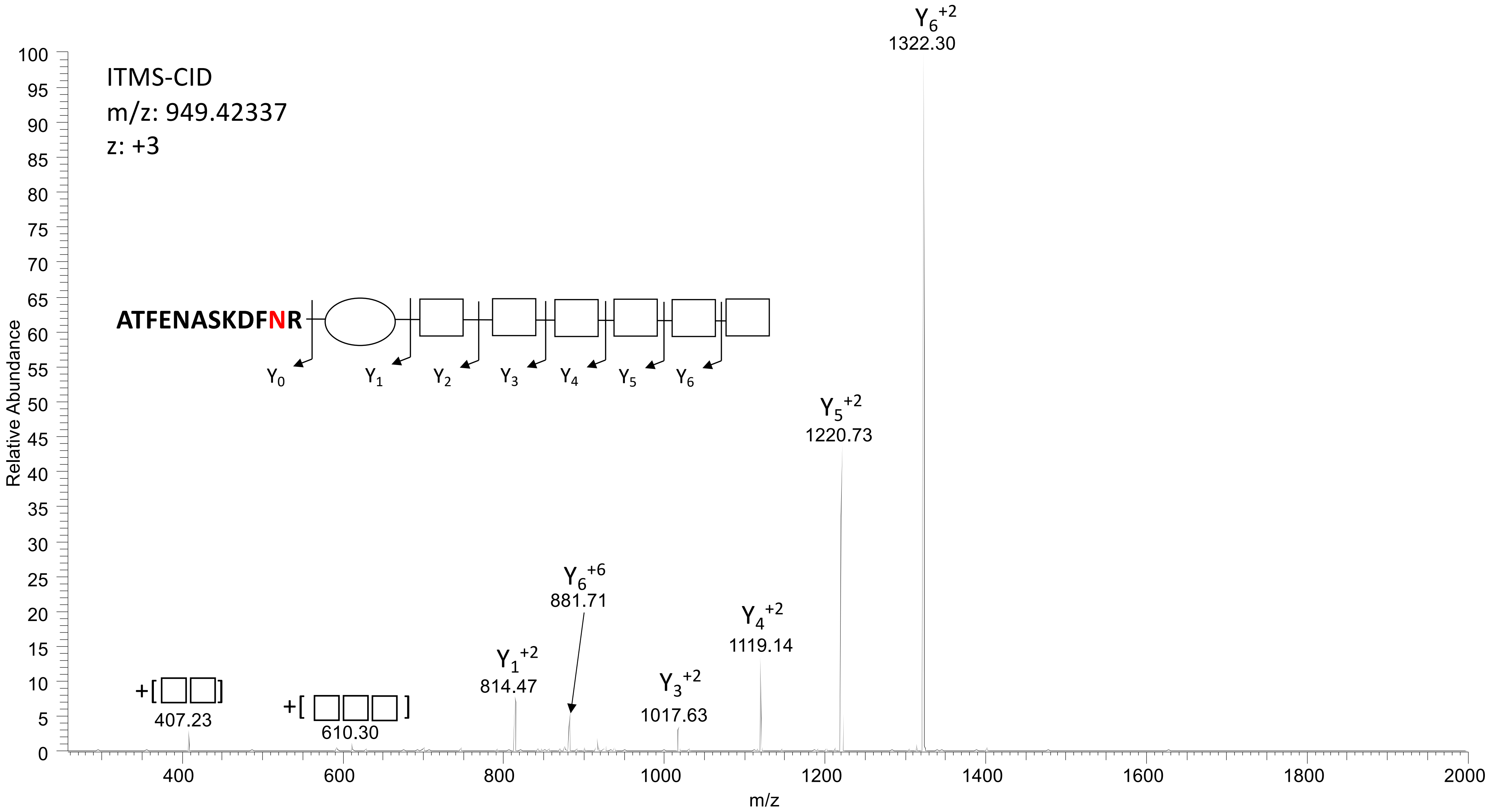
ITMS-CID
m/z: 949.42337
z: +3
ATFENASKDFNR
Y0
Y1
Y2
Y3
Y4
Y5
Y6
Y6+2
1322.30
Y5+2
1220.73
Y6+6
881.71
Y4+2
1119.14
Y1+2
814.47
Y3+2
1017.63
+[ ]
407.23
+[ ]
610.30
Relative Abundance
m/z

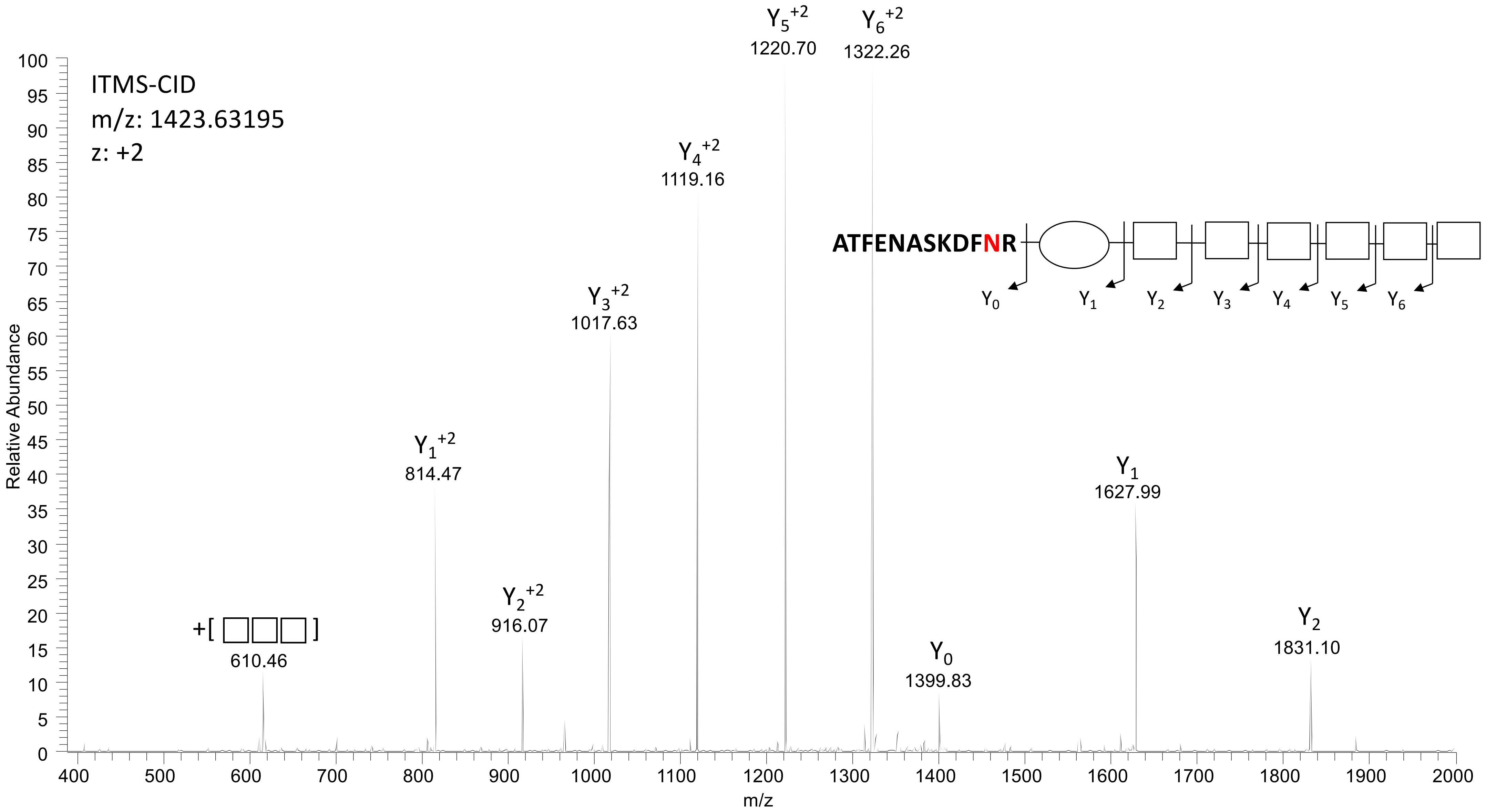
ITMS-CID
m/z: 1423.63195
z: +2
Relative Abundance
m/z
ATFENASKDFNR
Y0
Y1
Y2
Y3
Y4
Y5
Y6
+[ ]
610.46
Y1+2
814.47
Y2+2
916.07
Y3+2
1017.63
Y4+2
1119.16
Y5+2
1220.70
Y6+2
1322.26
Y0
1399.83
Y1
1627.99
Y2
1831.10

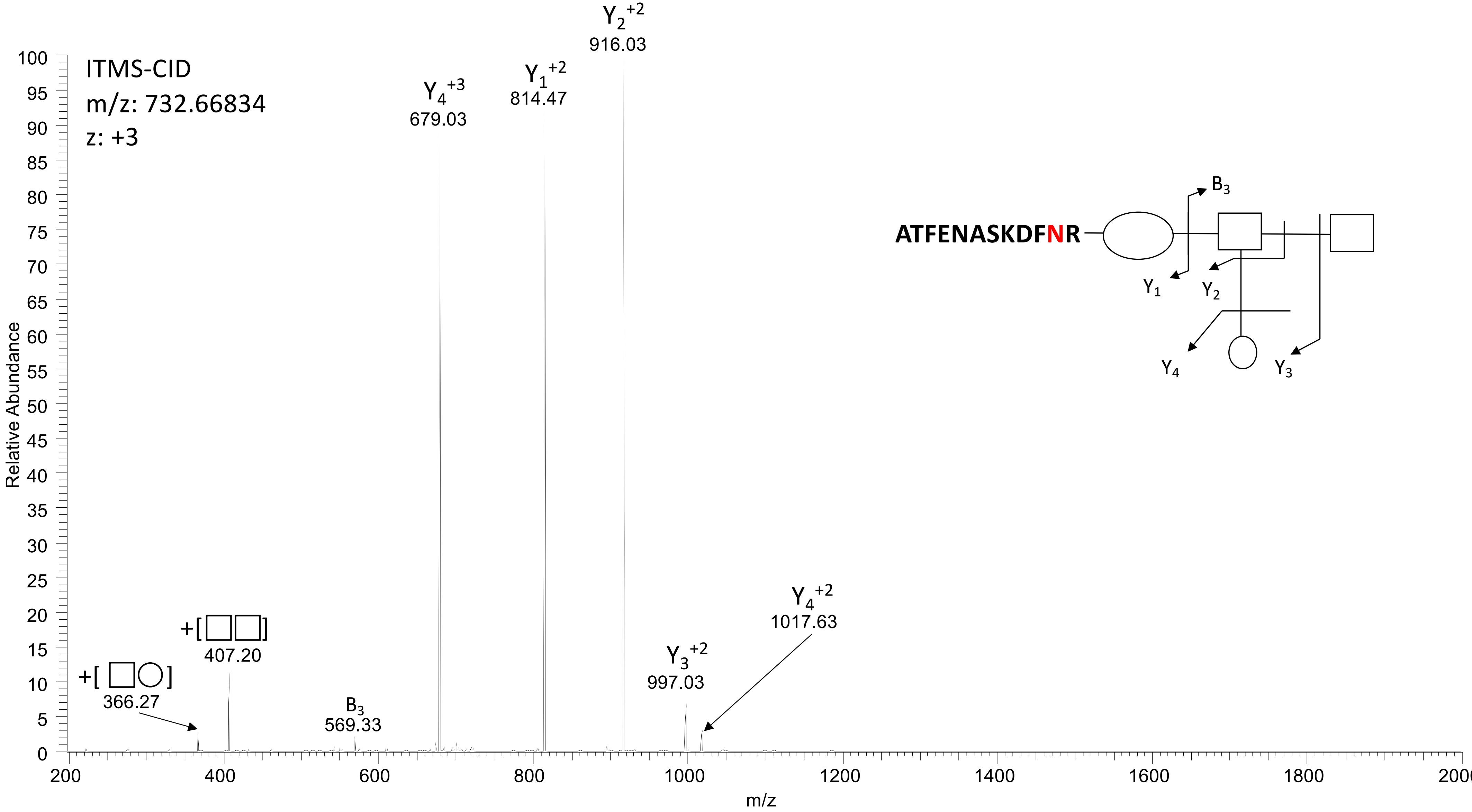

ITMS-CID
m/z: 732.66834
z: +3
Relative Abundance
m/z
Y_2^{+2} 916.03
Y_1^{+2} 814.47
Y_4^{+3} 679.03
Y_4^{+2} 1017.63
Y_3^{+2} 997.03
+[□□] 407.20
+[□○] 366.27
B_3 569.33
ATFENASKDFNR
B_3
Y_1
Y_2
Y_4
Y_3

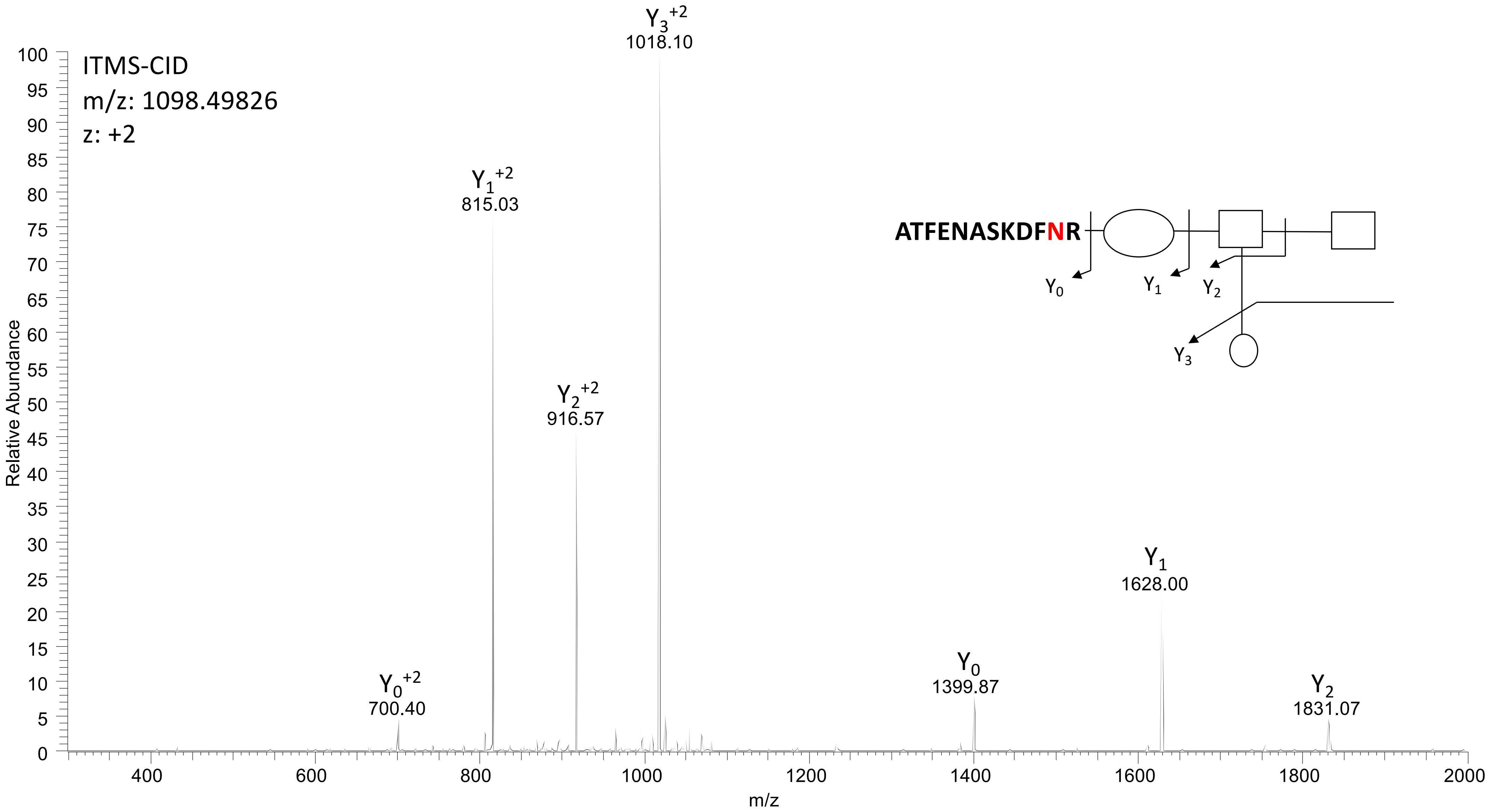

ITMS-CID
m/z: 1098.49826
z: +2
ATFENASKDFNR
Y0
Y1
Y2
Y3
Y3+2
1018.10
Y1+2
815.03
Y2+2
916.57
Y0+2
700.40
Y0
1399.87
Y1
1628.00
Y2
1831.07
Relative Abundance
m/z

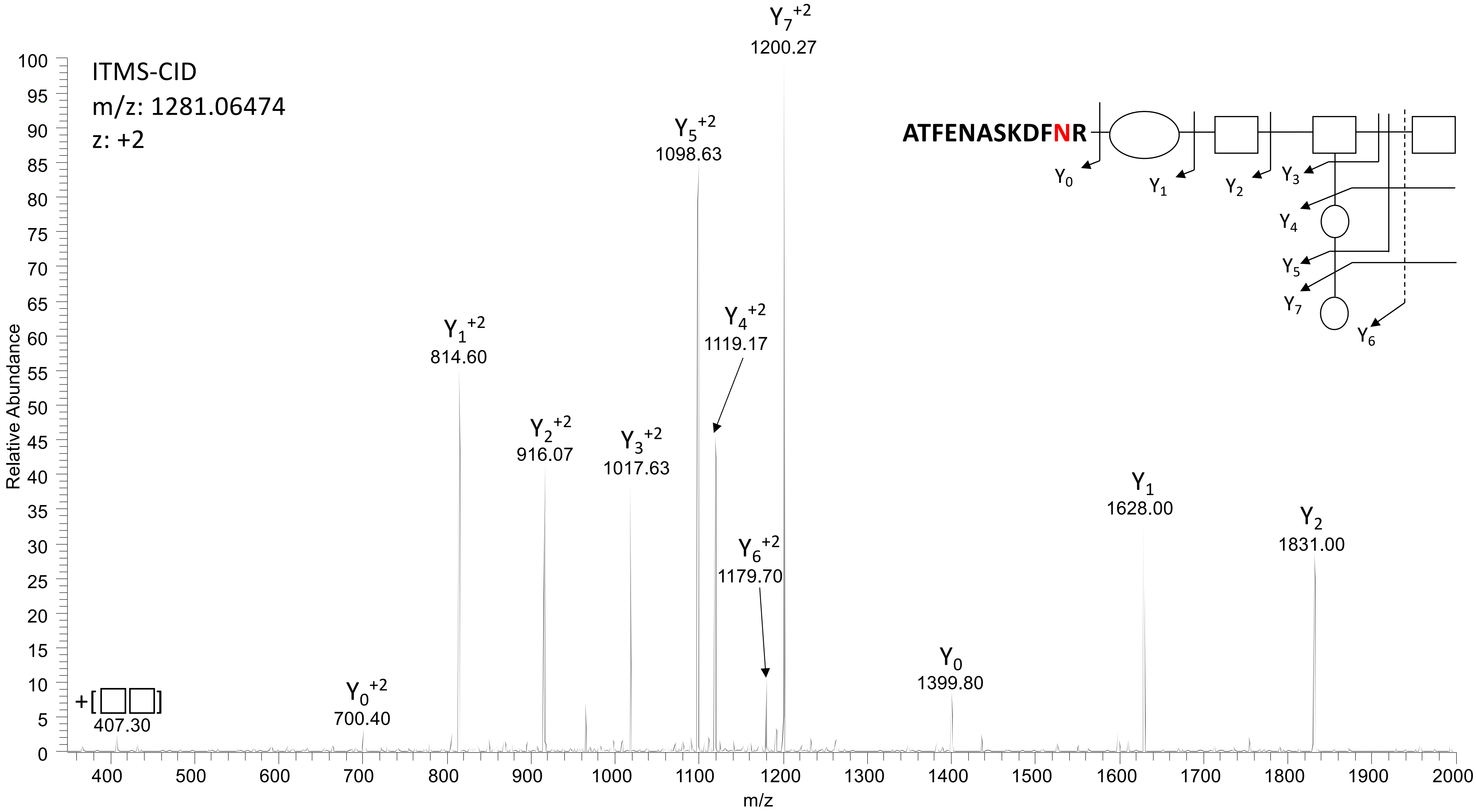
ITMS-CID
m/z: 1281.06474
z: +2
ATFENASKDFNR
Relative Abundance
m/z
Y7+2 1200.27
Y5+2 1098.63
Y4+2 1119.17
Y1+2 814.60
Y2+2 916.07
Y3+2 1017.63
Y6+2 1179.70
Y1 1628.00
Y2 1831.00
Y0 1399.80
Y0+2 700.40
+[□□] 407.30
Y0
Y1
Y2
Y3
Y4
Y5
Y7
Y6

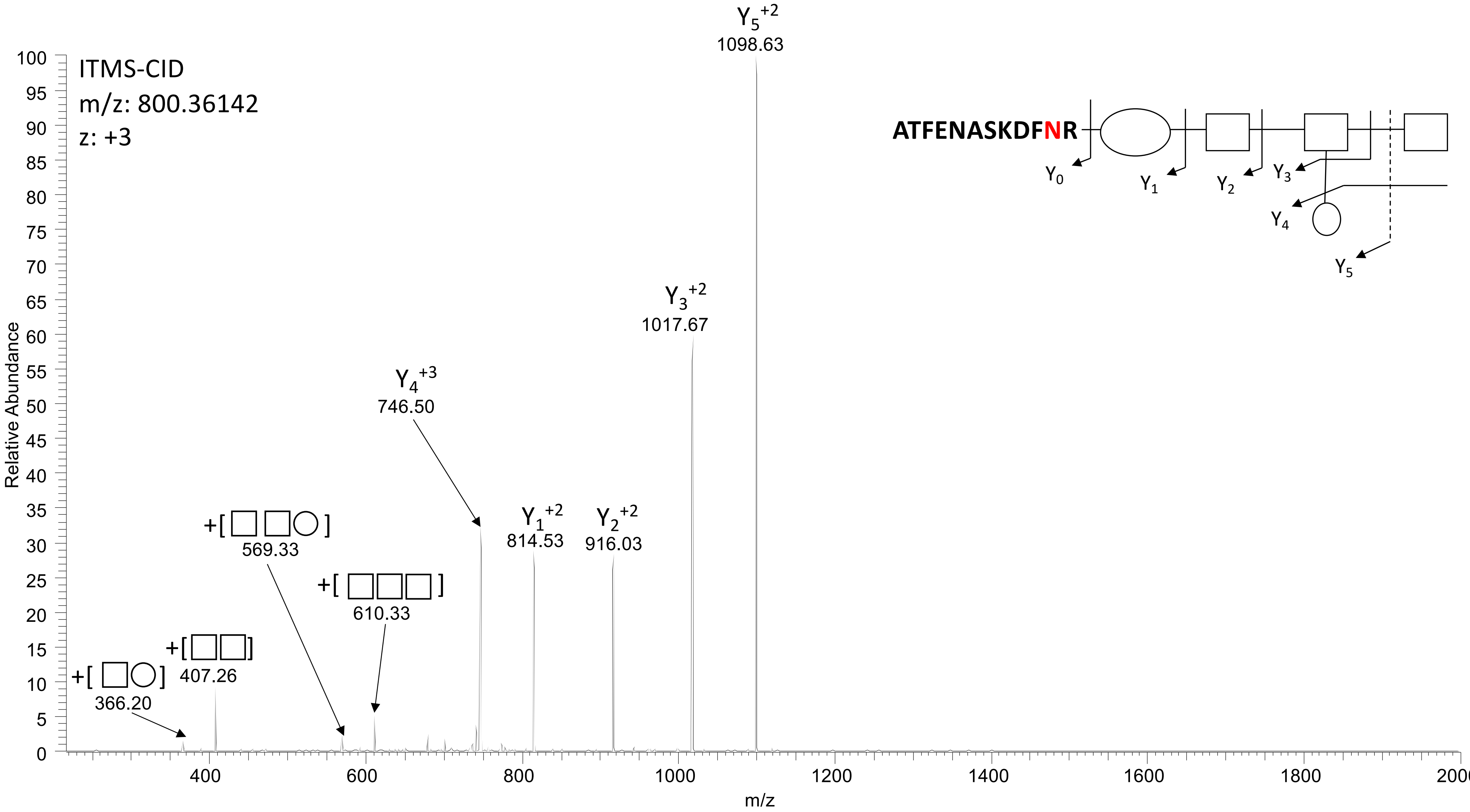
ITMS-CID
m/z: 800.36142
z: +3
ATFENASKDFNR
Y0
Y1
Y2
Y3
Y4
Y5
Y5 +2
1098.63
Y3 +2
1017.67
Y4 +3
746.50
Y1 +2
814.53
Y2 +2
916.03
569.33
610.33
407.26
366.20
Relative Abundance
m/z

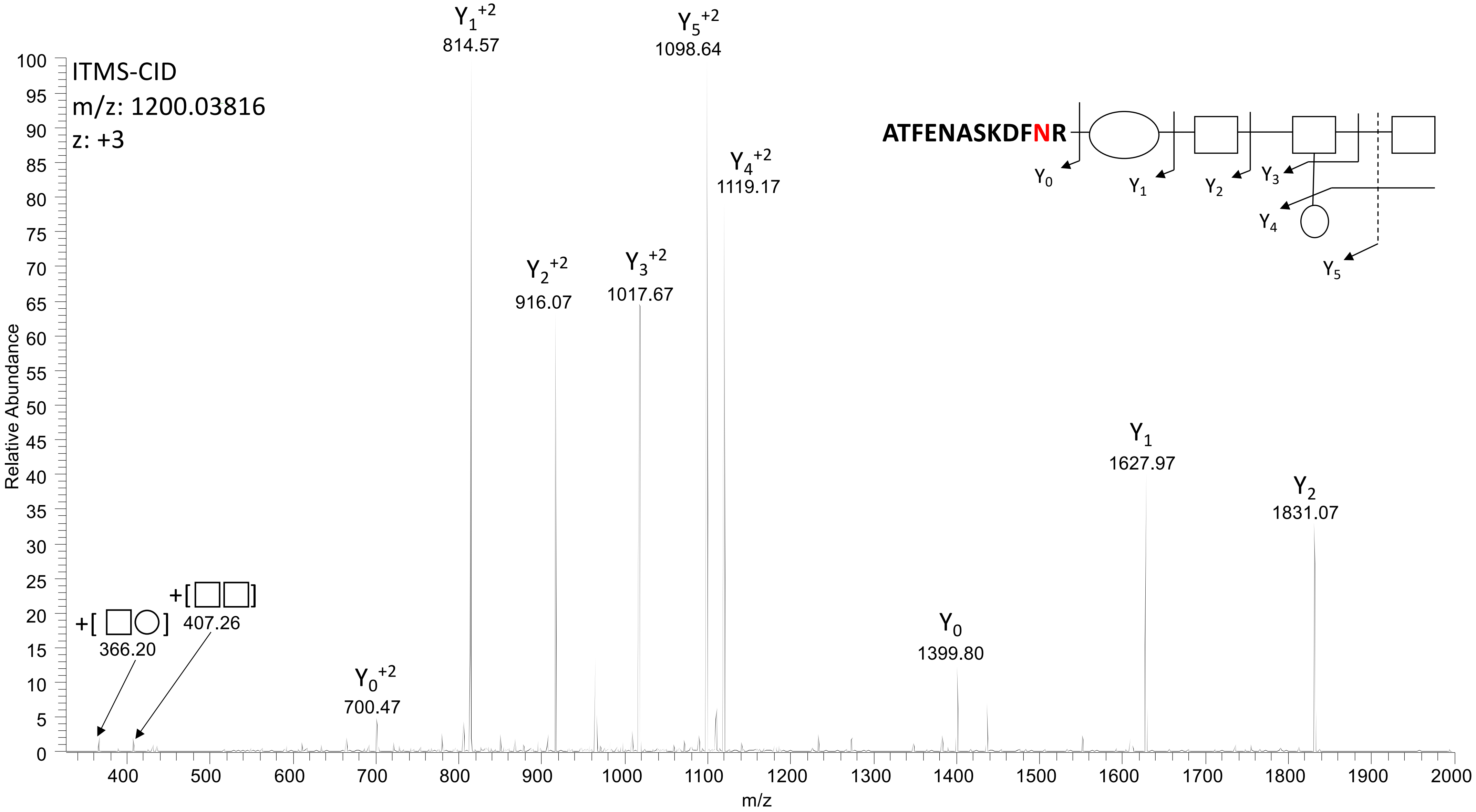
ITMS-CID
m/z: 1200.03816
z: +3
ATFENASKDFNR
Y0
Y1
Y2
Y3
Y4
Y5
Y1+2
814.57
Y5+2
1098.64
Y4+2
1119.17
Y2+2
916.07
Y3+2
1017.67
Y1
1627.97
Y2
1831.07
Y0
1399.80
Y0+2
700.47
+[ ]
366.20
+[ ]
407.26
Relative Abundance
m/z

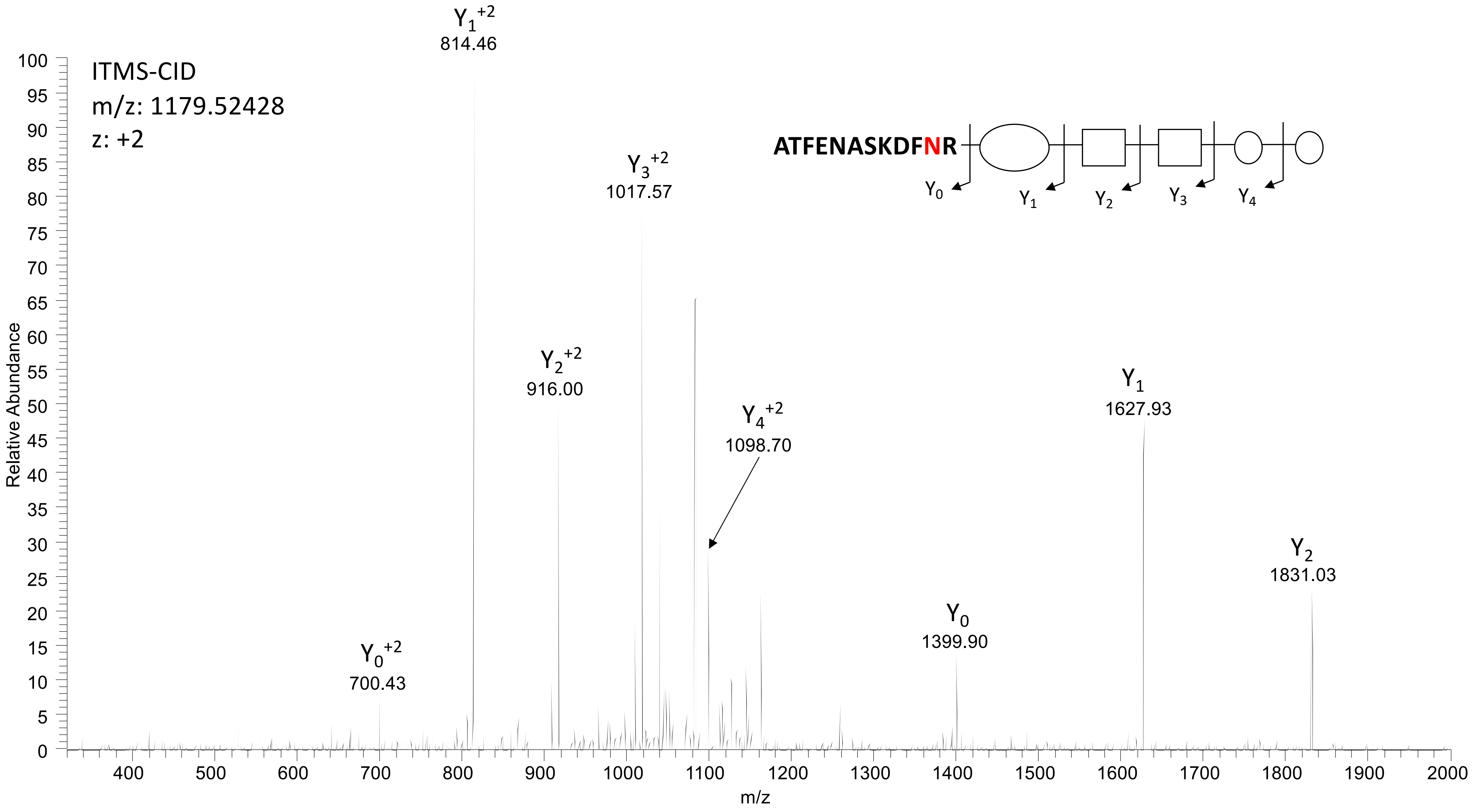

ITMS-CID
m/z: 1179.52428
z: +2
ATFENASKDFNR
Y0
Y1
Y2
Y3
Y4
Y1+2
814.46
Y3+2
1017.57
Y2+2
916.00
Y4+2
1098.70
Y1
1627.93
Y2
1831.03
Y0
1399.90
Y0+2
700.43
Relative Abundance
m/z

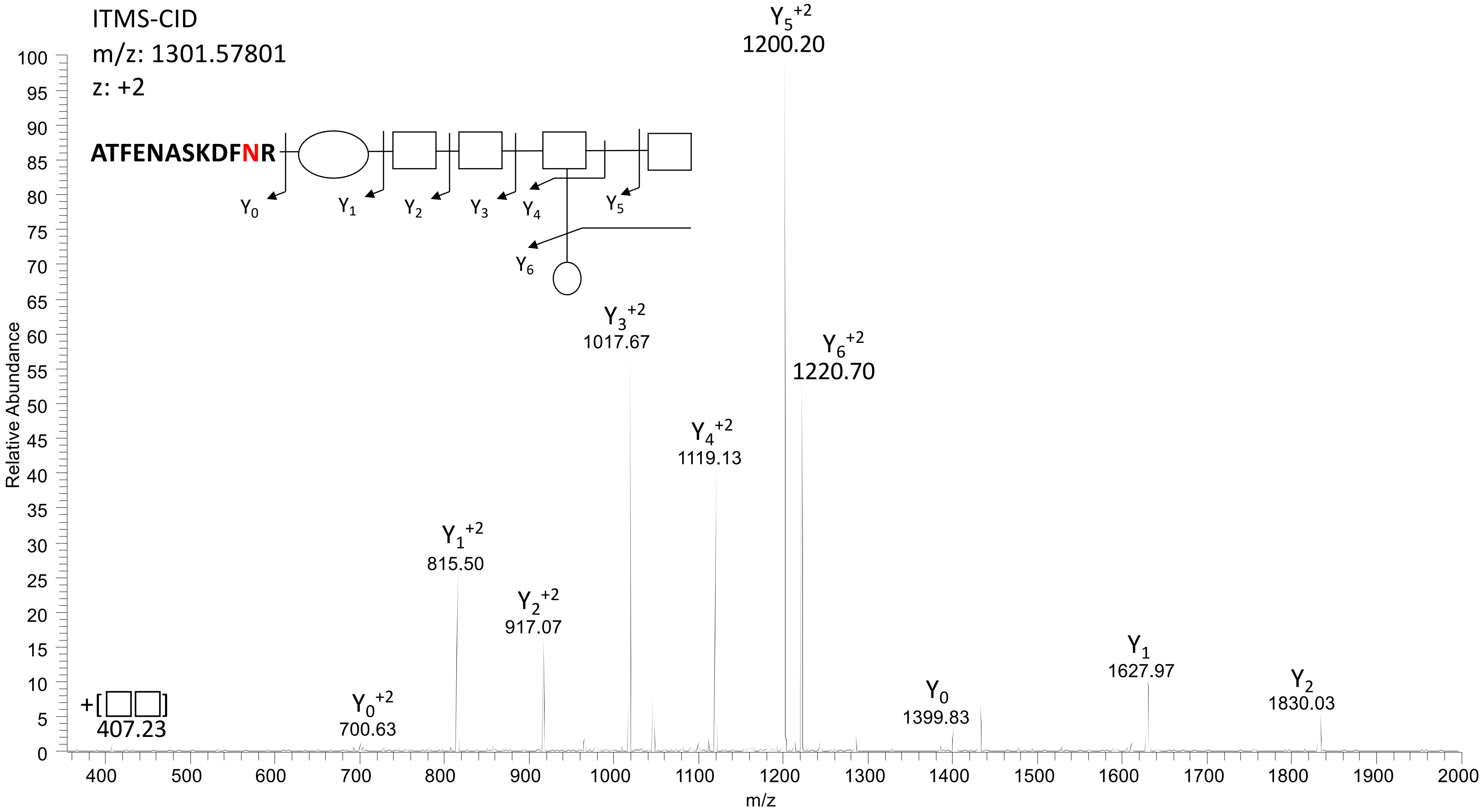
ITMS-CID
m/z: 1301.57801
z: +2
ATFENASKDFNR
Y0 Y1 Y2 Y3 Y4 Y5 Y6
Y5+2 1200.20
Y3+2 1017.67
Y6+2 1220.70
Y4+2 1119.13
Y1+2 815.50
Y2+2 917.07
Y1 1627.97
Y2 1830.03
Y0 1399.83
Y0+2 700.63
+[□□] 407.23
Relative Abundance
m/z

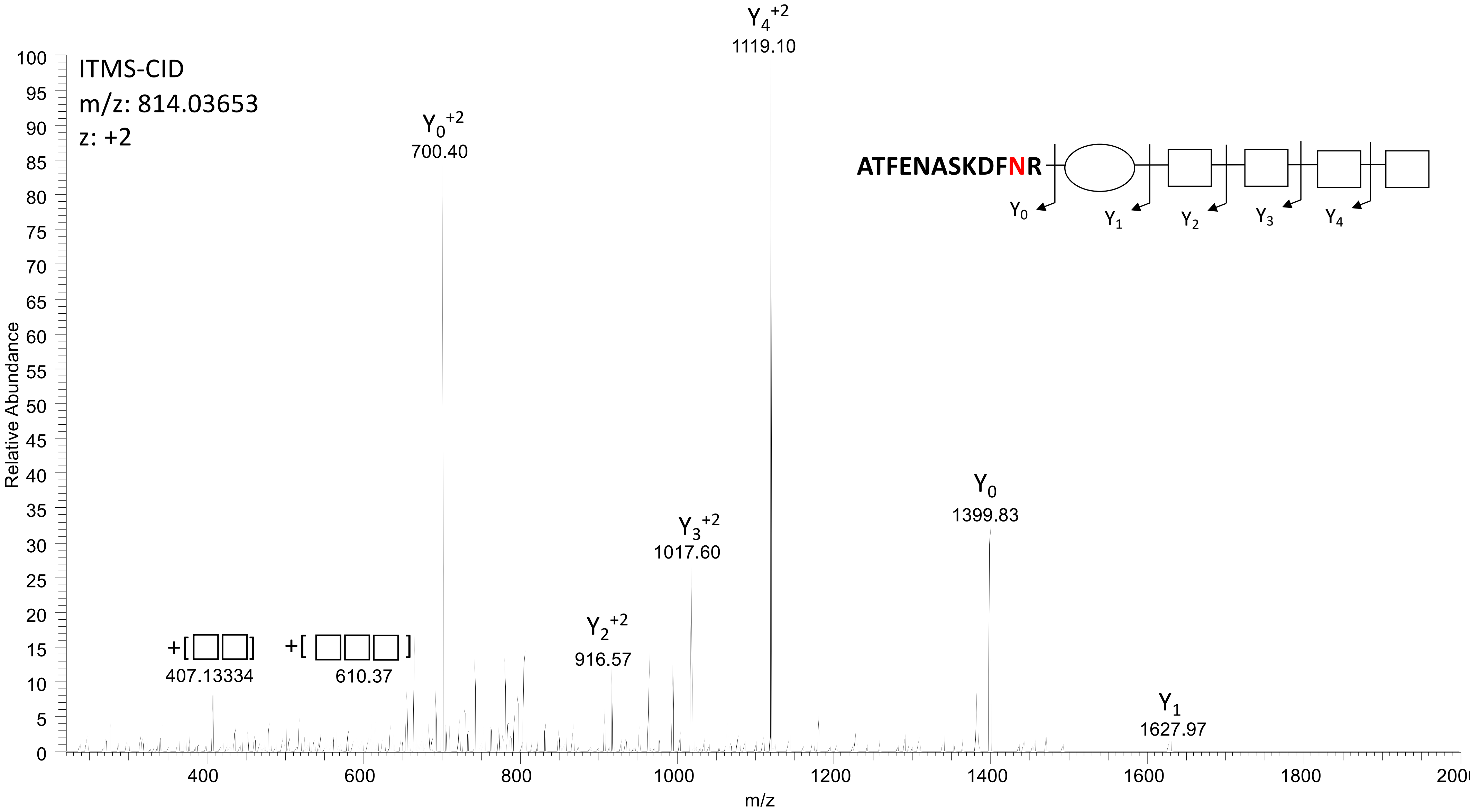

ITMS-CID
m/z: 814.03653
z: +2
Y0+2
700.40
Y4+2
1119.10
ATFENASKDFNR
Y0
Y1
Y2
Y3
Y4
Y3+2
1017.60
Y0
1399.83
Y2+2
916.57
+[ ]
407.13334
+[ ]
610.37
Y1
1627.97
Relative Abundance
m/z

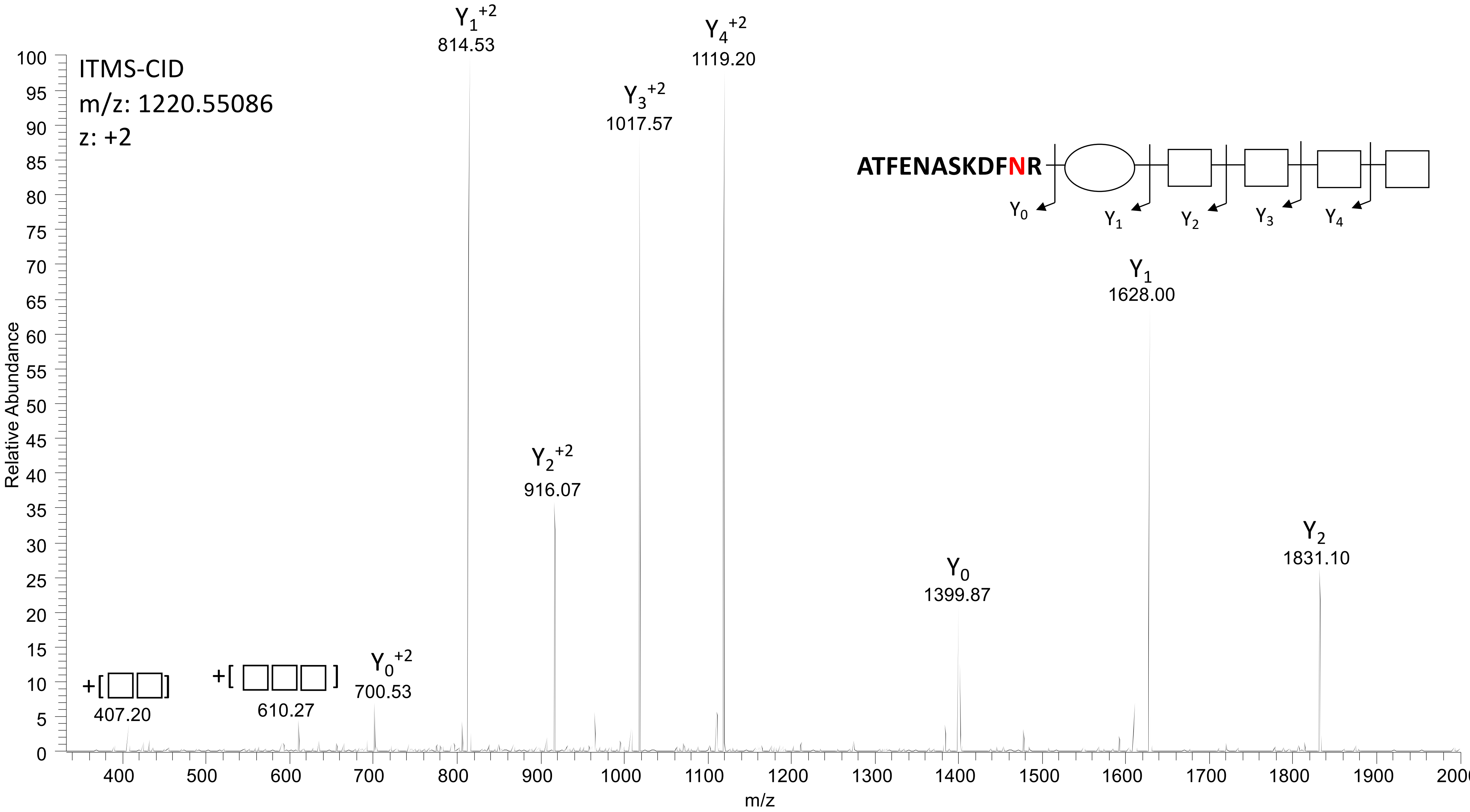
ITMS-CID
m/z: 1220.55086
z: +2
ATFENASKDFNR
Y_0 Y_1 Y_2 Y_3 Y_4
Y_1^{+2} 814.53
Y_4^{+2} 1119.20
Y_3^{+2} 1017.57
Y_1 1628.00
Y_2^{+2} 916.07
Y_2 1831.10
Y_0 1399.87
Y_0^{+2} 700.53
+[□□] 407.20
+[□□□] 610.27
Relative Abundance
m/z

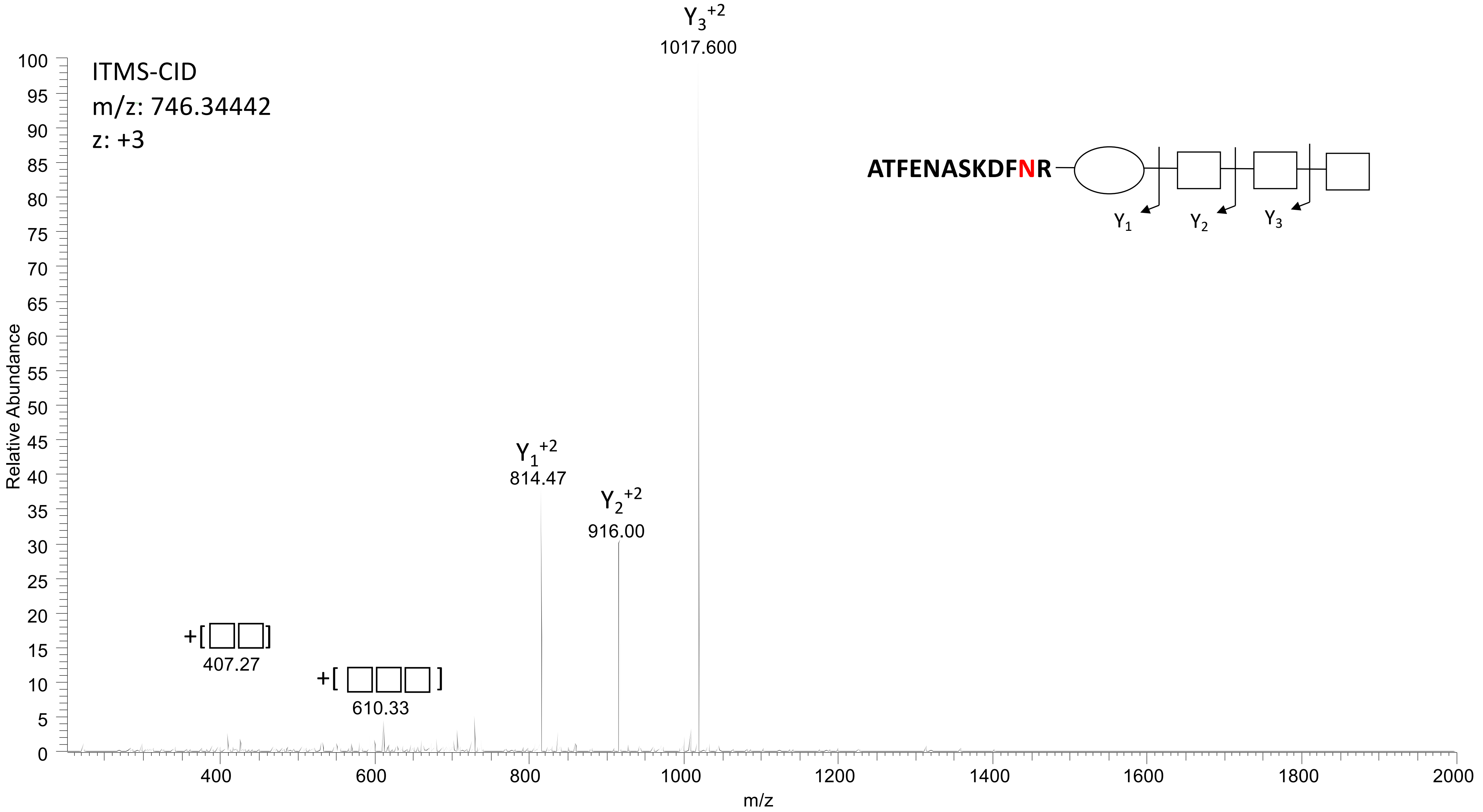
ITMS-CID
m/z: 746.34442
z: +3
ATFENASKDFNR
Y1
Y2
Y3
Y3+2
1017.600
Y1+2
814.47
Y2+2
916.00
+[]
407.27
+[]
610.33
Relative Abundance
m/z
100
95
90
85
80
75
70
65
60
55
50
45
40
35
30
25
20
15
10
5
0
400
600
800
1000
1200
1400
1600
1800
2000

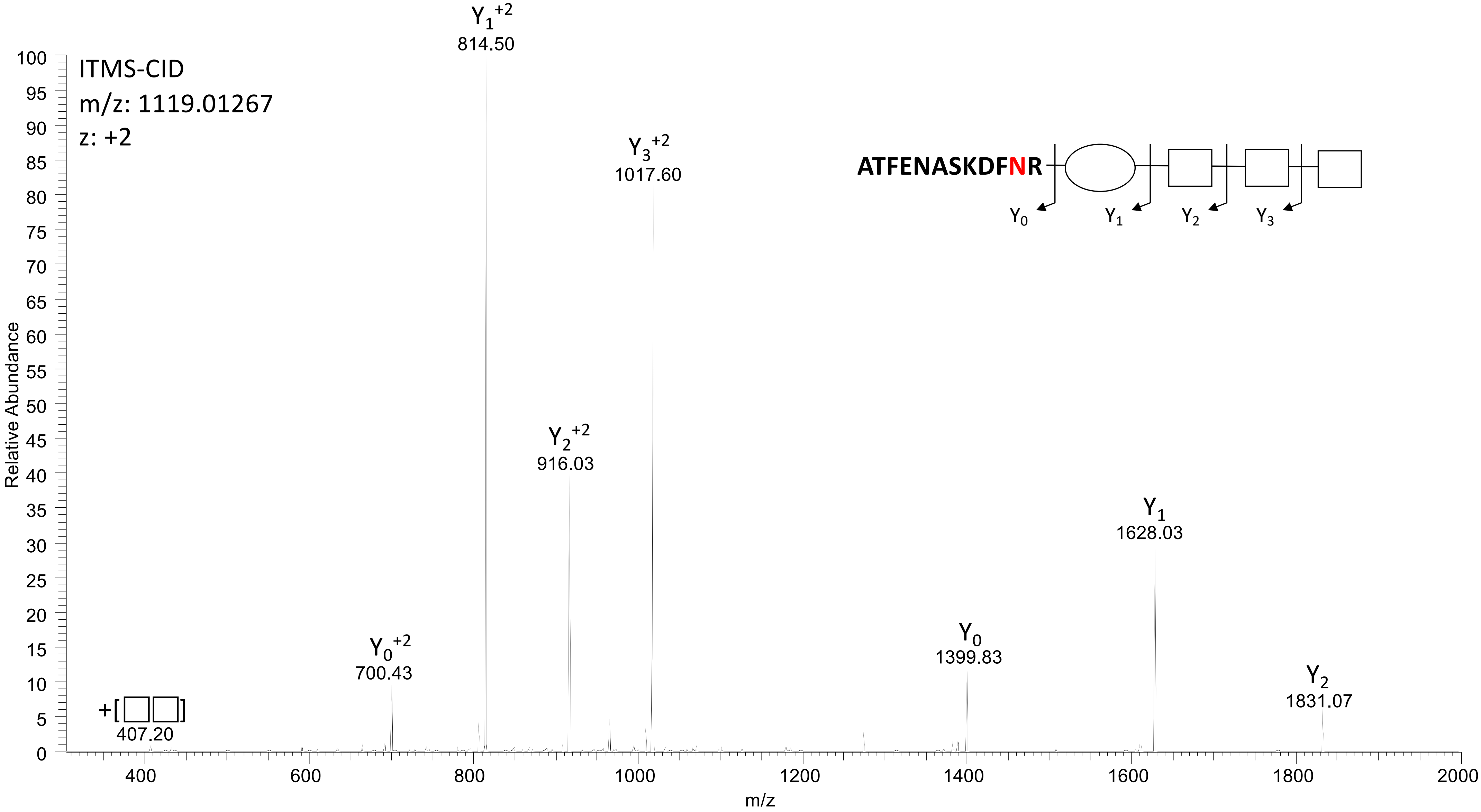
ITMS-CID
m/z: 1119.01267
z: +2
ATFENASKDFNR
Y0
Y1
Y2
Y3
Y1+2
814.50
Y3+2
1017.60
Y2+2
916.03
Y1
1628.03
Y0
1399.83
Y0+2
700.43
Y2
1831.07
+[□□]
407.20
Relative Abundance
m/z
